# Higher Circulating Lymphocytes and the Incidence of Pre-eclampsia and Eclampsia

**DOI:** 10.1155/2024/8834312

**Published:** 2024-03-19

**Authors:** Qiuping Zhao, Rongmei Liu, Hui Chen, Xiaomo Yang, Jiajia Dong, Minfu Bai, MingYang Yu, Zeying Feng, Dingyuan Zeng

**Affiliations:** ^1^Fuwai Central China Cardiovascular Hospital, Zhengzhou, Henan, China; ^2^Department of Gynecology and Obstetrics, Guangxi Clinical Research Center for Obstetrics and Gynecology, Liuzhou Key Laboratory of Gynecologic Oncology, Liuzhou Hospital, Guangzhou Women and Children's Medical Center, Liuzhou, Guangxi, China

## Abstract

Excessive immune activation contributes to the onset of early dysfunction of the maternal-fetal interface, and it is closely linked to the development of pre-eclampsia. However, the effect of specific immune cells on the risk of pre-eclampsia and eclampsia remains controversial. We investigated the causal relationship between immune cells and pre-eclampsia and eclampsia. For exposure, we extracted genetic variants associated with immune cell-related traits, and for outcomes, we used summary genetic data of pre-eclampsia/eclampsia. A two-sample Mendelian randomization (MR) analysis was then performed to assess the causal relationship. Robustness of the MR results was then evaluated through colocalization analysis. We found that genetically proxied circulating lymphocyte absolute count was causally associated with total eclampsia (odds ratio (OR) = 1.53, 95% confidence interval (CI) (1.31-1.79), *p* = 1.15*E* − 07) and pre-eclampsia (OR = 1.50, 95% CI (1.28-1.77), *p* = 9.18*E* − 07); T cell absolute count was causally associated with total eclampsia (OR = 1.49, 95% CI (1.28-1.73), *p* = 2.73*E* − 07) and pre-eclampsia (OR = 1.47, 95% CI (1.25-1.72), *p* = 1.76*E* − 06). And CD28- CD25+ CD8+ T cell absolute count was causally associated with total eclampsia (OR = 1.83, 95% CI (1.44-2.32), *p* = 7.11*E* − 07) and pre-eclampsia (OR = 1.77, 95% CI (1.38-2.26), *p* = 6.55*E* − 06). Colocalization analysis revealed that immune cell-related traits shared the same variant with pre-eclampsia/eclampsia. Our study suggested causal effects of genetic predisposition to high lymphocyte absolute count levels, T cell absolute count, and CD28- CD25+ CD8+ T cell absolute count on eclampsia, particularly pre-eclampsia risk, providing crucial new insights into the potential prevention target for eclampsia and pre-eclampsia.

## 1. Introduction

Pre-eclampsia and eclampsia are two of the most serious acute multisystemic disorders during pregnancy [1] and are significant determinants of maternal and neonatal mortality on a global scale [2, 3]. These conditions affect 3–8% of all pregnancies and contribute to 50,000–60,000 maternal deaths annually worldwide [4]. Pre-eclampsia is associated with an elevated susceptibility to adverse pregnancy outcomes, such as preterm birth and intrauterine growth restriction, thereby amplifying the risk of low birth weight [5]. Furthermore, it is intricately linked to serious maternal and neonatal health complications, including chronic hypertension, maternal end-stage renal disease, and neonatal pulmonary dysplasia [6–8].

The precise etiology of pre-eclampsia remains elusive, although our current understanding suggests that women afflicted with pre-eclampsia exhibit increased uterine artery resistance due to impaired immune regulation. This, in turn, contributes to the activation of the maternal endothelium and the onset of systemic chronic inflammation [9]. Multiple studies have found that immune cells change significantly in women with pre-eclampsia. For example, several studies have found alterations in the relative frequencies of circulating T cell subsets, including CD4+ T cells, CD8+ T cells, and *γδ* T cells [10, 11]. Nevertheless, the majority of preceding investigations were conducted in a cross-sectional manner, thus posing a challenge in establishing any causal relationship between exposure and outcome. Furthermore, confounding factors such as age and environmental influences can confound the association between immune cells and pre-eclampsia. Consequently, the ability to draw causal inferences regarding the role of immune cells in the development of pre-eclampsia was limited.

Mendelian randomization (MR) presents a robust means to investigate the causal relationship between immune cells and pre-eclampsia by genetic variants (single nucleotide polymorphisms (SNPs)) [12], and it is also less susceptible to the shortcomings of classical epidemiological studies, such as confounding bias, information bias, and selection bias [13]. Recently, the application of MR has gained significant traction in elucidating the causal link between immune cells and various diseases such as hypertension [14], amyotrophic lateral sclerosis [15], and multiple sclerosis [16]. In this study, we utilized MR and colocalization analysis to investigate the potential causal association between immune cells and pre-eclampsia.

## 2. Materials and Methods

### 2.1. Study Design

**Figure 1:**
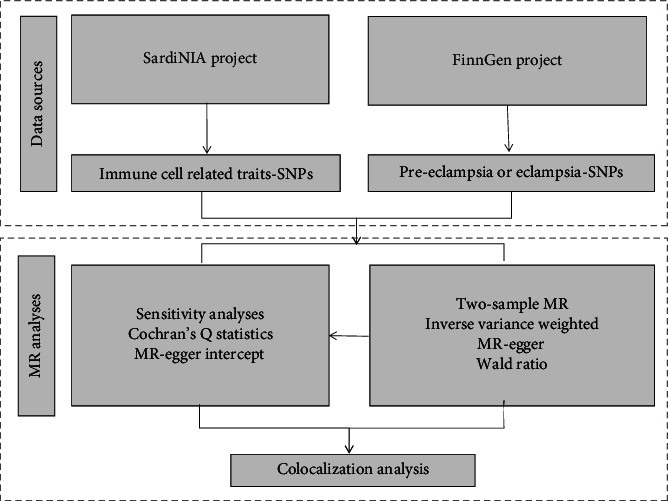
Diagram of the MR framework used in this study.

A comprehensive depiction of the study is presented in Figure 1. First, we selected instrumental variables (IVs) (*p* < 5*E* − 08) for immune cell-related traits and extracted the available summary data from the latest and largest genome-wide association study (GWAS) for pre-eclampsia/eclampsia. Second, we carried out a two-sample MR with a series of sensitivity analyses. Third, we employed a colocalization analysis to assess whether the immune cell-related traits and pre-eclampsia/eclampsia share the same causal SNP in the same specific region.

### 2.2. Data Sources for Two-Sample MR

#### 2.2.1. Selection of Exposure

We collected available GWAS summary data for immune cell-related traits from the SardiNIA project [17] from a relevant subset of 3,757 participants who had undergone immune profiling. A total of 25 ml of blood was collected from each participant and subsequently fractionated to yield serum, EDTA plasma, heparin plasma, white blood cells, and red blood cells. All participants provided informed consent to abide by the study protocols, which were duly approved by the Sardinian Regional Ethics Committee. This specific substudy reported about 22 million variants on a total of 731 immune cell traits, including 118 absolute cell counts, 192 relative counts (ratios between cell levels), 389 median fluorescence intensities of surface antigens, and 32 morphological parameters [18]. GWAS summary data for the immune cell-related traits are available at https://www.ebi.ac.uk/gwas/publications/32929287. More patient demographic information for the SardiNIA project is shown in Table S1.

#### 2.2.2. Selection of Outcomes

We assembled aggregated data on pre-eclampsia/eclampsia from FinnGen, a major public-private partnership set up to capture and analyze genetic and health data on over half a million people. The discharge and cause of death registries in FinnGen diagnose women with pre-eclampsia/eclampsia based on the International Classification of Diseases (ICD-10: O14 and ICD-8: 6370). In total, 6,436 cases and 176,113 controls were identified (https://www.finngen.fi/en/access_results).

The participants shared a homogeneous genetic background characterized by European ancestry, and there was no overlap in samples between immune cell traits and GWASs conducted for pre-eclampsia/eclampsia.

#### 2.2.3. SNP Selection

We selected SNPs that were found to be robustly associated with immune cell traits as IVs (*p* < 5*E* − 08). To ensure conditional independence, we established two stringent criteria for selection (LD threshold of *r*^2^ < 0.001 and a minimum distance of 10,000 kb between them). The *F*-statistic was utilized as a metric to quantify the strength of the association between the IVs and the exposures of interest.

### 2.3. Statistical Analysis

#### 2.3.1. Two-Sample Mendelian Randomization

In order to assess the causal impact of immune cell traits on pre-eclampsia/eclampsia, we employed a conventional MR framework. For an instrumental IV to be considered valid, it must satisfy three essential assumptions: (1) truly associated with immune cell traits (defined here as *p* < 5*E* − 08), (2) not associated with confounders of immune cell traits and pre-eclampsia/eclampsia, and (3) only associated with pre-eclampsia/eclampsia through immune cell traits (Figure S1).

To examine the causal impacts of immune cell traits on the occurrence of pre-eclampsia/eclampsia, we utilized the inverse-variance-weighted (IVW) method [19], using the Wald ratio when only a single SNP was available. The IVW method was chosen for its recognized statistical potency in the presence of valid IVs that can yield robust estimates of causal effects.

#### 2.3.2. Sensitivity Analysis

In order to assess whether there were any violations of the three IV assumptions, we employed Cochran's *Q*-test to evaluate the heterogeneity of the results [20]. Additionally, the MR-Egger intercept test was used to detect potential indications of horizontal pleiotropy, where an intercept *p* value > 0.05 suggests that there is no horizontal pleiotropy [21]. We took the Wald ratio or IVW results as the primary associations (adjusted *p* value < 0.05 after Bonferroni corrections for multiple comparisons by 0.05/731).

#### 2.3.3. Colocalization Analysis

Colocalization analysis is a statistical method to investigate whether two distinct genetic traits share a common underlying causal variable [22]. Briefly, colocalization analysis revolves around five key hypotheses: H0, no association with either trait; H1, association with trait 1 but not with trait 2; H2, association with trait 2 but not with trait 1; H3, association with both traits 1 and 2, with 2 independent SNPs; and H4, association with both traits 1 and 2, with a single shared SNP. In this analysis, posterior probability for H4 (PPH4) exceeding 95% means that the gene expressions and pre-eclampsia/eclampsia are not only associated but also share a common causal SNP.

All MR analysis was performed in R (version 4.2.0) with the R packages “TwoSampleMR” and “coloc.” Results are presented as odds ratios with respective 95% confidence intervals (CIs).

## 3. Results

### 3.1. Exposure and Outcome

Information on immune cell traits and pre-eclampsia/eclampsia GWAS datasets in the MR study is shown in Table 1. In total, we used 2,481 SNPs as IVs for 605 immune cell traits (*F*-statistics: 15.7-996.1). Power calculation results can be found in Supplementary Table S2.

### 3.2. Main Findings

**Figure 2:**
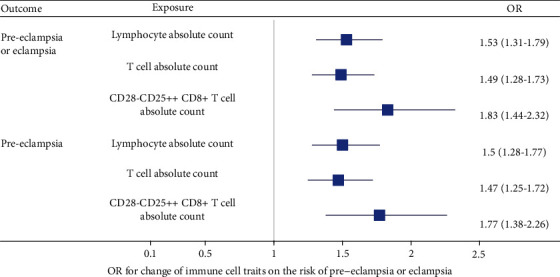
The association between genetically determined immune cell traits and the risk of pre-eclampsia/eclampsia. OR: odds ratio.

Table 2 shows the MR results for the effects of immune cell traits on pre-eclampsia/eclampsia. Genetically determined lymphocyte absolute count was causally associated with total eclampsia (OR = 1.53, 95% CI (1.31-1.79), *p* = 1.15*E* − 07) and pre-eclampsia (OR = 1.50, 95% CI (1.28-1.77), *p* = 9.18*E* − 07) in IVW; genetically determined T cell absolute count was causally associated with total eclampsia (OR = 1.49, 95% CI (1.28-1.73), *p* = 2.73*E* − 07) and pre-eclampsia (OR = 1.47, 95% CI (1.25-1.72), *p* = 1.76*E* − 06) in IVW; and CD28- CD25+ CD8+ T cell absolute count was causally associated with total eclampsia (OR = 1.83, 95% CI (1.44-2.32), *p* = 7.11*E* − 07) and pre-eclampsia (OR = 1.77, 95% CI (1.38-2.26), *p* = 6.55*E* − 06) in terms of the Wald ratio (Figure 2). There was little evidence for causal effects for other immune cell traits on total pre-eclampsia/eclampsia, single pre-eclampsia, and single eclampsia (Table S3, S4, and S5), and there was no heterogeneity for immune cell traits according to Cochran's *Q*-test (Table 2). The MR-Egger intercept test showed no results due to too few SNPs.

### 3.3. Colocalization Analysis

Colocalization analysis revealed that circulating lymphocytes (PPH4 = 0.952), T cells (PPH4 = 0.953), and CD28- CD25+ CD8+ T cells (PPH4 = 0.999) shared the same variants with pre-eclampsia and eclampsia (Table 3).

## 4. Discussion

In this study, we aimed to unravel the underlying genetic architecture that elucidates the observed association between immune cell characteristics and the occurrence of pre-eclampsia/eclampsia. Our findings collectively demonstrate significant associations between genetically predicted lymphocyte and T cell count and the risk of pre-eclampsia, as well as the combined occurrence of pre-eclampsia and eclampsia.

A complex interplay of acquired, genetic, and immune risk factors collectively contributes to the onset of early placental dysfunction, and many researchers believe that an abnormal maternal immune response to the fetus is the initiating factor in the development of eclampsia. Moreover, this process involves cells of the innate and adaptive immune systems, including neutrophils, monocytes, natural killer (NK) cells, and T lymphocytes. This dysfunction also triggers the release of antiangiogenic factors ultimately culminating in subsequent multiorgan dysfunction [23]. Previous research has indicated that T cell subset composition and activation status at the uteroplacental interface are comparable to that found in the peripheral blood [24]. Additionally, immune cells in the peripheral blood have been shown to be associated with eclampsia and are more susceptible to monitoring and intervention, so we chose to assess the causal relationship between peripheral immune cell-related traits and the risk of pre-eclampsia/eclampsia.

Previous studies have shown that abnormalities in lymphocytes and pre-eclampsia often occur together [25]. A recent study investigated the involvement of Treg cells and Th1/Th2/Th17 cytokines in both healthy and pathological pregnancies [26] and found a noteworthy reduction in anti-inflammatory cytokines, namely, IL-10 and IL-4, in addition to a substantial elevation of proinflammatory cytokines such as IL-17 and IL-2 in individuals affected by pre-eclampsia. The mobilization of Tregs from peripheral blood to the decidua is imperative for preserving maternal-fetal immune tolerance, underscoring the significant involvement of T cells in the development of eclampsia [26]. We also found that CD28- CD25+ CD8+ T cell absolute count, a subset of T cells with immunomodulatory effects, was associated with total eclampsia and pre-eclampsia in the present study. CD25+ CD8+ T cells, a subset of CD8+ cells with immune-modulatory functions [27], have rarely been studied in general, and their specific immune function and role in eclampsia remain unclear. Only one observational clinical study has found a significant increase in peripheral blood CD8+CD25+ T cells in eclampsia, suggesting a possible mechanism for their involvement in eclampsia pathogenesis [28].

Previous research has also reported increased quantities and activity of CD8 T cells in peripheral blood during pre-eclampsia [11, 29], which aligns with our results. However, phenotypic changes in effector T cells from women with pre-eclampsia include the increased expression of activation-associated markers such as HLA-DR on CD8+ T cell subsets [30]. The levels of IL-17, IL-2, TNF, and IFN-*γ* secreted by CD8 T cells are also significantly elevated in pre-eclampsia [25, 31], indicating the activation of both the innate and adaptive immune responses in the pathogenesis of the pronounced maternal systemic inflammation observed in pre-eclampsia. This suggests the involvement of multiple components of the immune system in the development of the heightened inflammatory state observed in pre-eclampsia.

The key role of lymphocytes in the development and progression of pre-eclampsia and eclampsia is not fully understood, but the following mechanisms may be involved. Data from animal experiments suggest that the role of lymphocytes may be mediated through regulation of vascular function, sympathetic outflow, and antigen-presenting cells in renal sodium reabsorption and salt handling [32, 33]. Furthermore, in the pathogenesis of pre-eclampsia, there is a transition from Tregs to Th17 cells that results in an aberrant immune milieu that triggers inflammation and compromises immune tolerance [34, 35]. Immunomodulatory therapies that target T cells have been investigated in a rat model of pre-eclampsia and have been found to have the potential to decrease the generation of proinflammatory mediators such as TNF while stimulating T+H2 cell differentiation [36]. In addition, preparations of immunoglobulins that can reduce cytotoxic T cell have recently become available for clinical use including treatment of various immunologic disorders during pregnancy [37]. Given their low cost of analysis and high accessibility, lymphocyte and T cell count parameters might be ideal potential biomarkers in the early identification of high-risk pre-eclampsia patients. Future studies on the role of T cell subsets, especially CD28- CD25+ CD8+ T cells, in the pathogenesis of eclampsia may also identify new targets for clinical intervention.

A major strength of our study is that we used a comprehensive and complementary genetic approach to evaluate genetic causality between lymphocyte and T cell counts and the combined risk of pre-eclampsia and eclampsia in addition to employing a variety of sensitivity analyses to quantify the consistency and robustness of our results. Moreover, by leveraging the extensive pooled data from a large population, our study successfully confirmed the presence of independent SNPs associated with the exposure of interest, thereby yielding enhanced statistical power. More importantly, we used multiple models with different model assumptions to prevent false positives due to misspecification.

However, this study has several limitations. First, we mostly included participants of European descent from Sardinia, who have a unique genetic heritage [38], and thus, our results may not be generalizable to other, genetically distinct populations. Second, the GWAS sample size for eclampsia in this study was relatively small. Therefore, the conclusions may not be representative of the general global population. Third, we only considered pre-eclampsia and eclampsia as outcome traits and did not examine the mechanisms behind our findings; more clinical research is needed to validate our findings. Finally, even though we used recent GWAS pooled data for exposure-related traits, we overlooked several unknown SNPs associated with immune cell traits in our analysis.

## 5. Conclusions

This study demonstrated a causal relationship between lymphocyte and T cell count and pre-eclampsia and the combination of pre-eclampsia and eclampsia. Based on these findings, we suggest that routine blood examinations should be incorporated into the clinical evaluation of pregnant woman more frequently. In addition, lymphocyte and T cell counts should be monitored in patients with pre-eclampsia and eclampsia. However, additional investigations are imperative to corroborate and validate these findings, in order to evaluate their robustness and generalizability.

## Figures and Tables

**Table 1:** Characteristics of immune cell traits and pre-eclampsia/eclampsia datasets.

Phenotype	Data source	Sample size (cases)	Population	Year of publication	PubMed ID/GWAS ID
*Exposures*					
Immune cell traits	Valeria Orrù	3757	European	2020	PMID: 32929287
*Outcomes*					
Pre-eclampsia/eclampsia	FinnGen	182,549 (6436)	European	2021	finn-b-O15_PRE_OR_ECLAMPSIA
Pre-eclampsia	FinnGen	182,035 (5922)	European	2021	finn-b-O15_PREECLAMPS
Eclampsia	FinnGen	176,539 (426)	European	2021	finn-b-O15_ECLAMPSIA

SNP: single nucleotide polymorphism; *F* > 10 in MR analysis means there was significant association between IV and immune cell traits.

**Table 2:** Two-sample Mendelian randomization estimations showing the effects of immune cell traits on the risk of pre-eclampsia/eclampsia.

Outcomes	Exposures	Inverse-variance weighted			
		OR (95% CI)	*p* value	*Q*-statistic	*p* value^a^
Pre-eclampsia/eclampsia	Lymphocyte absolute count	1.53 (1.31-1.79)	1.15*E*-07	0.001	9.87*E*-01
	T cell absolute count	1.49 (1.28-1.73)	2.73*E*-07	0.038	8.46*E*-01
	CD28- CD25++ CD8+ T cell absolute count	1.83 (1.44-2.32)	7.11*E*-07	NA	NA

Pre-eclampsia	Lymphocyte absolute count	1.50 (1.28-1.77)	9.18*E*-07	0.007	9.34*E*-01
	T cell absolute count	1.47 (1.25-1.72)	1.76*E*-06	0.096	7.56*E*-01
	CD28- CD25++ CD8+ T cell absolute count	1.nn (1.38-2.26)	6.55*E*-06	NA	NA

OR: odds ratio; CI: confidence interval. ^a^*p* value < 0.05 indicates the presence of heterogeneity. ^b^*p* value < 0.05 indicates the presence of horizontal pleiotropy.

**Table 3:** Colocalization analysis showing the immune cell-related traits and pre-eclampsia/eclampsia GWAS associations shared a causal SNP in the given region.

Outcome	Exposures	PPH0	PPH1	PPH2	PPH3	PPH4
Pre-eclampsia/eclampsia	Lymphocyte absolute count	5.26*E*-04	4.71*E*-02	1.06*E*-05	0	9.52*E*-01
	T cell absolute count	9.21*E*-06	4.71*E*-02	1.86*E*-07	0	9.53*E*-01
	CD28- CD25++ CD8+ T cell absolute count	3.88*E*-06	5.35*E*-04	7.24*E*-06	0	9.99*E*-01

PPH: posterior probability of hypothesis; PPH4 > 95% indicates that the immune cell-related traits and pre-eclampsia/eclampsia share a single causal SNP.

## Data Availability

The datasets were derived from sources in the public domain: FinnGen consortium (https://www.finngen.fi/en/access_results/) and ieu (https://www.ebi.ac.uk/gwas/publications/32929287).

## Abbreviations

GWAS: Genome-wide association studies
IV: Instrumental variable
MR: Mendelian randomization
SNP: Single nucleotide polymorphism.

## Supplementary Materials

Supplementary Materials Table S1: patient demographic information from SardiNIA. Table S2: statistical power calculation for MR analysis. SNP: single nucleotide polymorphism; OR: odds ratio. Table S3: two-sample MR estimations showing the effects of immune cell traits on the risk of pre-eclampsia/eclampsia. Table S4: two-sample MR estimations showing the effects of immune cell traits on the risk of pre-eclampsia. Table S5: two-sample MR estimations showing the effects of immune cell traits on the risk of eclampsia. Figure S1: IV assumptions for MR.
