# Supplementary material for: Higher Circulating Lymphocytes and the Incidence of Pre-eclampsia and Eclampsia
Source: J Pregnancy. 2024 Mar 19;2024:8834312. doi: 10.1155/2024/8834312 (PMC10965280; doi:10.1155/2024/8834312)

**Table S1.Patient demographic information of SardiNIA**

|  |  | **Overall** | | **Males** | | | | **Females** | | | |
| --- | --- | --- | --- | --- | --- | --- | --- | --- | --- | --- | --- |
| Age Band |  |  |  | 14-29 yrs | 30 - 44 yrs | 45 - 59 yrs | 60- 102 yrs | 14-29 yrs | 30 - 44 yrs | 45 - 59 yrs | 60- 102 yrs |
| Mean and SD of sample size |  | 6111 | (43.6) | 688.3 | 743.0 | 611.7 | 564.2 | 904.8 | 1049.6 | 851.3 | 698.2 |
| **Anthropometric** Measures |  |  |  |  |  |  |  |  |  |  |  |
| Height | cm | 160.0 | (9.1) | 169.0 | 169.0 | 165.0 | 161.0 | 158.0 | 157.0 | 153.0 | 150.0 |
| Weight | kg | 64.9 | (13.3) | 66.4 | 75.1 | 76.0 | 73.0 | 53.4 | 57.8 | 63.2 | 64.0 |
| Waist | cm | 84.8 | (13.1) | 80.5 | 89.4 | 95.0 | 97.7 | 72.2 | 77.1 | 84.9 | 92.0 |
| HIP | cm | 97.6 | (8.3) | 93.8 | 98.0 | 98.9 | 99.0 | 93.3 | 96.3 | 101.0 | 102.0 |
| BMI, body mass index | kg/m2 | 25.3 | (4.7) | 23.1 | 26.1 | 27.8 | 28.2 | 21.3 | 23.5 | 26.8 | 28.5 |
| **Cardiovascular Function** |  |  |  |  |  |  |  |  |  |  |  |
| systolic BP | mmHg | 126 | (18.50) | 121 | 125 | 135 | 144 | 111 | 114 | 129 | 140 |
| diastolic BP | mmHg | 77 | (10.90) | 71 | 79.2 | 84.9 | 83.9 | 68.4 | 72.8 | 80 | 81.7 |
| HR (heart rate) | beats/min | 67.1 | (11.20) | 64.8 | 63.8 | 65.7 | 64.2 | 70.1 | 68.7 | 68.5 | 68.8 |
| diam_S | mm | 5.9 | (0.70) | 6 | 6.2 | 6.3 | 6.6 | 5.5 | 5.5 | 5.7 | 6.2 |
| diam_D | mm | 5.4 | (0.70) | 5.3 | 5.6 | 5.8 | 6.2 | 4.9 | 5.1 | 5.2 | 5.7 |
| IMT (intimal medial thickness) | mm | 0.6 | (0.10) | 0.5 | 0.5 | 0.6 | 0.7 | 0.5 | 0.5 | 0.6 | 0.7 |
| PWV (pulse wave velocity) | cm/s | 671 | (226.00) | 513 | 623 | 741 | 925 | 495 | 589 | 721 | 923 |
| **Blood Test Analyses** |  |  |  |  |  |  |  |  |  |  |  |
| RBC | 10^6^/uL | 4.8 | (0.6) | 5.3 | 5.2 | 5.1 | 4.9 | 4.6 | 4.6 | 4.7 | 4.7 |
| Hb | g/dL | 13.8 | (1.5) | 14.9 | 14.9 | 14.9 | 14.7 | 13.0 | 12.8 | 13.1 | 13.4 |
| MCV (mean RBC vol) | fL | 86.5 | (9.3) | 85.0 | 86.5 | 88.0 | 90.2 | 85.0 | 85.4 | 86.0 | 87.6 |
| MCH (mean RBC Hb) | pg | 28.8 | (3.6) | 28.6 | 29.1 | 29.4 | 30.1 | 28.3 | 28.4 | 28.5 | 29.0 |
| WBC | 10^3^/uL | 6.7 | (1.7) | 7.1 | 7.0 | 7.1 | 6.9 | 6.7 | 6.5 | 6.2 | 6.2 |
| NE (neutrophils) | % | 56.6 | (8.7) | 54.6 | 56.6 | 56.1 | 57.5 | 55.1 | 58.3 | 57.2 | 57.0 |
| LY (lymphocytes) | % | 34.5 | (7.9) | 35.8 | 34.3 | 34.3 | 32.4 | 36.7 | 33.7 | 34.2 | 34.2 |
| MO (monocytes) | % | 5.9 | (2.3) | 6.6 | 6.0 | 6.3 | 6.7 | 5.6 | 5.3 | 5.6 | 5.8 |
| EO (eosinophils) | % | 2.6 | (1.8) | 2.7 | 2.7 | 2.9 | 3.0 | 2.3 | 2.3 | 2.6 | 2.6 |
| BA (basophils) | % | 0.4 | (0.3) | 0.4 | 0.3 | 0.3 | 0.3 | 0.4 | 0.4 | 0.4 | 0.4 |
| HbA1C | % | 5.4 | (0.8) | 5.2 | 5.3 | 5.8 | 5.9 | 5.1 | 5.2 | 5.5 | 5.9 |
| SERUM GLUCOSE | mg/dL | 90.1 | (23.7) | 84.7 | 89.8 | 100.0 | 104.0 | 79.0 | 82.9 | 90.4 | 100.0 |
| SERUM INSULIN | micro/UmL | 8.5 | (8.1) | 7.4 | 8.1 | 9.8 | 9.0 | 8.3 | 7.6 | 8.8 | 9.8 |
| BUN | mg/dL | 35.0 | (10.4) | 34.8 | 37.6 | 37.5 | 40.9 | 28.4 | 31.5 | 34.8 | 39.9 |
| SERUM CREATININE | mg/dL | 0.8 | (0.2) | 0.9 | 0.9 | 0.9 | 1.0 | 0.7 | 0.7 | 0.7 | 0.8 |
| ALT, ala aminotransferase | U/L | 25.0 | (23.1) | 26.6 | 37.2 | 34.1 | 28.6 | 16.0 | 17.9 | 22.5 | 24.8 |
| AST, asp aminotransferase | U/L | 21.6 | (14.6) | 22.2 | 24.4 | 25.5 | 25.8 | 17.2 | 17.4 | 20.7 | 24.2 |
| Gammagt, γ-glu-transferase | U/L | 27.9 | (39.9) | 22.0 | 41.1 | 51.2 | 46.7 | 13.8 | 15.8 | 21.9 | 27.7 |
| CHOLESTEROL | mg/dL | 208.0 | (42.2) | 175.0 | 213.0 | 226.0 | 220.0 | 185.0 | 204.0 | 226.0 | 228.0 |
| HDL | mg/dL | 64.1 | (14.9) | 55.6 | 57.1 | 59.7 | 62.6 | 66.3 | 68.3 | 69.7 | 69.0 |
| LDL | mg/dL | 127.0 | (35.4) | 105.0 | 134.0 | 141.0 | 136.0 | 106.0 | 121.0 | 139.0 | 140.0 |
| TRIGLYCERIDES | mg/dL | 88.1 | (68.3) | 72.5 | 111.0 | 127.0 | 106.0 | 61.8 | 69.9 | 84.8 | 95.4 |

Abbreviations: diam_D, diastolic diameter; diam_S, systolic diameter;

**Table S2. Statistical power calculation for Mendelian randomization analyses.**

|  |  |  | **Statistical power at the given odds ratio** | | | | | | |  |
| --- | --- | --- | --- | --- | --- | --- | --- | --- | --- | --- |
| **Exposure** | **Sample size** | **Cases** | **OR = 0.80** | **OR = 0.90** | **OR = 1.00** | **OR =1.10** | **OR 1.20** | **OR = 1.30** | **OR = 1.40** | **OR = 1.50** |
| Immune cell traits | 182,549 | 6436 | 1.00 | 0.66 | 0.05 | 0.65 | 1.00 | 1.00 | 1.00 | 1.00 |

SNP: OR: odds ratio

**Table S3. Two-sample Mendelian randomization estimations showing the effects of Immune cell traits on the risk of pre-eclampsia or eclampsia.**

| outcome | exposure | method | nSNP | pval | OR |
| --- | --- | --- | --- | --- | --- |
| Pre-eclampsia or eclampsia | Lymphocyte Absolute Count | Inverse variance weighted | 2 | 1.15E-07 | 1.53 (1.31 -1.79 ) |
|  | T cell Absolute Count | Inverse variance weighted | 2 | 2.73E-07 | 1.49 (1.28 -1.73 ) |
|  | CD28- CD25++ CD8+ T cell Absolute Count | Wald ratio | 1 | 7.11E-07 | 1.83 (1.44 -2.32 ) |
|  | HLA DR on Dendritic Cell | Inverse variance weighted | 7 | 3.32E-04 | 1.07 (1.03 -1.11 ) |
|  | Central Memory CD4+ T cell Absolute Count | Wald ratio | 1 | 8.35E-04 | 1.46 (1.17 -1.82 ) |
|  | CD4+ T cell Absolute Count | Wald ratio | 1 | 8.35E-04 | 1.39 (1.15 -1.69 ) |
|  | FSC-A on CD4+ T cell | Inverse variance weighted | 3 | 9.67E-04 | 0.72 (0.60 -0.88 ) |
|  | CD64 on CD14- CD16- | Inverse variance weighted | 3 | 1.45E-03 | 1.20 (1.07 -1.35 ) |
|  | Terminally Differentiated CD4+ T cell %CD4+ T cell | Inverse variance weighted | 4 | 1.57E-03 | 0.81 (0.71 -0.92 ) |
|  | HLA DR on myeloid Dendritic Cell | Inverse variance weighted | 6 | 1.96E-03 | 1.07 (1.03 -1.12 ) |
|  | CD8+ T cell Absolute Count | Wald ratio | 1 | 2.08E-03 | 1.34 (1.11 -1.62 ) |
|  | CD8+ T cell %leukocyte | Wald ratio | 1 | 2.08E-03 | 1.36 (1.12 -1.66 ) |
|  | CD127- CD8+ T cell Absolute Count | Wald ratio | 1 | 2.08E-03 | 1.34 (1.11 -1.61 ) |
|  | CD45 on Natural Killer T | Wald ratio | 1 | 2.94E-03 | 1.41 (1.12 -1.77 ) |
|  | CD28- CD127- CD25++ CD8+ T cell Absolute Count | Wald ratio | 1 | 2.98E-03 | 1.35 (1.11 -1.65 ) |
|  | CD4 on HLA DR+ CD4+ T cell | Inverse variance weighted | 2 | 2.99E-03 | 1.31 (1.10 -1.57 ) |
|  | CD3 on Effector Memory CD8+ T cell | Inverse variance weighted | 3 | 3.01E-03 | 0.87 (0.80 -0.95 ) |
|  | HLA DR on plasmacytoid Dendritic Cell | Inverse variance weighted | 8 | 3.16E-03 | 1.05 (1.02 -1.09 ) |
|  | CD64 on CD14+ CD16+ monocyte | Inverse variance weighted | 2 | 4.02E-03 | 1.41 (1.12 -1.78 ) |
|  | SSC-A on plasmacytoid Dendritic Cell | Inverse variance weighted | 5 | 4.94E-03 | 0.85 (0.76 -0.95 ) |
|  | SSC-A on CD14+ monocyte | Inverse variance weighted | 5 | 6.65E-03 | 0.91 (0.86 -0.98 ) |
|  | Naive CD4-CD8- T cell %T cell | Wald ratio | 1 | 1.02E-02 | 0.71 (0.55 -0.92 ) |
|  | PDL-1 on CD14- CD16- | Wald ratio | 1 | 1.06E-02 | 0.68 (0.51 -0.92 ) |
|  | CD45RA on naive CD8+ T cell | Inverse variance weighted | 2 | 1.23E-02 | 0.86 (0.76 -0.97 ) |
|  | CD4 regulatory T cell %CD4+ T cell | Inverse variance weighted | 2 | 1.63E-02 | 1.33 (1.05 -1.69 ) |
|  | BAFF-R on CD20- B cell | Wald ratio | 1 | 1.77E-02 | 1.20 (1.03 -1.39 ) |
|  | BAFF-R on IgD+ CD38- unswitched memory B cell | Inverse variance weighted | 5 | 1.80E-02 | 1.05 (1.01 -1.09 ) |
|  | BAFF-R on CD20- CD38- B cell | Wald ratio | 1 | 2.01E-02 | 1.15 (1.02 -1.30 ) |
|  | CD80 on CD62L+ plasmacytoid Dendritic Cell | Inverse variance weighted | 2 | 2.18E-02 | 0.87 (0.77 -0.98 ) |
|  | CD80 on plasmacytoid Dendritic Cell | Inverse variance weighted | 2 | 2.20E-02 | 0.87 (0.77 -0.98 ) |
|  | CD123 on CD62L+ plasmacytoid Dendritic Cell | Inverse variance weighted | 2 | 2.21E-02 | 0.87 (0.78 -0.98 ) |
|  | CD123 on plasmacytoid Dendritic Cell | Inverse variance weighted | 2 | 2.23E-02 | 0.88 (0.78 -0.98 ) |
|  | CD28 on CD39+ CD8+ T cell | Inverse variance weighted | 2 | 2.30E-02 | 1.06 (1.01 -1.11 ) |
|  | Central Memory CD8+ T cell Absolute Count | Wald ratio | 1 | 2.67E-02 | 1.28 (1.03 -1.60 ) |
|  | CD3 on CD39+ activated CD4 regulatory T cell | Inverse variance weighted | 4 | 2.77E-02 | 0.94 (0.89 -0.99 ) |
|  | CD28+ CD45RA+ CD8dim T cell Absolute Count | Inverse variance weighted | 8 | 2.82E-02 | 1.01 (1.00 -1.02 ) |
|  | BAFF-R on IgD+ CD24+ B cell | Inverse variance weighted | 6 | 3.20E-02 | 1.04 (1.00 -1.08 ) |
|  | BAFF-R on memory B cell | Inverse variance weighted | 7 | 3.40E-02 | 1.04 (1.00 -1.08 ) |
|  | BAFF-R on unswitched memory B cell | Inverse variance weighted | 7 | 3.41E-02 | 1.04 (1.00 -1.08 ) |
|  | CD28 on CD39+ activated CD4 regulatory T cell | Inverse variance weighted | 2 | 3.55E-02 | 1.16 (1.01 -1.32 ) |
|  | CD14 on Monocytic Myeloid-Derived Suppressor Cells | Wald ratio | 1 | 3.67E-02 | 0.88 (0.77 -0.99 ) |
|  | BAFF-R on IgD- CD38- B cell | Inverse variance weighted | 7 | 3.72E-02 | 1.04 (1.00 -1.08 ) |
|  | IgD- CD38dim B cell %lymphocyte | Inverse variance weighted | 2 | 3.99E-02 | 1.23 (1.01 -1.49 ) |
|  | BAFF-R on CD24+ CD27+ B cell | Inverse variance weighted | 8 | 3.99E-02 | 1.04 (1.00 -1.08 ) |
|  | CD27 on CD24+ CD27+ B cell | Inverse variance weighted | 9 | 4.25E-02 | 0.95 (0.90 -1.00 ) |
|  | CD25 on CD39+ secreting CD4 regulatory T cell | Inverse variance weighted | 2 | 4.45E-02 | 0.88 (0.78 -1.00 ) |
|  | CD25 on secreting CD4 regulatory T cell | Inverse variance weighted | 2 | 4.45E-02 | 0.88 (0.78 -1.00 ) |
|  | CD39+ secreting CD4 regulatory T cell %secreting CD4 regulatory T cell | Inverse variance weighted | 4 | 4.80E-02 | 1.03 (1.00 -1.07 ) |
|  | CD27 on switched memory B cell | Inverse variance weighted | 9 | 4.92E-02 | 0.95 (0.91 -1.00 ) |
|  | CD28 on CD39+ secreting CD4 regulatory T cell | Inverse variance weighted | 2 | 5.30E-02 | 1.09 (1.00 -1.18 ) |
|  | HLA DR on CD33+ HLA DR+ CD14- | Inverse variance weighted | 2 | 5.47E-02 | 1.06 (1.00 -1.12 ) |
|  | Monocytic Myeloid-Derived Suppressor Cells Absolute Count | Inverse variance weighted | 5 | 6.06E-02 | 0.95 (0.91 -1.00 ) |
|  | CD25 on memory B cell | Inverse variance weighted | 4 | 6.16E-02 | 1.08 (1.00 -1.17 ) |
|  | CD25 on CD24+ CD27+ B cell | Inverse variance weighted | 4 | 6.18E-02 | 1.08 (1.00 -1.17 ) |
|  | CD45 on B cell | Inverse variance weighted | 2 | 6.19E-02 | 1.20 (0.99 -1.46 ) |
|  | CD25 on unswitched memory B cell | Inverse variance weighted | 4 | 6.26E-02 | 1.08 (1.00 -1.18 ) |
|  | CD24 on memory B cell | Inverse variance weighted | 2 | 6.58E-02 | 0.90 (0.80 -1.01 ) |
|  | CD24 on switched memory B cell | Inverse variance weighted | 2 | 6.66E-02 | 0.89 (0.79 -1.01 ) |
|  | CD40 on CD14+ CD16+ monocyte | Inverse variance weighted | 3 | 6.71E-02 | 1.06 (1.00 -1.14 ) |
|  | CD3 on activated & secreting CD4 regulatory T cell | Inverse variance weighted | 4 | 7.01E-02 | 0.95 (0.90 -1.00 ) |
|  | CD3 on activated CD4 regulatory T cell | Inverse variance weighted | 4 | 7.04E-02 | 0.95 (0.90 -1.00 ) |
|  | BAFF-R on switched memory B cell | Inverse variance weighted | 9 | 7.11E-02 | 1.03 (1.00 -1.07 ) |
|  | CD25 on IgD+ CD24+ B cell | Inverse variance weighted | 3 | 7.27E-02 | 1.09 (0.99 -1.20 ) |
|  | BAFF-R on IgD- CD24- B cell | Inverse variance weighted | 8 | 7.39E-02 | 1.04 (1.00 -1.08 ) |
|  | CD39+ secreting CD4 regulatory T cell Absolute Count | Inverse variance weighted | 4 | 7.45E-02 | 1.03 (1.00 -1.07 ) |
|  | BAFF-R on IgD- CD27- B cell | Inverse variance weighted | 8 | 7.56E-02 | 1.04 (1.00 -1.08 ) |
|  | B cell %CD3- lymphocyte | Inverse variance weighted | 3 | 7.58E-02 | 1.07 (0.99 -1.15 ) |
|  | CD39+ CD8+ T cell %T cell | Inverse variance weighted | 8 | 7.71E-02 | 1.03 (1.00 -1.06 ) |
|  | CD19 on IgD- CD38- B cell | Wald ratio | 1 | 7.80E-02 | 0.80 (0.62 -1.03 ) |
|  | CD62L- plasmacytoid Dendritic Cell Absolute Count | Wald ratio | 1 | 7.91E-02 | 0.88 (0.76 -1.02 ) |
|  | CD28 on CD45RA+ CD4+ T cell | Inverse variance weighted | 2 | 7.95E-02 | 1.09 (0.99 -1.20 ) |
|  | CD25 on activated & secreting CD4 regulatory T cell | Wald ratio | 1 | 8.36E-02 | 0.87 (0.74 -1.02 ) |
|  | CD3 on CD39+ secreting CD4 regulatory T cell | Inverse variance weighted | 2 | 8.52E-02 | 0.94 (0.88 -1.01 ) |
|  | CD28- CD8+ T cell Absolute Count | Inverse variance weighted | 3 | 8.60E-02 | 1.21 (0.97 -1.50 ) |
|  | CD28 on CD28+ CD4+ T cell | Inverse variance weighted | 2 | 8.77E-02 | 1.09 (0.99 -1.21 ) |
|  | CD28 on CD4+ T cell | Inverse variance weighted | 2 | 8.88E-02 | 1.10 (0.99 -1.23 ) |
|  | CD27 on IgD- CD38dim B cell | Inverse variance weighted | 6 | 8.90E-02 | 0.96 (0.91 -1.01 ) |
|  | CD3 on CD39+ CD4+ T cell | Inverse variance weighted | 4 | 9.13E-02 | 0.95 (0.90 -1.01 ) |
|  | CD25 on CD39+ CD4+ T cell | Inverse variance weighted | 5 | 9.36E-02 | 0.96 (0.92 -1.01 ) |
|  | HLA DR+ Natural Killer %Natural Killer | Inverse variance weighted | 5 | 9.52E-02 | 1.06 (0.99 -1.13 ) |
|  | SSC-A on lymphocyte | Wald ratio | 1 | 1.01E-01 | 0.84 (0.68 -1.04 ) |
|  | Switched memory B cell %lymphocyte | Inverse variance weighted | 3 | 1.02E-01 | 1.13 (0.98 -1.31 ) |
|  | CD28 on CD39+ CD4+ T cell | Inverse variance weighted | 5 | 1.03E-01 | 1.05 (0.99 -1.11 ) |
|  | CD40 on CD14+ CD16- monocyte | Inverse variance weighted | 3 | 1.04E-01 | 1.06 (0.99 -1.14 ) |
|  | CD28 on CD28+ CD45RA+ CD8+ T cell | Inverse variance weighted | 3 | 1.05E-01 | 1.08 (0.98 -1.18 ) |
|  | CD39+ CD8+ T cell Absolute Count | Inverse variance weighted | 7 | 1.06E-01 | 1.03 (0.99 -1.06 ) |
|  | CD19 on B cell | Wald ratio | 1 | 1.06E-01 | 0.83 (0.66 -1.04 ) |
|  | IgD- CD38- B cell Absolute Count | Wald ratio | 1 | 1.13E-01 | 0.86 (0.72 -1.04 ) |
|  | HLA DR on CD33- HLA DR+ | Inverse variance weighted | 2 | 1.14E-01 | 1.06 (0.99 -1.15 ) |
|  | Activated CD4 regulatory T cell Absolute Count | Inverse variance weighted | 2 | 1.15E-01 | 1.11 (0.98 -1.26 ) |
|  | CD16 on CD14- CD16+ monocyte | Inverse variance weighted | 7 | 1.18E-01 | 1.03 (0.99 -1.08 ) |
|  | HLA DR on CD33dim HLA DR+ CD11b- | Inverse variance weighted | 5 | 1.21E-01 | 1.09 (0.98 -1.22 ) |
|  | CD62L- myeloid Dendritic Cell %Dendritic Cell | Inverse variance weighted | 4 | 1.21E-01 | 0.95 (0.89 -1.01 ) |
|  | CD25++ CD45RA+ CD4 not regulatory T cell Absolute Count | Inverse variance weighted | 3 | 1.21E-01 | 0.93 (0.86 -1.02 ) |
|  | HLA DR+ Natural Killer Absolute Count | Inverse variance weighted | 3 | 1.25E-01 | 1.06 (0.98 -1.14 ) |
|  | Natural Killer %CD3- lymphocyte | Inverse variance weighted | 5 | 1.28E-01 | 0.95 (0.88 -1.02 ) |
|  | CD62L on CD62L+ plasmacytoid Dendritic Cell | Inverse variance weighted | 3 | 1.29E-01 | 0.93 (0.84 -1.02 ) |
|  | CCR7 on naive CD4+ T cell | Inverse variance weighted | 4 | 1.30E-01 | 1.07 (0.98 -1.17 ) |
|  | CD3 on HLA DR+ T cell | Inverse variance weighted | 2 | 1.32E-01 | 0.93 (0.85 -1.02 ) |
|  | CD8+ Natural Killer T Absolute Count | Inverse variance weighted | 2 | 1.33E-01 | 1.51 (0.88 -2.58 ) |
|  | Naive CD8+ T cell %CD8+ T cell | Wald ratio | 1 | 1.34E-01 | 0.77 (0.55 -1.08 ) |
|  | CD62L- Dendritic Cell Absolute Count | Inverse variance weighted | 3 | 1.34E-01 | 0.95 (0.88 -1.02 ) |
|  | CD62L- CD86+ myeloid Dendritic Cell Absolute Count | Inverse variance weighted | 3 | 1.36E-01 | 0.96 (0.90 -1.01 ) |
|  | CD86+ myeloid Dendritic Cell Absolute Count | Inverse variance weighted | 3 | 1.37E-01 | 0.95 (0.89 -1.02 ) |
|  | CD86+ myeloid Dendritic Cell %Dendritic Cell | Inverse variance weighted | 3 | 1.37E-01 | 0.95 (0.89 -1.02 ) |
|  | CD27 on Plasma Blast-Plasma Cell | Wald ratio | 1 | 1.39E-01 | 0.84 (0.66 -1.06 ) |
|  | BAFF-R on IgD- CD38dim B cell | Wald ratio | 1 | 1.39E-01 | 0.86 (0.70 -1.05 ) |
|  | CD16 on CD14+ CD16+ monocyte | Inverse variance weighted | 4 | 1.41E-01 | 1.05 (0.98 -1.11 ) |
|  | CD33dim HLA DR+ CD11b+ Absolute Count | Wald ratio | 1 | 1.41E-01 | 1.11 (0.97 -1.27 ) |
|  | CD27 on T cell | Wald ratio | 1 | 1.41E-01 | 0.92 (0.82 -1.03 ) |
|  | PDL-1 on CD14+ CD16+ monocyte | Wald ratio | 1 | 1.41E-01 | 1.20 (0.94 -1.53 ) |
|  | CX3CR1 on CD14- CD16+ monocyte | Wald ratio | 1 | 1.41E-01 | 0.90 (0.77 -1.04 ) |
|  | CD14 on CD33dim HLA DR+ CD11b+ | Wald ratio | 1 | 1.41E-01 | 0.92 (0.83 -1.03 ) |
|  | CD11c on monocyte | Wald ratio | 1 | 1.41E-01 | 1.10 (0.97 -1.25 ) |
|  | CD11b on CD14+ monocyte | Wald ratio | 1 | 1.41E-01 | 1.04 (0.99 -1.10 ) |
|  | CD11b on Monocytic Myeloid-Derived Suppressor Cells | Wald ratio | 1 | 1.41E-01 | 1.06 (0.98 -1.14 ) |
|  | CD11b on CD33+ HLA DR+ CD14dim | Wald ratio | 1 | 1.41E-01 | 1.05 (0.98 -1.13 ) |
|  | CD34 on Hematopoietic Stem Cell | Inverse variance weighted | 4 | 1.46E-01 | 1.07 (0.98 -1.16 ) |
|  | Plasma Blast-Plasma Cell %lymphocyte | Wald ratio | 1 | 1.46E-01 | 0.82 (0.62 -1.07 ) |
|  | CX3CR1 on CD14+ CD16+ monocyte | Inverse variance weighted | 3 | 1.48E-01 | 0.95 (0.89 -1.02 ) |
|  | Memory B cell %B cell | Inverse variance weighted | 2 | 1.50E-01 | 0.85 (0.69 -1.06 ) |
|  | CD4 on monocyte | Wald ratio | 1 | 1.51E-01 | 1.12 (0.96 -1.31 ) |
|  | SSC-A on monocyte | Inverse variance weighted | 7 | 1.52E-01 | 0.95 (0.90 -1.02 ) |
|  | CD28+ CD45RA+ CD8+ T cell Absolute Count | Inverse variance weighted | 3 | 1.54E-01 | 1.34 (0.90 -2.00 ) |
|  | CD28+ CD45RA+ CD8+ T cell %T cell | Inverse variance weighted | 37 | 1.54E-01 | 1.00 (0.99 -1.00 ) |
|  | CD80 on monocyte | Inverse variance weighted | 6 | 1.56E-01 | 0.96 (0.91 -1.02 ) |
|  | CD25 on IgD+ CD38- unswitched memory B cell | Wald ratio | 1 | 1.58E-01 | 1.07 (0.97 -1.18 ) |
|  | CD11c on myeloid Dendritic Cell | Inverse variance weighted | 5 | 1.59E-01 | 0.93 (0.84 -1.03 ) |
|  | HLA DR on B cell | Inverse variance weighted | 9 | 1.61E-01 | 1.03 (0.99 -1.07 ) |
|  | HLA DR++ monocyte Absolute Count | Wald ratio | 1 | 1.62E-01 | 1.12 (0.96 -1.30 ) |
|  | CD62L on CD62L+ myeloid Dendritic Cell | Wald ratio | 1 | 1.63E-01 | 1.15 (0.95 -1.39 ) |
|  | CD33 on CD66b++ myeloid cell | Inverse variance weighted | 4 | 1.63E-01 | 0.98 (0.94 -1.01 ) |
|  | CD39+ secreting CD4 regulatory T cell %CD4 regulatory T cell | Inverse variance weighted | 6 | 1.65E-01 | 1.02 (0.99 -1.06 ) |
|  | CD62L- CD86+ myeloid Dendritic Cell %Dendritic Cell | Inverse variance weighted | 4 | 1.67E-01 | 0.96 (0.91 -1.02 ) |
|  | CD39+ CD4+ T cell %T cell | Inverse variance weighted | 6 | 1.67E-01 | 1.02 (0.99 -1.06 ) |
|  | CD39 on CD39+ CD8+ T cell | Inverse variance weighted | 2 | 1.67E-01 | 0.91 (0.79 -1.04 ) |
|  | CD39+ CD4+ T cell %CD4+ T cell | Inverse variance weighted | 6 | 1.67E-01 | 1.02 (0.99 -1.05 ) |
|  | CD39+ activated CD4 regulatory T cell %CD4 regulatory T cell | Inverse variance weighted | 6 | 1.70E-01 | 1.02 (0.99 -1.06 ) |
|  | CD45RA on naive CD4+ T cell | Inverse variance weighted | 10 | 1.71E-01 | 0.97 (0.94 -1.01 ) |
|  | CD62L- myeloid Dendritic Cell Absolute Count | Inverse variance weighted | 5 | 1.71E-01 | 0.96 (0.90 -1.02 ) |
|  | CD4 on CD39+ CD4+ T cell | Inverse variance weighted | 5 | 1.74E-01 | 1.04 (0.98 -1.11 ) |
|  | CD25++ CD8+ T cell %CD8+ T cell | Wald ratio | 1 | 1.77E-01 | 1.08 (0.97 -1.20 ) |
|  | CD28 on secreting CD4 regulatory T cell | Inverse variance weighted | 2 | 1.77E-01 | 1.13 (0.95 -1.34 ) |
|  | CD24 on IgD- CD38- B cell | Inverse variance weighted | 2 | 1.80E-01 | 0.93 (0.83 -1.04 ) |
|  | CD33- HLA DR+ Absolute Count | Wald ratio | 1 | 1.80E-01 | 0.90 (0.77 -1.05 ) |
|  | HLA DR+ Natural Killer %CD3- lymphocyte | Inverse variance weighted | 2 | 1.80E-01 | 1.05 (0.98 -1.14 ) |
|  | Myeloid Dendritic Cell Absolute Count | Inverse variance weighted | 2 | 1.81E-01 | 0.95 (0.87 -1.03 ) |
|  | Myeloid Dendritic Cell %Dendritic Cell | Inverse variance weighted | 2 | 1.82E-01 | 0.94 (0.86 -1.03 ) |
|  | CD33 on CD33dim HLA DR+ CD11b+ | Inverse variance weighted | 6 | 1.83E-01 | 0.98 (0.95 -1.01 ) |
|  | CD3 on HLA DR+ CD4+ T cell | Inverse variance weighted | 2 | 1.84E-01 | 0.94 (0.86 -1.03 ) |
|  | CD3 on Effector Memory CD4+ T cell | Inverse variance weighted | 2 | 1.84E-01 | 0.95 (0.88 -1.03 ) |
|  | CX3CR1 on monocyte | Inverse variance weighted | 3 | 1.84E-01 | 0.95 (0.89 -1.02 ) |
|  | CD3 on Terminally Differentiated CD4+ T cell | Wald ratio | 1 | 1.84E-01 | 0.91 (0.78 -1.05 ) |
|  | CD3 on Central Memory CD8+ T cell | Wald ratio | 1 | 1.84E-01 | 0.94 (0.85 -1.03 ) |
|  | CD3 on CD8+ T cell | Wald ratio | 1 | 1.84E-01 | 0.94 (0.85 -1.03 ) |
|  | CD3 on CD39+ CD8+ T cell | Wald ratio | 1 | 1.84E-01 | 0.93 (0.83 -1.04 ) |
|  | CD40 on CD14- CD16+ monocyte | Inverse variance weighted | 7 | 1.86E-01 | 1.04 (0.98 -1.10 ) |
|  | CD16-CD56 on HLA DR+ Natural Killer | Inverse variance weighted | 2 | 1.88E-01 | 0.87 (0.70 -1.07 ) |
|  | IgD- CD38+ B cell %B cell | Wald ratio | 1 | 1.88E-01 | 0.84 (0.66 -1.09 ) |
|  | CD3 on HLA DR+ CD8+ T cell | Wald ratio | 1 | 1.89E-01 | 0.91 (0.79 -1.05 ) |
|  | CX3CR1 on CD14+ CD16- monocyte | Inverse variance weighted | 3 | 1.91E-01 | 0.96 (0.90 -1.02 ) |
|  | Terminally Differentiated CD4-CD8- T cell %CD4-CD8- T cell | Inverse variance weighted | 2 | 1.93E-01 | 0.88 (0.72 -1.07 ) |
|  | IgD+ CD38- B cell %lymphocyte | Inverse variance weighted | 2 | 1.93E-01 | 1.31 (0.87 -1.96 ) |
|  | CD33 on Granulocytic Myeloid-Derived Suppressor Cells | Inverse variance weighted | 3 | 1.97E-01 | 0.97 (0.93 -1.01 ) |
|  | CD4+ CD8dim T cell %lymphocyte | Inverse variance weighted | 2 | 1.97E-01 | 0.92 (0.81 -1.05 ) |
|  | CD3 on CD39+ resting CD4 regulatory T cell | Wald ratio | 1 | 1.98E-01 | 0.92 (0.82 -1.04 ) |
|  | CD8 on CD39+ CD8+ T cell | Inverse variance weighted | 5 | 1.98E-01 | 1.11 (0.95 -1.29 ) |
|  | CD24 on IgD+ CD38- B cell | Inverse variance weighted | 2 | 1.98E-01 | 0.91 (0.79 -1.05 ) |
|  | Effector Memory CD4+ T cell Absolute Count | Wald ratio | 1 | 2.00E-01 | 1.09 (0.96 -1.23 ) |
|  | CD11c+ HLA DR++ monocyte %monocyte | Wald ratio | 1 | 2.01E-01 | 0.86 (0.69 -1.08 ) |
|  | CD45 on CD33- HLA DR- | Inverse variance weighted | 2 | 2.01E-01 | 1.08 (0.96 -1.20 ) |
|  | CD39 on monocyte | Inverse variance weighted | 2 | 2.03E-01 | 0.87 (0.70 -1.08 ) |
|  | CD45RA on resting CD4 regulatory T cell | Inverse variance weighted | 6 | 2.06E-01 | 0.97 (0.92 -1.02 ) |
|  | BAFF-R on transitional B cell | Inverse variance weighted | 8 | 2.08E-01 | 1.03 (0.98 -1.08 ) |
|  | CD4 on naive CD4+ T cell | Inverse variance weighted | 5 | 2.08E-01 | 1.11 (0.94 -1.31 ) |
|  | CD86+ plasmacytoid Dendritic Cell %Dendritic Cell | Wald ratio | 1 | 2.11E-01 | 1.14 (0.93 -1.41 ) |
|  | CCR2 on CD14+ CD16- monocyte | Inverse variance weighted | 2 | 2.13E-01 | 1.09 (0.95 -1.25 ) |
|  | CD86 on myeloid Dendritic Cell | Wald ratio | 1 | 2.18E-01 | 0.93 (0.84 -1.04 ) |
|  | CD4 regulatory T cell %T cell | Wald ratio | 1 | 2.20E-01 | 1.23 (0.88 -1.71 ) |
|  | CCR2 on CD14- CD16- | Inverse variance weighted | 3 | 2.21E-01 | 0.87 (0.69 -1.09 ) |
|  | CD4+ CD8dim T cell %leukocyte | Inverse variance weighted | 2 | 2.24E-01 | 0.92 (0.80 -1.05 ) |
|  | BAFF-R on IgD+ CD38- B cell | Inverse variance weighted | 9 | 2.25E-01 | 1.03 (0.98 -1.07 ) |
|  | CCR7 on naive CD8+ T cell | Inverse variance weighted | 2 | 2.25E-01 | 1.10 (0.95 -1.27 ) |
|  | CD33+ HLA DR+ Absolute Count | Wald ratio | 1 | 2.30E-01 | 0.94 (0.85 -1.04 ) |
|  | CD33+ HLA DR+ CD14- Absolute Count | Wald ratio | 1 | 2.30E-01 | 0.94 (0.85 -1.04 ) |
|  | CD33+ HLA DR+ CD14- %CD33+ HLA DR+ | Wald ratio | 1 | 2.34E-01 | 0.93 (0.83 -1.05 ) |
|  | CCR2 on monocyte | Inverse variance weighted | 2 | 2.35E-01 | 1.05 (0.97 -1.15 ) |
|  | HLA DR on CD33+ HLA DR+ CD14dim | Inverse variance weighted | 3 | 2.37E-01 | 1.05 (0.97 -1.13 ) |
|  | Resting CD4 regulatory T cell %CD4+ T cell | Inverse variance weighted | 5 | 2.42E-01 | 0.97 (0.93 -1.02 ) |
|  | CD4+CD8+ T cell %leukocyte | Wald ratio | 1 | 2.43E-01 | 0.93 (0.82 -1.05 ) |
|  | CD28+ CD45RA- CD8dim T cell Absolute Count | Inverse variance weighted | 3 | 2.44E-01 | 1.03 (0.98 -1.08 ) |
|  | CD28 on resting CD4 regulatory T cell | Wald ratio | 1 | 2.44E-01 | 1.06 (0.96 -1.18 ) |
|  | FSC-A on CD8+ T cell | Wald ratio | 1 | 2.47E-01 | 1.31 (0.83 -2.07 ) |
|  | CD3 on resting CD4 regulatory T cell | Inverse variance weighted | 4 | 2.49E-01 | 1.04 (0.97 -1.11 ) |
|  | CD45RA- CD4+ T cell Absolute Count | Inverse variance weighted | 2 | 2.51E-01 | 1.17 (0.90 -1.52 ) |
|  | BAFF-R on IgD+ CD38- naive B cell | Inverse variance weighted | 8 | 2.52E-01 | 1.02 (0.98 -1.07 ) |
|  | CD25++ CD45RA+ CD4 not regulatory T cell %CD4+ T cell | Inverse variance weighted | 4 | 2.52E-01 | 0.97 (0.91 -1.02 ) |
|  | CD28- CD8dim T cell %T cell | Inverse variance weighted | 3 | 2.53E-01 | 0.93 (0.82 -1.05 ) |
|  | Dendritic Cell Absolute Count | Inverse variance weighted | 2 | 2.53E-01 | 0.95 (0.86 -1.04 ) |
|  | HLA DR on HLA DR+ Natural Killer | Inverse variance weighted | 5 | 2.53E-01 | 1.07 (0.95 -1.20 ) |
|  | CD3 on CD28+ CD45RA- CD8+ T cell | Inverse variance weighted | 4 | 2.54E-01 | 0.95 (0.87 -1.04 ) |
|  | CD8+ and CD8dim T cell %leukocyte | Inverse variance weighted | 2 | 2.55E-01 | 1.13 (0.91 -1.40 ) |
|  | CD25++ CD45RA+ CD4 not regulatory T cell %T cell | Inverse variance weighted | 4 | 2.55E-01 | 0.96 (0.91 -1.03 ) |
|  | CD39 on CD39+ activated CD4 regulatory T cell | Inverse variance weighted | 5 | 2.55E-01 | 1.02 (0.99 -1.05 ) |
|  | CD20 on naive-mature B cell | Inverse variance weighted | 6 | 2.56E-01 | 1.05 (0.96 -1.15 ) |
|  | CD25++ CD8+ T cell Absolute Count | Inverse variance weighted | 3 | 2.57E-01 | 1.06 (0.96 -1.16 ) |
|  | CD45RA+ CD28- CD8+ T cell %CD8+ T cell | Wald ratio | 1 | 2.59E-01 | 0.99 (0.98 -1.01 ) |
|  | IgD+ CD38- B cell Absolute Count | Wald ratio | 1 | 2.59E-01 | 1.45 (0.76 -2.74 ) |
|  | CD27 on IgD- CD38- B cell | Inverse variance weighted | 7 | 2.60E-01 | 0.96 (0.90 -1.03 ) |
|  | Terminally Differentiated CD4+ T cell Absolute Count | Inverse variance weighted | 2 | 2.62E-01 | 0.82 (0.58 -1.16 ) |
|  | CD33 on CD14+ monocyte | Inverse variance weighted | 5 | 2.65E-01 | 0.98 (0.95 -1.01 ) |
|  | CD11c+ CD62L- monocyte Absolute Count | Wald ratio | 1 | 2.66E-01 | 1.20 (0.87 -1.64 ) |
|  | CD80 on granulocyte | Inverse variance weighted | 3 | 2.66E-01 | 1.08 (0.95 -1.22 ) |
|  | CD25 on IgD- CD38- B cell | Inverse variance weighted | 3 | 2.67E-01 | 1.06 (0.95 -1.19 ) |
|  | CD28+ CD45RA+ CD8dim T cell %T cell | Inverse variance weighted | 6 | 2.70E-01 | 0.97 (0.93 -1.02 ) |
|  | CD4+ CD8dim T cell Absolute Count | Inverse variance weighted | 2 | 2.71E-01 | 0.92 (0.78 -1.07 ) |
|  | PDL-1 on CD14+ CD16- monocyte | Wald ratio | 1 | 2.73E-01 | 0.87 (0.67 -1.12 ) |
|  | CD28 on activated & secreting CD4 regulatory T cell | Wald ratio | 1 | 2.73E-01 | 1.08 (0.94 -1.23 ) |
|  | CD28 on activated CD4 regulatory T cell | Wald ratio | 1 | 2.73E-01 | 1.09 (0.94 -1.26 ) |
|  | FSC-A on HLA DR+ Natural Killer | Inverse variance weighted | 3 | 2.74E-01 | 1.05 (0.96 -1.15 ) |
|  | CD33dim HLA DR+ CD11b- %CD33dim HLA DR+ | Inverse variance weighted | 3 | 2.78E-01 | 0.97 (0.92 -1.02 ) |
|  | CD62L- Dendritic Cell %Dendritic Cell | Inverse variance weighted | 4 | 2.79E-01 | 0.96 (0.90 -1.03 ) |
|  | CD11c on granulocyte | Inverse variance weighted | 2 | 2.80E-01 | 1.05 (0.96 -1.15 ) |
|  | CD39 on CD39+ secreting CD4 regulatory T cell | Inverse variance weighted | 5 | 2.80E-01 | 1.02 (0.98 -1.05 ) |
|  | HLA DR on CD33dim HLA DR+ CD11b+ | Inverse variance weighted | 4 | 2.81E-01 | 1.08 (0.94 -1.24 ) |
|  | CD33dim HLA DR+ CD11b+ %CD33dim HLA DR+ | Inverse variance weighted | 3 | 2.82E-01 | 1.03 (0.98 -1.08 ) |
|  | CD4+CD8+ T cell Absolute Count | Inverse variance weighted | 2 | 2.82E-01 | 0.93 (0.82 -1.06 ) |
|  | CD24 on IgD+ CD38- unswitched memory B cell | Inverse variance weighted | 2 | 2.83E-01 | 0.95 (0.87 -1.04 ) |
|  | CD20 on IgD- CD27- B cell | Inverse variance weighted | 4 | 2.84E-01 | 0.94 (0.83 -1.06 ) |
|  | CD33 on CD33dim HLA DR+ CD11b- | Inverse variance weighted | 7 | 2.86E-01 | 0.98 (0.96 -1.01 ) |
|  | Secreting CD4 regulatory T cell %CD4+ T cell | Inverse variance weighted | 7 | 2.93E-01 | 1.02 (0.98 -1.05 ) |
|  | Activated & secreting CD4 regulatory T cell Absolute Count | Inverse variance weighted | 4 | 2.96E-01 | 1.03 (0.97 -1.09 ) |
|  | CD45RA- CD4+ T cell %T cell | Inverse variance weighted | 2 | 3.05E-01 | 1.06 (0.95 -1.19 ) |
|  | CD24 on unswitched memory B cell | Inverse variance weighted | 2 | 3.05E-01 | 0.94 (0.85 -1.05 ) |
|  | CD8 on Effector Memory CD8+ T cell | Inverse variance weighted | 3 | 3.06E-01 | 0.96 (0.88 -1.04 ) |
|  | CD14+ CD16+ monocyte %monocyte | Inverse variance weighted | 3 | 3.06E-01 | 1.04 (0.97 -1.12 ) |
|  | CD8 on CD28+ CD45RA+ CD8+ T cell | Inverse variance weighted | 8 | 3.06E-01 | 1.04 (0.97 -1.11 ) |
|  | Secreting CD4 regulatory T cell Absolute Count | Inverse variance weighted | 4 | 3.09E-01 | 1.03 (0.97 -1.09 ) |
|  | BAFF-R on IgD- CD38+ B cell | Inverse variance weighted | 3 | 3.10E-01 | 1.08 (0.93 -1.25 ) |
|  | CD14+ CD16+ monocyte Absolute Count | Inverse variance weighted | 3 | 3.11E-01 | 1.04 (0.96 -1.13 ) |
|  | CD33+ HLA DR+ CD14dim %CD33+ HLA DR+ | Wald ratio | 1 | 3.15E-01 | 1.08 (0.93 -1.27 ) |
|  | CD45 on CD14+ monocyte | Wald ratio | 1 | 3.17E-01 | 1.23 (0.82 -1.85 ) |
|  | CD33 on Monocytic Myeloid-Derived Suppressor Cells | Inverse variance weighted | 4 | 3.22E-01 | 0.99 (0.96 -1.01 ) |
|  | CD39 on CD39+ CD4+ T cell | Inverse variance weighted | 4 | 3.23E-01 | 1.02 (0.98 -1.06 ) |
|  | CD24 on IgD+ CD24+ B cell | Inverse variance weighted | 2 | 3.23E-01 | 0.95 (0.86 -1.05 ) |
|  | CD45RA on Terminally Differentiated CD8+ T cell | Wald ratio | 1 | 3.23E-01 | 1.11 (0.90 -1.38 ) |
|  | CD25++ CD8+ T cell %T cell | Inverse variance weighted | 2 | 3.26E-01 | 1.05 (0.95 -1.17 ) |
|  | CD33 on CD33+ HLA DR+ CD14dim | Inverse variance weighted | 4 | 3.27E-01 | 0.99 (0.96 -1.01 ) |
|  | CD28 on CD4 regulatory T cell | Wald ratio | 1 | 3.27E-01 | 1.08 (0.93 -1.25 ) |
|  | CD33 on CD33+ HLA DR+ | Inverse variance weighted | 4 | 3.28E-01 | 0.99 (0.96 -1.01 ) |
|  | BAFF-R on IgD+ CD24- B cell | Inverse variance weighted | 10 | 3.29E-01 | 1.02 (0.98 -1.06 ) |
|  | BAFF-R on B cell | Inverse variance weighted | 10 | 3.29E-01 | 1.02 (0.98 -1.07 ) |
|  | BAFF-R on IgD+ B cell | Inverse variance weighted | 10 | 3.31E-01 | 1.02 (0.98 -1.07 ) |
|  | CD33 on CD33+ HLA DR+ CD14- | Inverse variance weighted | 4 | 3.31E-01 | 0.99 (0.96 -1.01 ) |
|  | CD3 on CD4+ T cell | Inverse variance weighted | 2 | 3.32E-01 | 1.08 (0.93 -1.25 ) |
|  | CCR2 on CD62L+ plasmacytoid Dendritic Cell | Inverse variance weighted | 2 | 3.34E-01 | 0.96 (0.88 -1.05 ) |
|  | CCR2 on plasmacytoid Dendritic Cell | Inverse variance weighted | 2 | 3.34E-01 | 0.96 (0.88 -1.05 ) |
|  | Immature Myeloid-Derived Suppressor Cells Absolute Count | Inverse variance weighted | 2 | 3.36E-01 | 0.96 (0.89 -1.04 ) |
|  | Terminally Differentiated CD8+ T cell %CD8+ T cell | Inverse variance weighted | 3 | 3.36E-01 | 0.95 (0.85 -1.06 ) |
|  | HLA DR+ CD8+ T cell %T cell | Inverse variance weighted | 3 | 3.36E-01 | 0.96 (0.89 -1.04 ) |
|  | BAFF-R on IgD+ CD38+ B cell | Inverse variance weighted | 10 | 3.37E-01 | 1.02 (0.98 -1.07 ) |
|  | CD38 on CD3- CD19- | Wald ratio | 1 | 3.41E-01 | 0.91 (0.76 -1.10 ) |
|  | HLA DR on HLA DR+ T cell | Wald ratio | 1 | 3.44E-01 | 1.08 (0.92 -1.26 ) |
|  | HLA DR on HLA DR+ CD4+ T cell | Wald ratio | 1 | 3.44E-01 | 1.11 (0.89 -1.39 ) |
|  | CD45RA+ CD8+ T cell Absolute Count | Inverse variance weighted | 3 | 3.44E-01 | 1.23 (0.80 -1.87 ) |
|  | SSC-A on HLA DR+ Natural Killer | Inverse variance weighted | 3 | 3.44E-01 | 1.04 (0.96 -1.13 ) |
|  | CD127 on CD8+ T cell | Wald ratio | 1 | 3.46E-01 | 1.10 (0.90 -1.34 ) |
|  | Naive CD8+ T cell %T cell | Inverse variance weighted | 3 | 3.47E-01 | 1.00 (0.98 -1.01 ) |
|  | CD14+ CD16- monocyte %monocyte | Wald ratio | 1 | 3.47E-01 | 0.76 (0.43 -1.34 ) |
|  | CD16+ monocyte %monocyte | Wald ratio | 1 | 3.47E-01 | 1.31 (0.74 -2.31 ) |
|  | CD8 on HLA DR+ CD8+ T cell | Inverse variance weighted | 2 | 3.49E-01 | 1.07 (0.93 -1.25 ) |
|  | BAFF-R on IgD+ CD38dim B cell | Inverse variance weighted | 10 | 3.54E-01 | 1.02 (0.98 -1.06 ) |
|  | CD39+ CD4+ T cell Absolute Count | Inverse variance weighted | 5 | 3.57E-01 | 0.97 (0.90 -1.04 ) |
|  | Memory B cell %lymphocyte | Inverse variance weighted | 2 | 3.58E-01 | 1.11 (0.89 -1.39 ) |
|  | Natural Killer T %T cell | Inverse variance weighted | 5 | 3.60E-01 | 0.95 (0.86 -1.06 ) |
|  | CD45 on granulocyte | Wald ratio | 1 | 3.62E-01 | 0.90 (0.72 -1.13 ) |
|  | CD3 on CD28+ CD45RA+ CD8+ T cell | Inverse variance weighted | 3 | 3.62E-01 | 1.04 (0.96 -1.13 ) |
|  | BAFF-R on naive-mature B cell | Inverse variance weighted | 10 | 3.62E-01 | 1.02 (0.98 -1.06 ) |
|  | CD4+/CD8+ T cell | Wald ratio | 1 | 3.63E-01 | 0.83 (0.57 -1.23 ) |
|  | CD8+ T cell %T cell | Wald ratio | 1 | 3.63E-01 | 1.19 (0.81 -1.75 ) |
|  | HLA DR on CD14- CD16+ monocyte | Inverse variance weighted | 8 | 3.71E-01 | 1.04 (0.95 -1.14 ) |
|  | CD19 on IgD- CD38dim B cell | Inverse variance weighted | 4 | 3.74E-01 | 0.95 (0.86 -1.06 ) |
|  | CD45RA+ CD28- CD8+ T cell Absolute Count | Inverse variance weighted | 417 | 3.77E-01 | 1.00 (1.00 -1.00 ) |
|  | HLA DR on CD14+ CD16+ monocyte | Inverse variance weighted | 4 | 3.78E-01 | 1.03 (0.96 -1.12 ) |
|  | CD39+ resting CD4 regulatory T cell Absolute Count | Inverse variance weighted | 8 | 3.79E-01 | 1.03 (0.97 -1.10 ) |
|  | Granulocytic Myeloid-Derived Suppressor Cells Absolute Count | Inverse variance weighted | 2 | 3.83E-01 | 0.95 (0.84 -1.07 ) |
|  | HLA DR+ CD8+ T cell %lymphocyte | Inverse variance weighted | 2 | 3.84E-01 | 0.96 (0.88 -1.05 ) |
|  | CD20 on IgD+ B cell | Inverse variance weighted | 5 | 3.87E-01 | 1.04 (0.95 -1.14 ) |
|  | IgD+ B cell %Lymphocyte | Inverse variance weighted | 2 | 3.87E-01 | 1.10 (0.88 -1.38 ) |
|  | CD62L on monocyte | Inverse variance weighted | 2 | 3.87E-01 | 1.04 (0.96 -1.13 ) |
|  | PDL-1 on monocyte | Inverse variance weighted | 2 | 3.99E-01 | 1.05 (0.94 -1.17 ) |
|  | CD3 on CD45RA- CD4+ T cell | Inverse variance weighted | 2 | 4.00E-01 | 0.96 (0.88 -1.05 ) |
|  | IgD on IgD+ B cell | Inverse variance weighted | 2 | 4.02E-01 | 1.03 (0.97 -1.09 ) |
|  | CD14- CD16- Absolute Count | Inverse variance weighted | 3 | 4.02E-01 | 1.11 (0.87 -1.44 ) |
|  | CD3 on Naive CD4+ T cell | Inverse variance weighted | 2 | 4.02E-01 | 0.97 (0.90 -1.04 ) |
|  | Naive-mature B cell %B cell | Wald ratio | 1 | 4.02E-01 | 1.13 (0.85 -1.52 ) |
|  | Naive CD4+ T cell %CD4+ T cell | Inverse variance weighted | 4 | 4.03E-01 | 1.10 (0.88 -1.38 ) |
|  | CD20 on B cell | Inverse variance weighted | 4 | 4.09E-01 | 1.10 (0.88 -1.37 ) |
|  | CD28+ CD45RA+ CD8dim T cell %CD8dim T cell | Inverse variance weighted | 5 | 4.14E-01 | 0.98 (0.94 -1.03 ) |
|  | CD45RA- CD4+ T cell %CD4+ T cell | Inverse variance weighted | 4 | 4.15E-01 | 1.02 (0.97 -1.08 ) |
|  | IgD+ CD24+ B cell %lymphocyte | Inverse variance weighted | 2 | 4.19E-01 | 1.09 (0.88 -1.36 ) |
|  | HVEM on T cell | Wald ratio | 1 | 4.21E-01 | 1.05 (0.93 -1.19 ) |
|  | HVEM on Effector Memory CD8+ T cell | Wald ratio | 1 | 4.21E-01 | 1.05 (0.93 -1.19 ) |
|  | HVEM on CD4+ T cell | Wald ratio | 1 | 4.21E-01 | 1.05 (0.93 -1.19 ) |
|  | HVEM on Central Memory CD4+ T cell | Wald ratio | 1 | 4.21E-01 | 1.05 (0.93 -1.19 ) |
|  | HVEM on CD45RA- CD4+ T cell | Wald ratio | 1 | 4.21E-01 | 1.05 (0.93 -1.18 ) |
|  | HVEM on Central Memory CD8+ T cell | Wald ratio | 1 | 4.21E-01 | 1.05 (0.93 -1.17 ) |
|  | CD39+ resting CD4 regulatory T cell %CD4 regulatory T cell | Inverse variance weighted | 11 | 4.22E-01 | 1.02 (0.98 -1.05 ) |
|  | CD25 on CD39+ CD4 regulatory T cell | Wald ratio | 1 | 4.23E-01 | 1.09 (0.89 -1.33 ) |
|  | CD25 on CD39+ activated CD4 regulatory T cell | Wald ratio | 1 | 4.23E-01 | 1.09 (0.88 -1.35 ) |
|  | Basophil Absolute Count | Inverse variance weighted | 3 | 4.23E-01 | 0.99 (0.96 -1.02 ) |
|  | Activated & secreting CD4 regulatory T cell %CD4+ T cell | Inverse variance weighted | 9 | 4.24E-01 | 1.01 (0.98 -1.04 ) |
|  | CD33dim HLA DR- Absolute Count | Inverse variance weighted | 3 | 4.24E-01 | 0.99 (0.95 -1.02 ) |
|  | CD20 on IgD+ CD38dim B cell | Inverse variance weighted | 7 | 4.26E-01 | 1.04 (0.94 -1.16 ) |
|  | Resting CD4 regulatory T cell %CD4 regulatory T cell | Inverse variance weighted | 9 | 4.30E-01 | 0.98 (0.94 -1.03 ) |
|  | CD3 on Central Memory CD4+ T cell | Inverse variance weighted | 2 | 4.34E-01 | 0.97 (0.89 -1.05 ) |
|  | CD25 on IgD- CD38dim B cell | Inverse variance weighted | 3 | 4.35E-01 | 0.96 (0.86 -1.07 ) |
|  | Terminally Differentiated CD8+ T cell %T cell | Inverse variance weighted | 2 | 4.44E-01 | 0.94 (0.81 -1.10 ) |
|  | CD45RA+ CD28- CD8+ T cell %T cell | Inverse variance weighted | 63 | 4.45E-01 | 1.00 (1.00 -1.00 ) |
|  | CD4-CD8- Natural Killer T Absolute Count | Wald ratio | 1 | 4.46E-01 | 1.09 (0.88 -1.34 ) |
|  | CD28+ CD4-CD8- T cell %T cell | Wald ratio | 1 | 4.46E-01 | 1.11 (0.85 -1.46 ) |
|  | Effector Memory CD4+ T cell %T cell | Inverse variance weighted | 4 | 4.46E-01 | 0.92 (0.73 -1.15 ) |
|  | CD4RA on Terminally Differentiated CD4+ T cell | Inverse variance weighted | 3 | 4.47E-01 | 0.97 (0.90 -1.05 ) |
|  | CD4 on CD45RA+ CD4+ T cell | Inverse variance weighted | 2 | 4.47E-01 | 0.96 (0.87 -1.06 ) |
|  | CD19 on IgD+ CD24+ B cell | Inverse variance weighted | 4 | 4.48E-01 | 0.96 (0.86 -1.07 ) |
|  | CCR2 on CD14+ CD16+ monocyte | Wald ratio | 1 | 4.48E-01 | 0.86 (0.59 -1.26 ) |
|  | CD3 on T cell | Inverse variance weighted | 3 | 4.48E-01 | 0.97 (0.89 -1.05 ) |
|  | CD4 on Central Memory CD4+ T cell | Inverse variance weighted | 2 | 4.49E-01 | 0.96 (0.86 -1.07 ) |
|  | SSC-A on T cell | Wald ratio | 1 | 4.50E-01 | 1.16 (0.79 -1.69 ) |
|  | CD28- CD8+ T cell %CD8+ T cell | Inverse variance weighted | 3 | 4.54E-01 | 1.06 (0.91 -1.24 ) |
|  | CD127 on CD45RA- CD4 not regulatory T cell | Wald ratio | 1 | 4.55E-01 | 0.91 (0.72 -1.16 ) |
|  | CD25 on IgD- CD24- B cell | Inverse variance weighted | 2 | 4.55E-01 | 0.92 (0.75 -1.14 ) |
|  | HLA DR+ CD4+ T cell %T cell | Wald ratio | 1 | 4.57E-01 | 0.95 (0.82 -1.09 ) |
|  | CD28 on CD45RA- CD4 not regulatory T cell | Inverse variance weighted | 3 | 4.57E-01 | 1.04 (0.94 -1.15 ) |
|  | CD62L- monocyte %monocyte | Inverse variance weighted | 3 | 4.62E-01 | 0.94 (0.80 -1.10 ) |
|  | CD33 on CD33dim HLA DR- | Inverse variance weighted | 6 | 4.64E-01 | 0.99 (0.95 -1.02 ) |
|  | Transitional B cell Absolute Count | Wald ratio | 1 | 4.66E-01 | 1.11 (0.83 -1.49 ) |
|  | CD45RA- CD28- CD8+ T cell %T cell | Inverse variance weighted | 40 | 4.71E-01 | 1.00 (1.00 -1.00 ) |
|  | CD45RA+ CD8+ T cell %CD8+ T cell | Inverse variance weighted | 4 | 4.71E-01 | 1.01 (0.98 -1.04 ) |
|  | Memory B cell Absolute Count | Wald ratio | 1 | 4.71E-01 | 1.23 (0.70 -2.14 ) |
|  | IgD+ CD24+ B cell Absolute Count | Wald ratio | 1 | 4.71E-01 | 1.20 (0.73 -1.96 ) |
|  | CD24+ CD27+ B cell Absolute Count | Wald ratio | 1 | 4.71E-01 | 1.23 (0.70 -2.18 ) |
|  | CD3 on CD28+ CD4+ T cell | Inverse variance weighted | 2 | 4.72E-01 | 0.96 (0.87 -1.07 ) |
|  | HLA DR++ monocyte %monocyte | Inverse variance weighted | 4 | 4.75E-01 | 1.04 (0.94 -1.14 ) |
|  | T/B cell | Inverse variance weighted | 2 | 4.75E-01 | 0.93 (0.76 -1.14 ) |
|  | CD45RA on CD39+ resting CD4 regulatory T cell | Wald ratio | 1 | 4.76E-01 | 1.03 (0.95 -1.11 ) |
|  | CD3 on CD28- CD8+ T cell | Inverse variance weighted | 2 | 4.79E-01 | 0.94 (0.79 -1.11 ) |
|  | Natural Killer T Absolute Count | Inverse variance weighted | 5 | 4.81E-01 | 0.96 (0.86 -1.07 ) |
|  | FSC-A on Natural Killer | Inverse variance weighted | 3 | 4.81E-01 | 1.04 (0.93 -1.18 ) |
|  | IgD+ CD38- B cell %B cell | Inverse variance weighted | 2 | 4.81E-01 | 0.86 (0.57 -1.31 ) |
|  | CD8dim Natural Killer T %T cell | Inverse variance weighted | 5 | 4.82E-01 | 0.97 (0.88 -1.06 ) |
|  | Basophil %CD33dim HLA DR- CD66b- | Inverse variance weighted | 3 | 4.83E-01 | 0.98 (0.92 -1.04 ) |
|  | IgD- CD27- B cell %lymphocyte | Inverse variance weighted | 2 | 4.84E-01 | 1.04 (0.92 -1.18 ) |
|  | CD20 on transitional B cell | Inverse variance weighted | 2 | 4.86E-01 | 0.96 (0.85 -1.08 ) |
|  | HLA DR++ monocyte %leukocyte | Inverse variance weighted | 2 | 4.88E-01 | 1.06 (0.89 -1.27 ) |
|  | CD20 on CD24+ CD27+ B cell | Inverse variance weighted | 5 | 4.88E-01 | 0.97 (0.87 -1.07 ) |
|  | CD25 on CD45RA+ CD4 not regulatory T cell | Wald ratio | 1 | 4.91E-01 | 1.06 (0.90 -1.24 ) |
|  | CD28+ CD45RA- CD8dim T cell %CD8dim T cell | Inverse variance weighted | 2 | 5.04E-01 | 1.02 (0.97 -1.07 ) |
|  | CD19 on memory B cell | Inverse variance weighted | 3 | 5.08E-01 | 0.95 (0.82 -1.10 ) |
|  | CD19 on CD24+ CD27+ B cell | Inverse variance weighted | 3 | 5.10E-01 | 0.96 (0.84 -1.09 ) |
|  | CD3 on CD4 regulatory T cell | Inverse variance weighted | 2 | 5.12E-01 | 0.97 (0.87 -1.07 ) |
|  | CD19 on transitional B cell | Inverse variance weighted | 4 | 5.16E-01 | 0.97 (0.88 -1.07 ) |
|  | CD25 on IgD+ CD38- B cell | Inverse variance weighted | 3 | 5.17E-01 | 0.96 (0.84 -1.09 ) |
|  | CD25++ CD45RA- CD4 not regulatory T cell Absolute Count | Inverse variance weighted | 2 | 5.18E-01 | 1.07 (0.86 -1.33 ) |
|  | CD45 on HLA DR+ T cell | Wald ratio | 1 | 5.18E-01 | 0.95 (0.83 -1.10 ) |
|  | SSC-A on HLA DR+ T cell | Wald ratio | 1 | 5.18E-01 | 0.94 (0.78 -1.13 ) |
|  | CD20 on memory B cell | Inverse variance weighted | 5 | 5.19E-01 | 0.96 (0.86 -1.08 ) |
|  | CD3 on Terminally Differentiated CD8+ T cell | Inverse variance weighted | 2 | 5.19E-01 | 0.93 (0.74 -1.16 ) |
|  | CD19 on unswitched memory B cell | Inverse variance weighted | 4 | 5.23E-01 | 0.97 (0.87 -1.07 ) |
|  | CD4+ T cell %leukocyte | Wald ratio | 1 | 5.27E-01 | 1.12 (0.79 -1.58 ) |
|  | CD28 on CD39+ resting CD4 regulatory T cell | Inverse variance weighted | 2 | 5.29E-01 | 1.08 (0.84 -1.39 ) |
|  | CD3 on secreting CD4 regulatory T cell | Inverse variance weighted | 2 | 5.30E-01 | 0.97 (0.87 -1.08 ) |
|  | CD4 on CD28+ CD4+ T cell | Inverse variance weighted | 2 | 5.36E-01 | 1.07 (0.87 -1.31 ) |
|  | HLA DR on CD14+ CD16- monocyte | Inverse variance weighted | 5 | 5.38E-01 | 1.02 (0.95 -1.10 ) |
|  | CD39+ activated CD4 regulatory T cell Absolute Count | Inverse variance weighted | 4 | 5.39E-01 | 0.98 (0.91 -1.05 ) |
|  | HLA DR on CD14+ monocyte | Inverse variance weighted | 5 | 5.45E-01 | 1.02 (0.95 -1.11 ) |
|  | CD24 on transitional B cell | Wald ratio | 1 | 5.48E-01 | 0.93 (0.74 -1.18 ) |
|  | Naive CD8+ T cell Absolute Count | Wald ratio | 1 | 5.49E-01 | 0.88 (0.57 -1.35 ) |
|  | CD25 on IgD+ B cell | Inverse variance weighted | 2 | 5.55E-01 | 0.96 (0.85 -1.09 ) |
|  | CD19 on IgD+ CD38dim B cell | Inverse variance weighted | 4 | 5.55E-01 | 0.97 (0.87 -1.08 ) |
|  | CD19 on IgD+ CD38+ B cell | Inverse variance weighted | 4 | 5.56E-01 | 0.97 (0.89 -1.07 ) |
|  | Immature Myeloid-Derived Suppressor Cells %CD33dim HLA DR- CD66b- | Inverse variance weighted | 2 | 5.56E-01 | 1.03 (0.93 -1.15 ) |
|  | Activated CD4 regulatory T cell %CD4 regulatory T cell | Inverse variance weighted | 3 | 5.60E-01 | 1.02 (0.96 -1.08 ) |
|  | CD38 on IgD+ B cell | Wald ratio | 1 | 5.60E-01 | 1.07 (0.84 -1.37 ) |
|  | HLA DR+ CD8+ T cell Absolute Count | Inverse variance weighted | 3 | 5.63E-01 | 0.98 (0.90 -1.06 ) |
|  | CD20 on IgD+ CD24- B cell | Inverse variance weighted | 4 | 5.63E-01 | 1.04 (0.91 -1.18 ) |
|  | PDL-1 on CD14- CD16+ monocyte | Inverse variance weighted | 2 | 5.64E-01 | 1.04 (0.91 -1.19 ) |
|  | IgD+ CD38+ B cell %lymphocyte | Wald ratio | 1 | 5.64E-01 | 0.74 (0.26 -2.07 ) |
|  | CD20 on IgD+ CD24+ B cell | Inverse variance weighted | 2 | 5.68E-01 | 0.96 (0.83 -1.11 ) |
|  | CD40 on monocytes | Inverse variance weighted | 4 | 5.72E-01 | 1.02 (0.95 -1.11 ) |
|  | T cell %lymphocyte | Inverse variance weighted | 2 | 5.78E-01 | 1.04 (0.91 -1.18 ) |
|  | B cell %lymphocyte | Inverse variance weighted | 2 | 5.79E-01 | 1.06 (0.86 -1.31 ) |
|  | Plasma Blast-Plasma Cell %B cell | Wald ratio | 1 | 5.79E-01 | 0.90 (0.62 -1.31 ) |
|  | CD4-CD8- T cell Absolute Count | Inverse variance weighted | 2 | 5.79E-01 | 1.05 (0.89 -1.23 ) |
|  | CD19 on naive-mature B cell | Inverse variance weighted | 2 | 5.83E-01 | 1.10 (0.78 -1.55 ) |
|  | HLA DR+ T cell Absolute Count | Inverse variance weighted | 3 | 5.84E-01 | 0.98 (0.90 -1.06 ) |
|  | CD8 on naive CD8+ T cell | Wald ratio | 1 | 5.85E-01 | 0.97 (0.89 -1.07 ) |
|  | HLA DR+ CD4+ T cell %lymphocyte | Inverse variance weighted | 2 | 5.85E-01 | 0.97 (0.86 -1.09 ) |
|  | Terminally Differentiated CD8+ T cell Absolute Count | Wald ratio | 1 | 5.85E-01 | 1.08 (0.81 -1.44 ) |
|  | CD28+ CD45RA- CD8dim T cell %T cell | Inverse variance weighted | 3 | 5.88E-01 | 1.01 (0.97 -1.06 ) |
|  | IgD+ CD38+ B cell Absolute Count | Inverse variance weighted | 2 | 5.89E-01 | 1.08 (0.81 -1.44 ) |
|  | Terminally Differentiated CD4-CD8- T cell %T cell | Inverse variance weighted | 3 | 5.92E-01 | 0.95 (0.77 -1.16 ) |
|  | CD127 on CD45RA+ CD4+ T cell | Inverse variance weighted | 4 | 5.95E-01 | 1.07 (0.84 -1.35 ) |
|  | IgD on IgD+ CD38- B cell | Wald ratio | 1 | 5.98E-01 | 1.02 (0.95 -1.10 ) |
|  | SSC-A on Natural Killer T | Inverse variance weighted | 2 | 5.99E-01 | 0.94 (0.76 -1.18 ) |
|  | CD8dim Natural Killer T %lymphocyte | Inverse variance weighted | 6 | 6.01E-01 | 0.98 (0.90 -1.07 ) |
|  | CD127 on CD28+ CD4+ T cell | Inverse variance weighted | 3 | 6.12E-01 | 1.04 (0.90 -1.19 ) |
|  | CD20 on IgD- CD24- B cell | Inverse variance weighted | 2 | 6.14E-01 | 1.07 (0.82 -1.39 ) |
|  | CD45 on Natural Killer | Inverse variance weighted | 2 | 6.16E-01 | 1.08 (0.80 -1.46 ) |
|  | SSC-A on Natural Killer | Wald ratio | 1 | 6.16E-01 | 1.04 (0.89 -1.22 ) |
|  | CD14- CD16+ monocyte Absolute Count | Wald ratio | 1 | 6.17E-01 | 1.04 (0.89 -1.22 ) |
|  | CCR2 on CD14- CD16+ monocyte | Wald ratio | 1 | 6.17E-01 | 0.93 (0.71 -1.22 ) |
|  | CD8 on Natural Killer T | Inverse variance weighted | 2 | 6.18E-01 | 1.12 (0.72 -1.75 ) |
|  | SSC-A on granulocyte | Inverse variance weighted | 3 | 6.20E-01 | 0.97 (0.88 -1.08 ) |
|  | CD25 on IgD+ CD38dim B cell | Inverse variance weighted | 2 | 6.22E-01 | 0.96 (0.83 -1.12 ) |
|  | IgD on transitional B cell | Inverse variance weighted | 5 | 6.24E-01 | 1.01 (0.96 -1.07 ) |
|  | CD8 on CD28+ CD45RA- CD8+ T cell | Inverse variance weighted | 2 | 6.25E-01 | 0.95 (0.78 -1.16 ) |
|  | SSC-A on CD4+ T cell | Inverse variance weighted | 4 | 6.30E-01 | 0.93 (0.68 -1.26 ) |
|  | CD25++ CD4+ T cell Absolute Count | Wald ratio | 1 | 6.31E-01 | 0.95 (0.79 -1.16 ) |
|  | CD25++ CD4+ T cell %CD4+ T cell | Wald ratio | 1 | 6.31E-01 | 0.96 (0.83 -1.12 ) |
|  | CD25++ CD4+ T cell %T cell | Wald ratio | 1 | 6.31E-01 | 0.96 (0.81 -1.14 ) |
|  | CD25 on CD4+ T cell | Wald ratio | 1 | 6.31E-01 | 0.94 (0.75 -1.19 ) |
|  | HVEM on Terminally Differentiated CD4+ T cell | Wald ratio | 1 | 6.34E-01 | 1.04 (0.89 -1.20 ) |
|  | HVEM on CD8+ T cell | Wald ratio | 1 | 6.34E-01 | 1.03 (0.90 -1.19 ) |
|  | CD4 on CD39+ secreting CD4 regulatory T cell | Inverse variance weighted | 3 | 6.35E-01 | 1.06 (0.83 -1.35 ) |
|  | CD33 on basophil | Inverse variance weighted | 7 | 6.35E-01 | 0.99 (0.95 -1.03 ) |
|  | CD24+ CD27+ B cell %lymphocyte | Inverse variance weighted | 3 | 6.35E-01 | 1.04 (0.88 -1.24 ) |
|  | CD3 on Natural Killer T | Inverse variance weighted | 2 | 6.36E-01 | 1.05 (0.87 -1.26 ) |
|  | HVEM on naive CD4+ T cell | Wald ratio | 1 | 6.37E-01 | 1.03 (0.90 -1.19 ) |
|  | CD28+ CD45RA- CD8+ T cell Absolute Count | Inverse variance weighted | 2 | 6.41E-01 | 1.02 (0.93 -1.13 ) |
|  | CD20- CD38- B cell %B cell | Wald ratio | 1 | 6.42E-01 | 1.03 (0.90 -1.19 ) |
|  | CD25 on IgD- CD27- B cell | Inverse variance weighted | 2 | 6.47E-01 | 1.04 (0.89 -1.20 ) |
|  | FSC-A on monocyte | Inverse variance weighted | 2 | 6.48E-01 | 0.90 (0.58 -1.41 ) |
|  | CD27 on IgD+ CD24+ B cell | Inverse variance weighted | 6 | 6.49E-01 | 0.98 (0.92 -1.05 ) |
|  | CD45RA+ CD8+ T cell %T cell | Inverse variance weighted | 3 | 6.49E-01 | 1.08 (0.78 -1.50 ) |
|  | CD33 on Immature Myeloid-Derived Suppressor Cells | Inverse variance weighted | 6 | 6.50E-01 | 0.99 (0.94 -1.04 ) |
|  | CD24+ CD27+ B cell %B cell | Inverse variance weighted | 3 | 6.57E-01 | 0.97 (0.87 -1.09 ) |
|  | Natural Killer T %lymphocyte | Inverse variance weighted | 7 | 6.62E-01 | 0.98 (0.89 -1.07 ) |
|  | CD80 on myeloid Dendritic Cell | Inverse variance weighted | 4 | 6.62E-01 | 0.97 (0.85 -1.11 ) |
|  | CD33- HLA DR- Absolute Count | Wald ratio | 1 | 6.64E-01 | 0.96 (0.79 -1.16 ) |
|  | CD4 on CD4+ T cell | Wald ratio | 1 | 6.70E-01 | 0.97 (0.84 -1.12 ) |
|  | CD4 on CD4 regulatory T cell | Wald ratio | 1 | 6.70E-01 | 0.97 (0.82 -1.14 ) |
|  | CD4 on resting CD4 regulatory T cell | Wald ratio | 1 | 6.70E-01 | 0.96 (0.77 -1.18 ) |
|  | CD4 on activated CD4 regulatory T cell | Wald ratio | 1 | 6.70E-01 | 0.97 (0.83 -1.13 ) |
|  | CD4 on secreting CD4 regulatory T cell | Wald ratio | 1 | 6.70E-01 | 0.96 (0.79 -1.16 ) |
|  | CD4 on activated & secreting CD4 regulatory T cell | Wald ratio | 1 | 6.70E-01 | 0.96 (0.82 -1.14 ) |
|  | CD3- lymphocyte %leukocyte | Wald ratio | 1 | 6.70E-01 | 1.06 (0.81 -1.40 ) |
|  | CD28- CD4-CD8- T cell %CD4-CD8- T cell | Wald ratio | 1 | 6.70E-01 | 0.95 (0.74 -1.22 ) |
|  | CD28+ CD4-CD8- T cell %CD4-CD8- T cell | Wald ratio | 1 | 6.70E-01 | 1.06 (0.82 -1.35 ) |
|  | CD28- CD8dim T cell %CD8dim T cell | Wald ratio | 1 | 6.70E-01 | 0.94 (0.72 -1.23 ) |
|  | CD28- CD4-CD8- T cell Absolute Count | Inverse variance weighted | 2 | 6.72E-01 | 1.03 (0.89 -1.20 ) |
|  | FSC-A on plasmacytoid Dendritic Cell | Inverse variance weighted | 4 | 6.73E-01 | 1.03 (0.90 -1.18 ) |
|  | CD62L- plasmacytoid Dendritic Cell %Dendritic Cell | Inverse variance weighted | 2 | 6.81E-01 | 0.96 (0.78 -1.18 ) |
|  | CD19 on IgD+ B cell | Inverse variance weighted | 2 | 6.82E-01 | 1.09 (0.72 -1.67 ) |
|  | CD86 on monocyte | Wald ratio | 1 | 6.86E-01 | 0.95 (0.75 -1.21 ) |
|  | Activated & resting CD4 regulatory T cell %CD4 regulatory T cell | Inverse variance weighted | 5 | 6.90E-01 | 0.99 (0.93 -1.05 ) |
|  | Secreting CD4 regulatory T cell %CD4 regulatory T cell | Inverse variance weighted | 5 | 6.91E-01 | 1.01 (0.96 -1.07 ) |
|  | CD39 on granulocyte | Inverse variance weighted | 2 | 6.92E-01 | 0.96 (0.78 -1.18 ) |
|  | CD27 on IgD- CD38+ B cell | Inverse variance weighted | 2 | 6.93E-01 | 1.06 (0.80 -1.39 ) |
|  | CD20 on IgD+ CD38+ B cell | Inverse variance weighted | 5 | 6.94E-01 | 1.02 (0.93 -1.11 ) |
|  | CD25 on IgD+ CD38+ B cell | Inverse variance weighted | 3 | 6.95E-01 | 1.02 (0.91 -1.15 ) |
|  | Terminally Differentiated CD4+ T cell %T cell | Inverse variance weighted | 5 | 6.96E-01 | 0.97 (0.82 -1.15 ) |
|  | Activated & resting CD4 regulatory T cell Absolute Count | Wald ratio | 1 | 7.00E-01 | 0.95 (0.76 -1.21 ) |
|  | Central Memory CD4+ T cell %CD4+ T cell | Wald ratio | 1 | 7.00E-01 | 1.04 (0.85 -1.28 ) |
|  | IgD+ B cell %B cell | Wald ratio | 1 | 7.01E-01 | 0.95 (0.74 -1.23 ) |
|  | CD14+ CD16- monocyte Absolute Count | Inverse variance weighted | 3 | 7.02E-01 | 0.97 (0.83 -1.13 ) |
|  | CD127 on CD28+ CD45RA- CD8+ T cell | Wald ratio | 1 | 7.03E-01 | 1.02 (0.93 -1.11 ) |
|  | CD45 on HLA DR+ CD4+ T cell | Inverse variance weighted | 2 | 7.05E-01 | 0.97 (0.82 -1.14 ) |
|  | CD39+ resting CD4 regulatory T cell %resting CD4 regulatory T cell | Inverse variance weighted | 5 | 7.05E-01 | 0.99 (0.91 -1.06 ) |
|  | HVEM on Effector Memory CD4+ T cell | Wald ratio | 1 | 7.07E-01 | 1.02 (0.91 -1.16 ) |
|  | HLA DR+ CD4+ T cell Absolute Count | Inverse variance weighted | 2 | 7.11E-01 | 0.98 (0.86 -1.11 ) |
|  | CD45 on CD33+ HLA DR+ CD14- | Wald ratio | 1 | 7.12E-01 | 0.97 (0.80 -1.16 ) |
|  | Plasmacytoid Dendritic Cell %Dendritic Cell | Inverse variance weighted | 2 | 7.14E-01 | 1.03 (0.88 -1.20 ) |
|  | HLA DR on monocyte | Inverse variance weighted | 4 | 7.17E-01 | 1.02 (0.93 -1.11 ) |
|  | Naive CD4+ T cell Absolute Count | Inverse variance weighted | 2 | 7.17E-01 | 1.10 (0.65 -1.89 ) |
|  | Unswitched memory B cell Absolute Count | Inverse variance weighted | 2 | 7.21E-01 | 1.05 (0.81 -1.35 ) |
|  | CD19 on IgD+ CD24- B cell | Inverse variance weighted | 4 | 7.21E-01 | 0.98 (0.86 -1.11 ) |
|  | CD14- CD16+ monocyte %monocyte | Wald ratio | 1 | 7.24E-01 | 1.04 (0.82 -1.33 ) |
|  | CD25 on switched memory B cell | Inverse variance weighted | 3 | 7.32E-01 | 0.98 (0.89 -1.08 ) |
|  | CD39+ CD8+ T cell %CD8+ T cell | Inverse variance weighted | 6 | 7.33E-01 | 0.99 (0.92 -1.06 ) |
|  | Effector Memory CD8+ T cell Absolute Count | Inverse variance weighted | 2 | 7.34E-01 | 1.04 (0.84 -1.29 ) |
|  | Monocyte Absolute Count | Wald ratio | 1 | 7.37E-01 | 1.05 (0.81 -1.35 ) |
|  | IgD+ CD24- B cell %B cell | Wald ratio | 1 | 7.37E-01 | 1.02 (0.89 -1.18 ) |
|  | CD20 on IgD- CD38dim B cell | Inverse variance weighted | 7 | 7.38E-01 | 0.99 (0.91 -1.07 ) |
|  | CD20 on unswitched memory B cell | Inverse variance weighted | 4 | 7.39E-01 | 0.97 (0.84 -1.13 ) |
|  | CD86 on CD62L+ myeloid Dendritic Cell | Wald ratio | 1 | 7.41E-01 | 1.02 (0.93 -1.11 ) |
|  | HLA DR+ T cell%lymphocyte | Inverse variance weighted | 4 | 7.43E-01 | 0.99 (0.91 -1.07 ) |
|  | CCR2 on monocyte | Wald ratio | 1 | 7.44E-01 | 1.03 (0.87 -1.21 ) |
|  | CD16-CD56 on Natural Killer | Inverse variance weighted | 8 | 7.45E-01 | 1.01 (0.95 -1.08 ) |
|  | CD25++ CD45RA- CD4 not regulatory T cell %T cell | Inverse variance weighted | 3 | 7.48E-01 | 1.01 (0.95 -1.07 ) |
|  | CD25 on B cell | Inverse variance weighted | 3 | 7.53E-01 | 0.98 (0.88 -1.09 ) |
|  | Activated & secreting CD4 regulatory T cell %CD4 regulatory T cell | Inverse variance weighted | 8 | 7.57E-01 | 1.01 (0.96 -1.06 ) |
|  | IgD on IgD+ CD24+ B cell | Inverse variance weighted | 6 | 7.58E-01 | 0.99 (0.91 -1.07 ) |
|  | CD19 on CD20- B cell | Wald ratio | 1 | 7.60E-01 | 1.02 (0.90 -1.15 ) |
|  | CD19 on switched memory B cell | Inverse variance weighted | 2 | 7.60E-01 | 0.92 (0.53 -1.60 ) |
|  | CD3 on naive CD8+ T cell | Inverse variance weighted | 4 | 7.61E-01 | 0.99 (0.91 -1.07 ) |
|  | Resting CD4 regulatory T cell Absolute Count | Inverse variance weighted | 4 | 7.67E-01 | 0.99 (0.93 -1.06 ) |
|  | CD28+ CD45RA- CD8+ T cell %T cell | Inverse variance weighted | 3 | 7.69E-01 | 0.99 (0.93 -1.05 ) |
|  | CD4-CD8- T cell %leukocyte | Inverse variance weighted | 4 | 7.70E-01 | 1.02 (0.89 -1.16 ) |
|  | CD19 on IgD+ CD38- unswitched memory B cell | Inverse variance weighted | 3 | 7.70E-01 | 1.03 (0.85 -1.24 ) |
|  | CD25++ CD45RA- CD4 not regulatory T cell %CD4+ T cell | Inverse variance weighted | 3 | 7.73E-01 | 0.99 (0.93 -1.05 ) |
|  | CD64 on CD14+ CD16- monocyte | Inverse variance weighted | 8 | 7.76E-01 | 1.01 (0.96 -1.05 ) |
|  | FSC-A on Natural Killer T | Inverse variance weighted | 2 | 7.80E-01 | 1.03 (0.83 -1.29 ) |
|  | B cell Absolute Count | Wald ratio | 1 | 7.81E-01 | 1.04 (0.78 -1.38 ) |
|  | CD20 on IgD+ CD38- B cell | Inverse variance weighted | 4 | 7.81E-01 | 0.98 (0.86 -1.12 ) |
|  | CD4-CD8- Natural Killer T %lymphocyte | Inverse variance weighted | 6 | 7.82E-01 | 1.01 (0.93 -1.11 ) |
|  | CD45 on Immature Myeloid-Derived Suppressor Cells | Inverse variance weighted | 2 | 7.83E-01 | 1.01 (0.95 -1.07 ) |
|  | Plasmacytoid Dendritic Cell Absolute Count | Inverse variance weighted | 2 | 7.86E-01 | 1.05 (0.73 -1.53 ) |
|  | CD20 on switched memory B cell | Inverse variance weighted | 7 | 7.88E-01 | 0.99 (0.91 -1.07 ) |
|  | CD4-CD8- T cell %T cell | Inverse variance weighted | 3 | 7.89E-01 | 1.02 (0.88 -1.18 ) |
|  | CD127 on CD28+ CD45RA+ CD8+ T cell | Inverse variance weighted | 2 | 7.92E-01 | 1.03 (0.82 -1.31 ) |
|  | CD64 on monocyte | Inverse variance weighted | 8 | 7.93E-01 | 1.01 (0.96 -1.06 ) |
|  | CD11c on CD62L+ myeloid Dendritic Cell | Wald ratio | 1 | 7.95E-01 | 1.01 (0.92 -1.12 ) |
|  | HVEM on naive CD8+ T cell | Inverse variance weighted | 2 | 7.97E-01 | 0.98 (0.87 -1.12 ) |
|  | HLA DR+ T cell%T cell | Inverse variance weighted | 4 | 7.98E-01 | 1.02 (0.90 -1.14 ) |
|  | CD127 on granulocyte | Inverse variance weighted | 8 | 7.98E-01 | 0.99 (0.94 -1.05 ) |
|  | SSC-A on myeloid Dendritic Cell | Inverse variance weighted | 5 | 8.01E-01 | 0.97 (0.80 -1.19 ) |
|  | CD25 on CD45RA- CD4 not regulatory T cell | Inverse variance weighted | 2 | 8.02E-01 | 0.98 (0.86 -1.13 ) |
|  | CD3- lymphocyte Absolute Count | Wald ratio | 1 | 8.04E-01 | 0.98 (0.83 -1.16 ) |
|  | CD45RA- CD28- CD8+ T cell %CD8+ T cell | Inverse variance weighted | 10 | 8.05E-01 | 1.00 (1.00 -1.00 ) |
|  | CD38 on IgD- CD38dim B cell | Inverse variance weighted | 3 | 8.06E-01 | 0.99 (0.91 -1.08 ) |
|  | FSC-A on CD14+ monocyte | Wald ratio | 1 | 8.07E-01 | 1.07 (0.63 -1.82 ) |
|  | CD86 on granulocyte | Inverse variance weighted | 2 | 8.08E-01 | 0.97 (0.74 -1.27 ) |
|  | Effector Memory CD4-CD8- T cell %T cell | Inverse variance weighted | 3 | 8.08E-01 | 1.02 (0.89 -1.17 ) |
|  | IgD- CD27- B cell Absolute Count | Inverse variance weighted | 2 | 8.08E-01 | 0.98 (0.82 -1.17 ) |
|  | CD8dim T cell %T cell | Inverse variance weighted | 2 | 8.09E-01 | 0.97 (0.79 -1.20 ) |
|  | CD16-CD56 on Natural Killer T | Inverse variance weighted | 4 | 8.09E-01 | 1.01 (0.93 -1.10 ) |
|  | CD8+ Natural Killer T %lymphocyte | Wald ratio | 1 | 8.09E-01 | 0.95 (0.65 -1.40 ) |
|  | Hematopoietic Stem Cell Absolute Count | Inverse variance weighted | 2 | 8.11E-01 | 0.99 (0.94 -1.05 ) |
|  | CD25 on IgD+ CD38- naive B cell | Inverse variance weighted | 3 | 8.12E-01 | 1.01 (0.92 -1.12 ) |
|  | Natural Killer %lymphocyte | Inverse variance weighted | 3 | 8.12E-01 | 0.99 (0.88 -1.11 ) |
|  | CD20 on IgD- CD38- B cell | Inverse variance weighted | 4 | 8.13E-01 | 1.02 (0.89 -1.16 ) |
|  | CD33dim HLA DR+ CD11b- Absolute Count | Inverse variance weighted | 2 | 8.13E-01 | 1.01 (0.90 -1.15 ) |
|  | Effector Memory CD8+ T cell %CD8+ T cell | Inverse variance weighted | 3 | 8.19E-01 | 0.99 (0.92 -1.06 ) |
|  | CCR2 on CD62L+ myeloid Dendritic Cell | Inverse variance weighted | 3 | 8.24E-01 | 0.99 (0.92 -1.07 ) |
|  | CD38 on naive-mature B cell | Inverse variance weighted | 3 | 8.25E-01 | 0.99 (0.90 -1.09 ) |
|  | CD8dim T cell %leukocyte | Inverse variance weighted | 2 | 8.26E-01 | 0.98 (0.78 -1.21 ) |
|  | Effector Memory CD8+ T cell %T cell | Inverse variance weighted | 2 | 8.27E-01 | 1.01 (0.93 -1.09 ) |
|  | IgD on IgD+ CD38+ B cell | Inverse variance weighted | 6 | 8.30E-01 | 1.00 (0.96 -1.05 ) |
|  | Lymphocyte %leukocyte | Wald ratio | 1 | 8.34E-01 | 1.04 (0.72 -1.49 ) |
|  | Granulocyte %leukocyte | Wald ratio | 1 | 8.34E-01 | 0.96 (0.67 -1.38 ) |
|  | CD19 on CD20- CD38- B cell | Wald ratio | 1 | 8.44E-01 | 1.03 (0.75 -1.41 ) |
|  | CD38 on transitional B cell | Inverse variance weighted | 5 | 8.46E-01 | 0.99 (0.92 -1.07 ) |
|  | FSC-A on granulocyte | Inverse variance weighted | 2 | 8.47E-01 | 0.99 (0.86 -1.13 ) |
|  | CD4 on CD39+ resting CD4 regulatory T cell | Wald ratio | 1 | 8.47E-01 | 0.98 (0.78 -1.23 ) |
|  | CD27 on unswitched memory B cell | Inverse variance weighted | 11 | 8.50E-01 | 0.99 (0.92 -1.07 ) |
|  | CD25 on transitional B cell | Inverse variance weighted | 2 | 8.54E-01 | 1.01 (0.88 -1.17 ) |
|  | CD28+ CD45RA+ CD8+ T cell %CD8+ T cell | Wald ratio | 1 | 8.54E-01 | 1.01 (0.90 -1.14 ) |
|  | CD27 on IgD+ CD38- unswitched memory B cell | Inverse variance weighted | 5 | 8.57E-01 | 1.01 (0.94 -1.08 ) |
|  | CD64 on CD14- CD16+ monocyte | Wald ratio | 1 | 8.64E-01 | 1.02 (0.79 -1.33 ) |
|  | CD45 on CD8+ T cell | Inverse variance weighted | 2 | 8.67E-01 | 1.01 (0.92 -1.10 ) |
|  | CX3CR1 on CD14- CD16- | Inverse variance weighted | 3 | 8.70E-01 | 1.01 (0.90 -1.14 ) |
|  | Terminally Differentiated CD4-CD8- T cell Absolute Count | Wald ratio | 1 | 8.71E-01 | 1.06 (0.52 -2.17 ) |
|  | CD62L on CD62L+ Dendritic Cell | Inverse variance weighted | 2 | 8.71E-01 | 0.99 (0.85 -1.14 ) |
|  | FSC-A on myeloid Dendritic Cell | Inverse variance weighted | 3 | 8.72E-01 | 1.01 (0.92 -1.11 ) |
|  | IgD on IgD+ CD38- unswitched memory B cell | Inverse variance weighted | 2 | 8.72E-01 | 1.00 (0.95 -1.06 ) |
|  | CCR2 on myeloid Dendritic Cell | Inverse variance weighted | 2 | 8.74E-01 | 1.01 (0.89 -1.14 ) |
|  | CD45 on CD33- HLA DR+ | Inverse variance weighted | 2 | 8.77E-01 | 1.02 (0.84 -1.23 ) |
|  | CCR2 on granulocyte | Inverse variance weighted | 2 | 8.79E-01 | 0.99 (0.83 -1.17 ) |
|  | CD8+ Natural Killer T %T cell | Wald ratio | 1 | 8.81E-01 | 0.97 (0.64 -1.46 ) |
|  | CD62L- monocyte Absolute Count | Inverse variance weighted | 2 | 8.87E-01 | 1.02 (0.77 -1.35 ) |
|  | CD38 on IgD+ CD38+ B cell | Inverse variance weighted | 3 | 8.90E-01 | 0.99 (0.89 -1.11 ) |
|  | Naive-mature B cell %lymphocyte | Wald ratio | 1 | 8.90E-01 | 1.01 (0.92 -1.11 ) |
|  | IgD+ CD24- B cell %lymphocyte | Wald ratio | 1 | 8.90E-01 | 1.01 (0.91 -1.11 ) |
|  | CD25 on resting CD4 regulatory T cell | Wald ratio | 1 | 8.93E-01 | 1.07 (0.41 -2.77 ) |
|  | CD4-CD8- Natural Killer T %T cell | Inverse variance weighted | 5 | 8.95E-01 | 1.01 (0.92 -1.10 ) |
|  | HLA DR on CD14- CD16- | Inverse variance weighted | 4 | 8.97E-01 | 0.99 (0.90 -1.10 ) |
|  | CD25 on CD39+ resting CD4 regulatory T cell | Wald ratio | 1 | 8.98E-01 | 0.98 (0.72 -1.33 ) |
|  | CD8 on CD8+ T cell | Wald ratio | 1 | 8.98E-01 | 1.01 (0.90 -1.13 ) |
|  | CD8 on Terminally Differentiated CD8+ T cell | Inverse variance weighted | 2 | 9.00E-01 | 0.99 (0.81 -1.21 ) |
|  | CD45RA- CD28- CD8+ T cell Absolute Count | Inverse variance weighted | 138 | 9.02E-01 | 1.00 (1.00 -1.00 ) |
|  | CD80 on CD62L+ myeloid Dendritic Cell | Inverse variance weighted | 3 | 9.04E-01 | 1.01 (0.86 -1.19 ) |
|  | CD25 on naive-mature B cell | Inverse variance weighted | 4 | 9.05E-01 | 1.01 (0.89 -1.14 ) |
|  | CD25 on IgD+ CD24- B cell | Inverse variance weighted | 3 | 9.07E-01 | 1.01 (0.90 -1.12 ) |
|  | CD27 on memory B cell | Inverse variance weighted | 7 | 9.07E-01 | 1.00 (0.92 -1.08 ) |
|  | CD8 on CD28- CD8+ T cell | Inverse variance weighted | 2 | 9.18E-01 | 1.02 (0.72 -1.45 ) |
|  | CD28+ CD45RA- CD8+ T cell %CD8+ T cell | Inverse variance weighted | 3 | 9.22E-01 | 0.99 (0.87 -1.13 ) |
|  | CD38 on IgD+ CD38dim B cell | Inverse variance weighted | 4 | 9.23E-01 | 1.00 (0.93 -1.09 ) |
|  | CD33+ HLA DR+ CD14dim Absolute Count | Inverse variance weighted | 2 | 9.23E-01 | 0.99 (0.78 -1.25 ) |
|  | CD3 on CD45RA+ CD4+ T cell | Inverse variance weighted | 4 | 9.23E-01 | 1.00 (0.91 -1.09 ) |
|  | Effector Memory CD4-CD8- T cell Absolute Count | Inverse variance weighted | 4 | 9.28E-01 | 1.00 (0.90 -1.12 ) |
|  | CD4 on CD39+ activated CD4 regulatory T cell | Inverse variance weighted | 3 | 9.31E-01 | 0.99 (0.82 -1.20 ) |
|  | IgD- CD27- B cell %B cell | Inverse variance weighted | 3 | 9.31E-01 | 1.00 (0.90 -1.12 ) |
|  | Activated & resting CD4 regulatory T cell %CD4+ T cell | Inverse variance weighted | 3 | 9.33E-01 | 1.00 (0.94 -1.06 ) |
|  | CD14 on CD14+ CD16- monocyte | Wald ratio | 1 | 9.34E-01 | 0.99 (0.85 -1.16 ) |
|  | IgD on IgD+ CD24- B cell | Inverse variance weighted | 5 | 9.36E-01 | 1.00 (0.94 -1.07 ) |
|  | IgD on IgD+ CD38dim B cell | Inverse variance weighted | 5 | 9.38E-01 | 1.00 (0.93 -1.08 ) |
|  | Unswitched memory B cell %B cell | Wald ratio | 1 | 9.42E-01 | 1.01 (0.79 -1.30 ) |
|  | Natural Killer Absolute Count | Inverse variance weighted | 3 | 9.46E-01 | 1.00 (0.87 -1.14 ) |
|  | CD4 on Effector Memory CD4+ T cell | Inverse variance weighted | 3 | 9.46E-01 | 1.00 (0.92 -1.08 ) |
|  | CD28- CD4-CD8- T cell %T cell | Inverse variance weighted | 3 | 9.50E-01 | 1.00 (0.88 -1.15 ) |
|  | Effector Memory CD4+ T cell %CD4+ T cell | Inverse variance weighted | 2 | 9.56E-01 | 1.01 (0.74 -1.37 ) |
|  | CD8 on Central Memory CD8+ T cell | Inverse variance weighted | 2 | 9.58E-01 | 1.00 (0.89 -1.11 ) |
|  | CD11b on CD33dim HLA DR- | Wald ratio | 1 | 9.64E-01 | 1.00 (0.90 -1.10 ) |
|  | CD11b on basophil | Wald ratio | 1 | 9.64E-01 | 1.00 (0.91 -1.10 ) |
|  | Effector Memory CD4-CD8- T cell %CD4-CD8- T cell | Inverse variance weighted | 4 | 9.79E-01 | 1.00 (0.84 -1.19 ) |
|  | CD8dim Natural Killer T Absolute Count | Inverse variance weighted | 5 | 9.80E-01 | 1.00 (0.91 -1.10 ) |
|  | CD24 on IgD+ CD38+ B cell | Inverse variance weighted | 2 | 9.80E-01 | 1.00 (0.82 -1.21 ) |
|  | CD14 on CD14+ CD16+ monocyte | Inverse variance weighted | 2 | 9.85E-01 | 1.00 (0.86 -1.16 ) |
|  | Unswitched memory B cell %lymphocyte | Inverse variance weighted | 4 | 9.86E-01 | 1.00 (0.85 -1.18 ) |
|  | CD38 on IgD+ CD24- B cell | Inverse variance weighted | 2 | 9.93E-01 | 1.00 (0.89 -1.13 ) |
|  | CD8dim T cell Absolute Count | Inverse variance weighted | 3 | 9.95E-01 | 1.00 (0.84 -1.19 ) |
|  | CD28- CD8dim T cell Absolute Count | Inverse variance weighted | 2 | 9.95E-01 | 1.00 (0.76 -1.31 ) |
|  | IgD on unswitched memory B cell | Inverse variance weighted | 5 | 9.99E-01 | 1.00 (0.95 -1.06 ) |

**Table S4. Two-sample Mendelian randomization estimations showing the effects of Immune cell traits on the risk of pre-eclampsia.**

| outcome | exposure | method | nsnp | pval | or |
| --- | --- | --- | --- | --- | --- |
| pre-eclampsia | Lymphocyte Absolute Count | Inverse variance weighted | 2 | 9.18E-07 | 1.50 (1.28 -1.77 ) |
|  | T cell Absolute Count | Inverse variance weighted | 2 | 1.76E-06 | 1.47 (1.25 -1.72 ) |
|  | CD28- CD25++ CD8+ T cell Absolute Count | Wald ratio | 1 | 6.55E-06 | 1.77 (1.38 -2.26 ) |
|  | Terminally Differentiated CD4+ T cell %CD4+ T cell | Inverse variance weighted | 4 | 6.32E-04 | 0.82 (0.74 -0.92 ) |
|  | Central Memory CD4+ T cell Absolute Count | Wald ratio | 1 | 1.35E-03 | 1.46 (1.16 -1.84 ) |
|  | CD4+ T cell Absolute Count | Wald ratio | 1 | 1.35E-03 | 1.39 (1.14 -1.71 ) |
|  | CD8+ T cell Absolute Count | Wald ratio | 1 | 2.69E-03 | 1.35 (1.11 -1.64 ) |
|  | CD8+ T cell %leukocyte | Wald ratio | 1 | 2.69E-03 | 1.37 (1.11 -1.68 ) |
|  | CD127- CD8+ T cell Absolute Count | Wald ratio | 1 | 2.69E-03 | 1.34 (1.11 -1.63 ) |
|  | SSC-A on CD14+ monocyte | Inverse variance weighted | 5 | 3.30E-03 | 0.91 (0.85 -0.97 ) |
|  | HLA DR on Dendritic Cell | Inverse variance weighted | 7 | 3.42E-03 | 1.06 (1.02 -1.10 ) |
|  | CD45 on Natural Killer T | Wald ratio | 1 | 4.36E-03 | 1.41 (1.11 -1.79 ) |
|  | Naive CD4-CD8- T cell %T cell | Wald ratio | 1 | 4.59E-03 | 0.67 (0.51 -0.89 ) |
|  | CD64 on CD14+ CD16+ monocyte | Inverse variance weighted | 2 | 4.75E-03 | 1.42 (1.11 -1.81 ) |
|  | FSC-A on CD4+ T cell | Inverse variance weighted | 3 | 5.50E-03 | 0.75 (0.62 -0.92 ) |
|  | PDL-1 on CD14- CD16- | Wald ratio | 1 | 5.84E-03 | 0.66 (0.49 -0.89 ) |
|  | CD28- CD127- CD25++ CD8+ T cell Absolute Count | Wald ratio | 1 | 5.94E-03 | 1.34 (1.09 -1.65 ) |
|  | CD3 on Effector Memory CD8+ T cell | Inverse variance weighted | 3 | 7.28E-03 | 0.87 (0.79 -0.96 ) |
|  | HLA DR on myeloid Dendritic Cell | Inverse variance weighted | 6 | 1.43E-02 | 1.06 (1.01 -1.11 ) |
|  | SSC-A on plasmacytoid Dendritic Cell | Inverse variance weighted | 5 | 1.47E-02 | 0.86 (0.77 -0.97 ) |
|  | IgD- CD38dim B cell %lymphocyte | Inverse variance weighted | 2 | 1.70E-02 | 1.28 (1.05 -1.57 ) |
|  | CD80 on monocyte | Inverse variance weighted | 6 | 1.85E-02 | 0.93 (0.88 -0.99 ) |
|  | CD4 on HLA DR+ CD4+ T cell | Inverse variance weighted | 2 | 2.06E-02 | 1.26 (1.04 -1.54 ) |
|  | CD45RA on naive CD8+ T cell | Inverse variance weighted | 2 | 2.20E-02 | 0.84 (0.73 -0.98 ) |
|  | HLA DR on plasmacytoid Dendritic Cell | Inverse variance weighted | 8 | 2.40E-02 | 1.04 (1.01 -1.08 ) |
|  | CD24 on memory B cell | Inverse variance weighted | 2 | 2.48E-02 | 0.87 (0.77 -0.98 ) |
|  | CD24 on switched memory B cell | Inverse variance weighted | 2 | 2.50E-02 | 0.87 (0.76 -0.98 ) |
|  | CD28 on CD39+ CD8+ T cell | Inverse variance weighted | 2 | 2.58E-02 | 1.05 (1.01 -1.10 ) |
|  | CD39+ secreting CD4 regulatory T cell %secreting CD4 regulatory T cell | Inverse variance weighted | 4 | 3.01E-02 | 1.04 (1.00 -1.08 ) |
|  | CD45 on B cell | Inverse variance weighted | 2 | 3.10E-02 | 1.25 (1.02 -1.53 ) |
|  | HLA DR on CD33dim HLA DR+ CD11b- | Inverse variance weighted | 5 | 3.43E-02 | 1.11 (1.01 -1.23 ) |
|  | CD28 on CD39+ secreting CD4 regulatory T cell | Inverse variance weighted | 2 | 3.50E-02 | 1.10 (1.01 -1.20 ) |
|  | B cell %CD3- lymphocyte | Inverse variance weighted | 3 | 3.55E-02 | 1.09 (1.01 -1.18 ) |
|  | BAFF-R on CD20- B cell | Wald ratio | 1 | 3.84E-02 | 1.18 (1.01 -1.37 ) |
|  | CD39+ CD8+ T cell %T cell | Inverse variance weighted | 8 | 3.90E-02 | 1.03 (1.00 -1.07 ) |
|  | BAFF-R on IgD+ CD38- unswitched memory B cell | Inverse variance weighted | 5 | 4.21E-02 | 1.04 (1.00 -1.09 ) |
|  | Switched memory B cell %lymphocyte | Inverse variance weighted | 3 | 4.28E-02 | 1.17 (1.01 -1.36 ) |
|  | CD14 on Monocytic Myeloid-Derived Suppressor Cells | Wald ratio | 1 | 4.41E-02 | 0.88 (0.77 -1.00 ) |
|  | CD40 on CD14+ CD16+ monocyte | Inverse variance weighted | 3 | 4.48E-02 | 1.07 (1.00 -1.15 ) |
|  | BAFF-R on CD20- CD38- B cell | Wald ratio | 1 | 4.49E-02 | 1.14 (1.00 -1.29 ) |
|  | CD80 on CD62L+ plasmacytoid Dendritic Cell | Inverse variance weighted | 2 | 4.90E-02 | 0.88 (0.77 -1.00 ) |
|  | CD80 on plasmacytoid Dendritic Cell | Inverse variance weighted | 2 | 4.93E-02 | 0.88 (0.78 -1.00 ) |
|  | CD123 on CD62L+ plasmacytoid Dendritic Cell | Inverse variance weighted | 2 | 4.96E-02 | 0.89 (0.79 -1.00 ) |
|  | CD40 on CD14+ CD16- monocyte | Inverse variance weighted | 3 | 4.98E-02 | 1.08 (1.00 -1.17 ) |
|  | CD123 on plasmacytoid Dendritic Cell | Inverse variance weighted | 2 | 4.99E-02 | 0.89 (0.79 -1.00 ) |
|  | Natural Killer %CD3- lymphocyte | Inverse variance weighted | 5 | 5.32E-02 | 0.93 (0.86 -1.00 ) |
|  | IgD- CD38- B cell Absolute Count | Wald ratio | 1 | 5.57E-02 | 0.83 (0.69 -1.00 ) |
|  | CD25 on CD39+ CD4+ T cell | Inverse variance weighted | 5 | 5.63E-02 | 0.95 (0.91 -1.00 ) |
|  | CD39+ CD8+ T cell Absolute Count | Inverse variance weighted | 7 | 5.64E-02 | 1.03 (1.00 -1.07 ) |
|  | CD27 on switched memory B cell | Inverse variance weighted | 9 | 5.64E-02 | 0.95 (0.90 -1.00 ) |
|  | Activated CD4 regulatory T cell Absolute Count | Inverse variance weighted | 2 | 5.80E-02 | 1.10 (1.00 -1.21 ) |
|  | CD27 on CD24+ CD27+ B cell | Inverse variance weighted | 9 | 5.82E-02 | 0.95 (0.90 -1.00 ) |
|  | CD39+ secreting CD4 regulatory T cell Absolute Count | Inverse variance weighted | 4 | 5.88E-02 | 1.04 (1.00 -1.07 ) |
|  | CD28- CD8+ T cell Absolute Count | Inverse variance weighted | 3 | 5.91E-02 | 1.21 (0.99 -1.48 ) |
|  | CD19 on IgD- CD38- B cell | Wald ratio | 1 | 6.03E-02 | 0.78 (0.60 -1.01 ) |
|  | CD28 on CD28+ CD45RA+ CD8+ T cell | Inverse variance weighted | 3 | 6.68E-02 | 1.09 (0.99 -1.20 ) |
|  | CD3 on CD39+ activated CD4 regulatory T cell | Inverse variance weighted | 4 | 6.82E-02 | 0.95 (0.89 -1.00 ) |
|  | CD28 on CD45RA+ CD4+ T cell | Inverse variance weighted | 2 | 7.05E-02 | 1.10 (0.99 -1.22 ) |
|  | Central Memory CD8+ T cell Absolute Count | Wald ratio | 1 | 7.26E-02 | 1.23 (0.98 -1.55 ) |
|  | CD45RA on naive CD4+ T cell | Inverse variance weighted | 10 | 7.28E-02 | 0.96 (0.93 -1.00 ) |
|  | CD28 on CD39+ CD4+ T cell | Inverse variance weighted | 5 | 7.43E-02 | 1.05 (0.99 -1.12 ) |
|  | BAFF-R on unswitched memory B cell | Inverse variance weighted | 7 | 7.62E-02 | 1.04 (1.00 -1.08 ) |
|  | BAFF-R on memory B cell | Inverse variance weighted | 7 | 7.62E-02 | 1.04 (1.00 -1.08 ) |
|  | CD3 on resting CD4 regulatory T cell | Inverse variance weighted | 4 | 7.82E-02 | 1.06 (0.99 -1.14 ) |
|  | BAFF-R on CD24+ CD27+ B cell | Inverse variance weighted | 8 | 7.91E-02 | 1.03 (1.00 -1.07 ) |
|  | Monocytic Myeloid-Derived Suppressor Cells Absolute Count | Inverse variance weighted | 5 | 8.16E-02 | 0.96 (0.91 -1.01 ) |
|  | BAFF-R on IgD- CD38dim B cell | Wald ratio | 1 | 8.17E-02 | 0.83 (0.67 -1.02 ) |
|  | CD27 on Plasma Blast-Plasma Cell | Wald ratio | 1 | 8.17E-02 | 0.80 (0.63 -1.03 ) |
|  | CD33dim HLA DR+ CD11b+ Absolute Count | Wald ratio | 1 | 8.29E-02 | 1.13 (0.98 -1.30 ) |
|  | CD27 on T cell | Wald ratio | 1 | 8.29E-02 | 0.90 (0.80 -1.01 ) |
|  | PDL-1 on CD14+ CD16+ monocyte | Wald ratio | 1 | 8.29E-02 | 1.25 (0.97 -1.61 ) |
|  | CX3CR1 on CD14- CD16+ monocyte | Wald ratio | 1 | 8.29E-02 | 0.87 (0.75 -1.02 ) |
|  | CD14 on CD33dim HLA DR+ CD11b+ | Wald ratio | 1 | 8.29E-02 | 0.91 (0.82 -1.01 ) |
|  | CD11c on monocyte | Wald ratio | 1 | 8.29E-02 | 1.12 (0.98 -1.28 ) |
|  | CD11b on CD14+ monocyte | Wald ratio | 1 | 8.29E-02 | 1.05 (0.99 -1.12 ) |
|  | CD11b on Monocytic Myeloid-Derived Suppressor Cells | Wald ratio | 1 | 8.29E-02 | 1.07 (0.99 -1.16 ) |
|  | CD11b on CD33+ HLA DR+ CD14dim | Wald ratio | 1 | 8.29E-02 | 1.07 (0.99 -1.15 ) |
|  | CD4 regulatory T cell %CD4+ T cell | Inverse variance weighted | 2 | 8.31E-02 | 1.31 (0.97 -1.77 ) |
|  | CD40 on CD14- CD16+ monocyte | Inverse variance weighted | 7 | 8.42E-02 | 1.05 (0.99 -1.12 ) |
|  | BAFF-R on IgD+ CD24+ B cell | Inverse variance weighted | 6 | 8.43E-02 | 1.03 (1.00 -1.08 ) |
|  | CD4 on CD39+ CD4+ T cell | Inverse variance weighted | 5 | 8.57E-02 | 1.06 (0.99 -1.13 ) |
|  | CD64 on CD14- CD16- | Inverse variance weighted | 3 | 8.70E-02 | 1.18 (0.98 -1.42 ) |
|  | BAFF-R on IgD- CD38- B cell | Inverse variance weighted | 7 | 8.85E-02 | 1.03 (0.99 -1.07 ) |
|  | CD28 on CD39+ activated CD4 regulatory T cell | Inverse variance weighted | 2 | 9.00E-02 | 1.13 (0.98 -1.30 ) |
|  | CD28+ CD45RA+ CD8dim T cell Absolute Count | Inverse variance weighted | 8 | 9.09E-02 | 1.01 (1.00 -1.01 ) |
|  | CD39+ secreting CD4 regulatory T cell %CD4 regulatory T cell | Inverse variance weighted | 6 | 9.57E-02 | 1.03 (1.00 -1.06 ) |
|  | CD27 on IgD- CD38dim B cell | Inverse variance weighted | 6 | 9.68E-02 | 0.95 (0.90 -1.01 ) |
|  | CD39+ CD4+ T cell %T cell | Inverse variance weighted | 6 | 9.89E-02 | 1.03 (0.99 -1.06 ) |
|  | CD39+ CD4+ T cell %CD4+ T cell | Inverse variance weighted | 6 | 9.90E-02 | 1.03 (0.99 -1.06 ) |
|  | CD39 on CD39+ activated CD4 regulatory T cell | Inverse variance weighted | 5 | 9.91E-02 | 1.02 (1.00 -1.05 ) |
|  | CD39+ activated CD4 regulatory T cell %CD4 regulatory T cell | Inverse variance weighted | 6 | 1.02E-01 | 1.03 (0.99 -1.07 ) |
|  | CD25 on CD39+ secreting CD4 regulatory T cell | Inverse variance weighted | 2 | 1.02E-01 | 0.90 (0.79 -1.02 ) |
|  | CD25 on secreting CD4 regulatory T cell | Inverse variance weighted | 2 | 1.03E-01 | 0.90 (0.80 -1.02 ) |
|  | CX3CR1 on CD14+ CD16+ monocyte | Inverse variance weighted | 3 | 1.04E-01 | 0.94 (0.88 -1.01 ) |
|  | CD33 on CD66b++ myeloid cell | Inverse variance weighted | 4 | 1.05E-01 | 0.97 (0.94 -1.01 ) |
|  | CD39 on CD39+ secreting CD4 regulatory T cell | Inverse variance weighted | 5 | 1.06E-01 | 1.03 (0.99 -1.06 ) |
|  | CD39 on CD39+ CD4+ T cell | Inverse variance weighted | 4 | 1.07E-01 | 1.03 (0.99 -1.06 ) |
|  | CD33 on Granulocytic Myeloid-Derived Suppressor Cells | Inverse variance weighted | 3 | 1.09E-01 | 0.97 (0.93 -1.01 ) |
|  | CD25++ CD45RA+ CD4 not regulatory T cell Absolute Count | Inverse variance weighted | 3 | 1.11E-01 | 0.93 (0.85 -1.02 ) |
|  | HLA DR on HLA DR+ T cell | Wald ratio | 1 | 1.13E-01 | 1.14 (0.97 -1.34 ) |
|  | HLA DR on HLA DR+ CD4+ T cell | Wald ratio | 1 | 1.13E-01 | 1.21 (0.96 -1.52 ) |
|  | Naive CD8+ T cell %CD8+ T cell | Wald ratio | 1 | 1.17E-01 | 0.75 (0.53 -1.07 ) |
|  | SSC-A on monocyte | Inverse variance weighted | 7 | 1.19E-01 | 0.95 (0.89 -1.01 ) |
|  | SSC-A on lymphocyte | Wald ratio | 1 | 1.20E-01 | 0.84 (0.67 -1.05 ) |
|  | CD19 on B cell | Wald ratio | 1 | 1.22E-01 | 0.83 (0.66 -1.05 ) |
|  | CD38 on IgD+ B cell | Wald ratio | 1 | 1.24E-01 | 1.22 (0.95 -1.57 ) |
|  | CD4 on monocyte | Wald ratio | 1 | 1.26E-01 | 1.14 (0.96 -1.34 ) |
|  | CD25 on unswitched memory B cell | Inverse variance weighted | 4 | 1.26E-01 | 1.07 (0.98 -1.17 ) |
|  | Terminally Differentiated CD4-CD8- T cell %CD4-CD8- T cell | Inverse variance weighted | 2 | 1.28E-01 | 0.89 (0.77 -1.03 ) |
|  | IgD+ B cell %Lymphocyte | Inverse variance weighted | 2 | 1.29E-01 | 1.20 (0.95 -1.51 ) |
|  | HLA DR on CD33dim HLA DR+ CD11b+ | Inverse variance weighted | 4 | 1.29E-01 | 1.10 (0.97 -1.23 ) |
|  | CD33+ HLA DR+ CD14- %CD33+ HLA DR+ | Wald ratio | 1 | 1.29E-01 | 0.91 (0.81 -1.03 ) |
|  | CD33+ HLA DR+ Absolute Count | Wald ratio | 1 | 1.29E-01 | 0.92 (0.83 -1.02 ) |
|  | CD33+ HLA DR+ CD14- Absolute Count | Wald ratio | 1 | 1.29E-01 | 0.92 (0.84 -1.02 ) |
|  | CD25 on memory B cell | Inverse variance weighted | 4 | 1.30E-01 | 1.07 (0.98 -1.16 ) |
|  | CD4+ CD8dim T cell %lymphocyte | Inverse variance weighted | 2 | 1.32E-01 | 0.92 (0.83 -1.02 ) |
|  | CD8+ Natural Killer T Absolute Count | Inverse variance weighted | 2 | 1.32E-01 | 1.48 (0.89 -2.45 ) |
|  | CD25 on CD24+ CD27+ B cell | Inverse variance weighted | 4 | 1.32E-01 | 1.07 (0.98 -1.16 ) |
|  | CD4+ CD8dim T cell %leukocyte | Inverse variance weighted | 2 | 1.37E-01 | 0.92 (0.83 -1.03 ) |
|  | Resting CD4 regulatory T cell %CD4+ T cell | Inverse variance weighted | 5 | 1.37E-01 | 0.97 (0.92 -1.01 ) |
|  | CD28+ CD45RA+ CD8+ T cell %T cell | Inverse variance weighted | 37 | 1.39E-01 | 1.00 (0.99 -1.00 ) |
|  | CX3CR1 on monocyte | Inverse variance weighted | 3 | 1.40E-01 | 0.95 (0.88 -1.02 ) |
|  | CX3CR1 on CD14+ CD16- monocyte | Inverse variance weighted | 3 | 1.41E-01 | 0.95 (0.89 -1.02 ) |
|  | BAFF-R on switched memory B cell | Inverse variance weighted | 9 | 1.43E-01 | 1.03 (0.99 -1.07 ) |
|  | CD62L- plasmacytoid Dendritic Cell Absolute Count | Wald ratio | 1 | 1.47E-01 | 0.89 (0.77 -1.04 ) |
|  | CD25 on activated & secreting CD4 regulatory T cell | Wald ratio | 1 | 1.47E-01 | 0.88 (0.75 -1.04 ) |
|  | CD4+ CD8dim T cell Absolute Count | Inverse variance weighted | 2 | 1.47E-01 | 0.92 (0.82 -1.03 ) |
|  | HLA DR+ CD8+ T cell %lymphocyte | Inverse variance weighted | 2 | 1.50E-01 | 0.94 (0.86 -1.02 ) |
|  | CD62L on CD62L+ plasmacytoid Dendritic Cell | Inverse variance weighted | 3 | 1.52E-01 | 0.93 (0.84 -1.03 ) |
|  | IgD+ CD24+ B cell %lymphocyte | Inverse variance weighted | 2 | 1.52E-01 | 1.18 (0.94 -1.47 ) |
|  | CD8 on Effector Memory CD8+ T cell | Inverse variance weighted | 3 | 1.54E-01 | 0.94 (0.86 -1.02 ) |
|  | HLA DR on CD14+ CD16+ monocyte | Inverse variance weighted | 4 | 1.54E-01 | 1.04 (0.98 -1.10 ) |
|  | CD4 on naive CD4+ T cell | Inverse variance weighted | 5 | 1.55E-01 | 1.12 (0.96 -1.31 ) |
|  | BAFF-R on IgD- CD24- B cell | Inverse variance weighted | 8 | 1.56E-01 | 1.03 (0.99 -1.07 ) |
|  | CD3 on HLA DR+ T cell | Inverse variance weighted | 2 | 1.56E-01 | 0.93 (0.85 -1.03 ) |
|  | CD33 on CD33dim HLA DR+ CD11b+ | Inverse variance weighted | 6 | 1.57E-01 | 0.98 (0.94 -1.01 ) |
|  | BAFF-R on IgD- CD27- B cell | Inverse variance weighted | 8 | 1.58E-01 | 1.03 (0.99 -1.07 ) |
|  | HLA DR+ CD8+ T cell %T cell | Inverse variance weighted | 3 | 1.64E-01 | 0.94 (0.86 -1.02 ) |
|  | CD14- CD16- Absolute Count | Inverse variance weighted | 3 | 1.66E-01 | 1.18 (0.93 -1.48 ) |
|  | HLA DR+ Natural Killer %Natural Killer | Inverse variance weighted | 5 | 1.67E-01 | 1.05 (0.98 -1.12 ) |
|  | CD33 on CD33dim HLA DR+ CD11b- | Inverse variance weighted | 7 | 1.69E-01 | 0.98 (0.95 -1.01 ) |
|  | CD3 on activated & secreting CD4 regulatory T cell | Inverse variance weighted | 4 | 1.75E-01 | 0.96 (0.91 -1.02 ) |
|  | Natural Killer T %T cell | Inverse variance weighted | 5 | 1.75E-01 | 0.93 (0.83 -1.03 ) |
|  | CD34 on Hematopoietic Stem Cell | Inverse variance weighted | 4 | 1.76E-01 | 1.06 (0.97 -1.17 ) |
|  | CD3 on activated CD4 regulatory T cell | Inverse variance weighted | 4 | 1.76E-01 | 0.96 (0.91 -1.02 ) |
|  | CD45RA+ CD28- CD8+ T cell %CD8+ T cell | Wald ratio | 1 | 1.77E-01 | 0.99 (0.98 -1.00 ) |
|  | CD3 on CD39+ secreting CD4 regulatory T cell | Inverse variance weighted | 2 | 1.77E-01 | 0.95 (0.89 -1.02 ) |
|  | Plasma Blast-Plasma Cell %lymphocyte | Wald ratio | 1 | 1.80E-01 | 0.82 (0.62 -1.09 ) |
|  | CD11c on myeloid Dendritic Cell | Inverse variance weighted | 5 | 1.81E-01 | 0.92 (0.81 -1.04 ) |
|  | CD45RA on resting CD4 regulatory T cell | Inverse variance weighted | 6 | 1.83E-01 | 0.97 (0.92 -1.02 ) |
|  | CD24+ CD27+ B cell %lymphocyte | Inverse variance weighted | 3 | 1.87E-01 | 1.13 (0.94 -1.36 ) |
|  | CD27 on IgD- CD38- B cell | Inverse variance weighted | 7 | 1.92E-01 | 0.96 (0.89 -1.02 ) |
|  | CD3 on CD39+ CD4+ T cell | Inverse variance weighted | 4 | 1.92E-01 | 0.96 (0.91 -1.02 ) |
|  | CCR7 on naive CD4+ T cell | Inverse variance weighted | 4 | 1.95E-01 | 1.06 (0.97 -1.16 ) |
|  | CD62L- myeloid Dendritic Cell %Dendritic Cell | Inverse variance weighted | 4 | 1.96E-01 | 0.96 (0.90 -1.02 ) |
|  | HLA DR on CD14- CD16+ monocyte | Inverse variance weighted | 8 | 1.97E-01 | 1.06 (0.97 -1.15 ) |
|  | CD28 on CD28+ CD4+ T cell | Inverse variance weighted | 2 | 1.98E-01 | 1.08 (0.96 -1.21 ) |
|  | Terminally Differentiated CD4+ T cell Absolute Count | Inverse variance weighted | 2 | 1.98E-01 | 0.82 (0.61 -1.11 ) |
|  | HLA DR++ monocyte Absolute Count | Wald ratio | 1 | 2.00E-01 | 1.11 (0.95 -1.30 ) |
|  | Granulocytic Myeloid-Derived Suppressor Cells Absolute Count | Inverse variance weighted | 2 | 2.01E-01 | 0.92 (0.82 -1.04 ) |
|  | CD33 on Monocytic Myeloid-Derived Suppressor Cells | Inverse variance weighted | 4 | 2.01E-01 | 0.98 (0.95 -1.01 ) |
|  | HLA DR+ Natural Killer Absolute Count | Inverse variance weighted | 3 | 2.04E-01 | 1.05 (0.97 -1.14 ) |
|  | CD33 on CD14+ monocyte | Inverse variance weighted | 5 | 2.04E-01 | 0.98 (0.95 -1.01 ) |
|  | CD33 on CD33+ HLA DR+ CD14dim | Inverse variance weighted | 4 | 2.05E-01 | 0.98 (0.95 -1.01 ) |
|  | CD33 on CD33+ HLA DR+ | Inverse variance weighted | 4 | 2.06E-01 | 0.98 (0.95 -1.01 ) |
|  | HLA DR on B cell | Inverse variance weighted | 9 | 2.06E-01 | 1.03 (0.98 -1.08 ) |
|  | CD28 on CD4+ T cell | Inverse variance weighted | 2 | 2.07E-01 | 1.08 (0.96 -1.23 ) |
|  | CD33 on CD33+ HLA DR+ CD14- | Inverse variance weighted | 4 | 2.08E-01 | 0.98 (0.95 -1.01 ) |
|  | CD62L on CD62L+ myeloid Dendritic Cell | Wald ratio | 1 | 2.10E-01 | 1.14 (0.93 -1.39 ) |
|  | CD28 on secreting CD4 regulatory T cell | Inverse variance weighted | 2 | 2.10E-01 | 1.12 (0.94 -1.33 ) |
|  | Memory B cell %lymphocyte | Inverse variance weighted | 2 | 2.12E-01 | 1.16 (0.92 -1.47 ) |
|  | HLA DR on CD33+ HLA DR+ CD14- | Inverse variance weighted | 2 | 2.13E-01 | 1.04 (0.98 -1.11 ) |
|  | CD16 on CD14- CD16+ monocyte | Inverse variance weighted | 7 | 2.15E-01 | 1.03 (0.98 -1.07 ) |
|  | CD62L- Dendritic Cell Absolute Count | Inverse variance weighted | 3 | 2.19E-01 | 0.96 (0.89 -1.03 ) |
|  | CD28+ CD45RA+ CD8+ T cell Absolute Count | Inverse variance weighted | 3 | 2.22E-01 | 1.32 (0.85 -2.06 ) |
|  | CD62L- CD86+ myeloid Dendritic Cell Absolute Count | Inverse variance weighted | 3 | 2.24E-01 | 0.96 (0.91 -1.02 ) |
|  | CD86+ myeloid Dendritic Cell Absolute Count | Inverse variance weighted | 3 | 2.25E-01 | 0.96 (0.90 -1.03 ) |
|  | CCR2 on CD14+ CD16+ monocyte | Wald ratio | 1 | 2.26E-01 | 0.79 (0.53 -1.16 ) |
|  | CD86+ myeloid Dendritic Cell %Dendritic Cell | Inverse variance weighted | 3 | 2.27E-01 | 0.96 (0.90 -1.03 ) |
|  | CD28+ CD45RA- CD8dim T cell Absolute Count | Inverse variance weighted | 3 | 2.29E-01 | 1.03 (0.98 -1.08 ) |
|  | CD25 on IgD+ CD24+ B cell | Inverse variance weighted | 3 | 2.29E-01 | 1.06 (0.96 -1.17 ) |
|  | CD45 on HLA DR+ T cell | Wald ratio | 1 | 2.30E-01 | 0.91 (0.79 -1.06 ) |
|  | SSC-A on HLA DR+ T cell | Wald ratio | 1 | 2.30E-01 | 0.89 (0.73 -1.08 ) |
|  | CD33dim HLA DR+ CD11b- %CD33dim HLA DR+ | Inverse variance weighted | 3 | 2.30E-01 | 0.97 (0.92 -1.02 ) |
|  | B cell %lymphocyte | Inverse variance weighted | 2 | 2.30E-01 | 1.14 (0.92 -1.42 ) |
|  | CD25++ CD45RA+ CD4 not regulatory T cell %CD4+ T cell | Inverse variance weighted | 4 | 2.33E-01 | 0.96 (0.91 -1.02 ) |
|  | CD62L- myeloid Dendritic Cell Absolute Count | Inverse variance weighted | 5 | 2.33E-01 | 0.96 (0.90 -1.03 ) |
|  | CD33dim HLA DR+ CD11b+ %CD33dim HLA DR+ | Inverse variance weighted | 3 | 2.33E-01 | 1.03 (0.98 -1.09 ) |
|  | CD4+ T cell %leukocyte | Wald ratio | 1 | 2.34E-01 | 1.24 (0.87 -1.78 ) |
|  | T/B cell | Inverse variance weighted | 2 | 2.34E-01 | 0.88 (0.71 -1.09 ) |
|  | CD16 on CD14+ CD16+ monocyte | Inverse variance weighted | 4 | 2.38E-01 | 1.04 (0.97 -1.11 ) |
|  | CD25++ CD45RA+ CD4 not regulatory T cell %T cell | Inverse variance weighted | 4 | 2.39E-01 | 0.96 (0.90 -1.03 ) |
|  | Resting CD4 regulatory T cell %CD4 regulatory T cell | Inverse variance weighted | 9 | 2.39E-01 | 0.98 (0.94 -1.02 ) |
|  | CD24 on IgD- CD38- B cell | Inverse variance weighted | 2 | 2.41E-01 | 0.92 (0.81 -1.05 ) |
|  | CD62L- CD86+ myeloid Dendritic Cell %Dendritic Cell | Inverse variance weighted | 4 | 2.47E-01 | 0.97 (0.91 -1.02 ) |
|  | CD3 on HLA DR+ CD4+ T cell | Inverse variance weighted | 2 | 2.54E-01 | 0.95 (0.87 -1.04 ) |
|  | Activated & secreting CD4 regulatory T cell Absolute Count | Inverse variance weighted | 4 | 2.54E-01 | 1.03 (0.98 -1.08 ) |
|  | CD33- HLA DR+ Absolute Count | Wald ratio | 1 | 2.54E-01 | 0.91 (0.78 -1.07 ) |
|  | CD45 on CD33- HLA DR- | Inverse variance weighted | 2 | 2.55E-01 | 1.07 (0.95 -1.20 ) |
|  | CD24 on IgD+ CD38- B cell | Inverse variance weighted | 2 | 2.60E-01 | 0.91 (0.77 -1.07 ) |
|  | CD28+ CD45RA+ CD8dim T cell %T cell | Inverse variance weighted | 6 | 2.61E-01 | 0.97 (0.93 -1.02 ) |
|  | CD20 on IgD- CD27- B cell | Inverse variance weighted | 4 | 2.65E-01 | 0.93 (0.82 -1.06 ) |
|  | CCR2 on CD14- CD16- | Inverse variance weighted | 3 | 2.67E-01 | 0.86 (0.67 -1.12 ) |
|  | Secreting CD4 regulatory T cell Absolute Count | Inverse variance weighted | 4 | 2.67E-01 | 1.03 (0.98 -1.08 ) |
|  | CD33 on CD33dim HLA DR- | Inverse variance weighted | 6 | 2.68E-01 | 0.98 (0.95 -1.02 ) |
|  | HLA DR on HLA DR+ Natural Killer | Inverse variance weighted | 5 | 2.73E-01 | 1.07 (0.95 -1.19 ) |
|  | Effector Memory CD4+ T cell Absolute Count | Wald ratio | 1 | 2.74E-01 | 1.08 (0.94 -1.23 ) |
|  | CD25++ CD8+ T cell %CD8+ T cell | Wald ratio | 1 | 2.75E-01 | 1.07 (0.95 -1.20 ) |
|  | Secreting CD4 regulatory T cell %CD4+ T cell | Inverse variance weighted | 7 | 2.77E-01 | 1.02 (0.99 -1.05 ) |
|  | HLA DR on CD33- HLA DR+ | Inverse variance weighted | 2 | 2.84E-01 | 1.05 (0.96 -1.15 ) |
|  | CD45RA- CD4+ T cell Absolute Count | Inverse variance weighted | 2 | 2.84E-01 | 1.16 (0.88 -1.52 ) |
|  | IgD+ CD38+ B cell %lymphocyte | Wald ratio | 1 | 2.86E-01 | 0.56 (0.19 -1.63 ) |
|  | CD80 on granulocyte | Inverse variance weighted | 3 | 2.87E-01 | 1.08 (0.94 -1.23 ) |
|  | BAFF-R on transitional B cell | Inverse variance weighted | 8 | 2.88E-01 | 1.02 (0.98 -1.07 ) |
|  | CD45 on CD14+ monocyte | Wald ratio | 1 | 2.90E-01 | 1.26 (0.82 -1.92 ) |
|  | CD24 on transitional B cell | Wald ratio | 1 | 2.91E-01 | 0.88 (0.69 -1.12 ) |
|  | CD11c+ HLA DR++ monocyte %monocyte | Wald ratio | 1 | 2.92E-01 | 0.88 (0.70 -1.11 ) |
|  | CD8 on CD39+ CD8+ T cell | Inverse variance weighted | 5 | 2.92E-01 | 1.08 (0.94 -1.25 ) |
|  | HLA DR+ Natural Killer %CD3- lymphocyte | Inverse variance weighted | 2 | 2.92E-01 | 1.04 (0.96 -1.13 ) |
|  | PDL-1 on CD14+ CD16- monocyte | Wald ratio | 1 | 2.94E-01 | 0.87 (0.67 -1.13 ) |
|  | CD3 on Effector Memory CD4+ T cell | Inverse variance weighted | 2 | 2.96E-01 | 0.96 (0.89 -1.04 ) |
|  | Myeloid Dendritic Cell Absolute Count | Inverse variance weighted | 2 | 2.97E-01 | 0.96 (0.88 -1.04 ) |
|  | BAFF-R on IgD+ CD38- B cell | Inverse variance weighted | 9 | 2.99E-01 | 1.02 (0.98 -1.06 ) |
|  | CD45 on HLA DR+ CD4+ T cell | Inverse variance weighted | 2 | 3.01E-01 | 0.91 (0.77 -1.09 ) |
|  | Myeloid Dendritic Cell %Dendritic Cell | Inverse variance weighted | 2 | 3.01E-01 | 0.95 (0.87 -1.04 ) |
|  | CD3 on CD28+ CD45RA- CD8+ T cell | Inverse variance weighted | 4 | 3.03E-01 | 0.95 (0.87 -1.04 ) |
|  | CD45 on granulocyte | Wald ratio | 1 | 3.07E-01 | 0.88 (0.70 -1.12 ) |
|  | CD8dim Natural Killer T %T cell | Inverse variance weighted | 5 | 3.07E-01 | 0.95 (0.87 -1.05 ) |
|  | CD39+ resting CD4 regulatory T cell %CD4 regulatory T cell | Inverse variance weighted | 11 | 3.13E-01 | 1.02 (0.98 -1.06 ) |
|  | CD3 on CD45RA- CD4+ T cell | Inverse variance weighted | 2 | 3.16E-01 | 0.96 (0.90 -1.04 ) |
|  | CD11c on granulocyte | Inverse variance weighted | 2 | 3.16E-01 | 1.06 (0.95 -1.19 ) |
|  | BAFF-R on IgD- CD38+ B cell | Inverse variance weighted | 3 | 3.16E-01 | 1.07 (0.94 -1.23 ) |
|  | CD4+CD8+ T cell %leukocyte | Wald ratio | 1 | 3.17E-01 | 0.94 (0.82 -1.07 ) |
|  | CD3 on Naive CD4+ T cell | Inverse variance weighted | 2 | 3.18E-01 | 0.97 (0.92 -1.03 ) |
|  | CD28 on resting CD4 regulatory T cell | Wald ratio | 1 | 3.21E-01 | 1.06 (0.95 -1.18 ) |
|  | CD39 on monocyte | Inverse variance weighted | 2 | 3.24E-01 | 0.89 (0.72 -1.12 ) |
|  | CD16-CD56 on HLA DR+ Natural Killer | Inverse variance weighted | 2 | 3.26E-01 | 0.89 (0.72 -1.12 ) |
|  | Immature Myeloid-Derived Suppressor Cells Absolute Count | Inverse variance weighted | 2 | 3.26E-01 | 0.96 (0.88 -1.04 ) |
|  | Terminally Differentiated CD8+ T cell %CD8+ T cell | Inverse variance weighted | 3 | 3.28E-01 | 0.95 (0.85 -1.06 ) |
|  | CD20 on CD24+ CD27+ B cell | Inverse variance weighted | 5 | 3.29E-01 | 0.95 (0.86 -1.05 ) |
|  | Activated CD4 regulatory T cell %CD4 regulatory T cell | Inverse variance weighted | 3 | 3.30E-01 | 1.03 (0.97 -1.10 ) |
|  | BAFF-R on IgD+ CD38- naive B cell | Inverse variance weighted | 8 | 3.31E-01 | 1.02 (0.98 -1.06 ) |
|  | CD45RA- CD4+ T cell %CD4+ T cell | Inverse variance weighted | 4 | 3.33E-01 | 1.03 (0.97 -1.09 ) |
|  | CD20 on memory B cell | Inverse variance weighted | 5 | 3.33E-01 | 0.94 (0.84 -1.06 ) |
|  | Memory B cell %B cell | Inverse variance weighted | 2 | 3.36E-01 | 0.90 (0.72 -1.12 ) |
|  | HLA DR+ CD4+ T cell %T cell | Wald ratio | 1 | 3.38E-01 | 0.93 (0.80 -1.08 ) |
|  | CCR2 on CD62L+ plasmacytoid Dendritic Cell | Inverse variance weighted | 2 | 3.38E-01 | 0.96 (0.87 -1.05 ) |
|  | CCR2 on plasmacytoid Dendritic Cell | Inverse variance weighted | 2 | 3.38E-01 | 0.96 (0.87 -1.05 ) |
|  | SSC-A on T cell | Wald ratio | 1 | 3.42E-01 | 1.21 (0.82 -1.79 ) |
|  | CD3 on Terminally Differentiated CD4+ T cell | Wald ratio | 1 | 3.42E-01 | 0.93 (0.80 -1.08 ) |
|  | CD3 on Central Memory CD8+ T cell | Wald ratio | 1 | 3.42E-01 | 0.95 (0.86 -1.05 ) |
|  | CD3 on CD8+ T cell | Wald ratio | 1 | 3.42E-01 | 0.95 (0.86 -1.05 ) |
|  | CD3 on CD39+ CD8+ T cell | Wald ratio | 1 | 3.42E-01 | 0.94 (0.84 -1.06 ) |
|  | IgD+ CD38- B cell %B cell | Inverse variance weighted | 2 | 3.44E-01 | 0.85 (0.61 -1.19 ) |
|  | CD24 on IgD+ CD38- unswitched memory B cell | Inverse variance weighted | 2 | 3.47E-01 | 0.95 (0.86 -1.05 ) |
|  | CD4RA on Terminally Differentiated CD4+ T cell | Inverse variance weighted | 3 | 3.48E-01 | 0.97 (0.90 -1.04 ) |
|  | CD45RA- CD4+ T cell %T cell | Inverse variance weighted | 2 | 3.48E-01 | 1.06 (0.94 -1.19 ) |
|  | CD3 on Central Memory CD4+ T cell | Inverse variance weighted | 2 | 3.50E-01 | 0.97 (0.91 -1.04 ) |
|  | CD4-CD8- Natural Killer T Absolute Count | Wald ratio | 1 | 3.52E-01 | 1.11 (0.89 -1.39 ) |
|  | CD28+ CD4-CD8- T cell %T cell | Wald ratio | 1 | 3.52E-01 | 1.14 (0.86 -1.52 ) |
|  | CD3 on HLA DR+ CD8+ T cell | Wald ratio | 1 | 3.52E-01 | 0.93 (0.80 -1.08 ) |
|  | CD28 on activated & secreting CD4 regulatory T cell | Wald ratio | 1 | 3.54E-01 | 1.07 (0.93 -1.22 ) |
|  | CD28 on activated CD4 regulatory T cell | Wald ratio | 1 | 3.54E-01 | 1.08 (0.92 -1.26 ) |
|  | CD3 on CD28- CD8+ T cell | Inverse variance weighted | 2 | 3.55E-01 | 0.94 (0.82 -1.07 ) |
|  | CD33+ HLA DR+ CD14dim %CD33+ HLA DR+ | Wald ratio | 1 | 3.57E-01 | 1.08 (0.92 -1.27 ) |
|  | CCR7 on naive CD8+ T cell | Inverse variance weighted | 2 | 3.62E-01 | 1.07 (0.92 -1.25 ) |
|  | CD25 on IgD+ CD38- unswitched memory B cell | Wald ratio | 1 | 3.64E-01 | 1.05 (0.95 -1.16 ) |
|  | CD3 on CD39+ resting CD4 regulatory T cell | Wald ratio | 1 | 3.65E-01 | 0.94 (0.83 -1.07 ) |
|  | IgD+ CD38- B cell %lymphocyte | Inverse variance weighted | 2 | 3.67E-01 | 1.21 (0.80 -1.85 ) |
|  | FSC-A on HLA DR+ Natural Killer | Inverse variance weighted | 3 | 3.68E-01 | 1.04 (0.95 -1.14 ) |
|  | CD24 on unswitched memory B cell | Inverse variance weighted | 2 | 3.69E-01 | 0.94 (0.83 -1.07 ) |
|  | CD20 on transitional B cell | Inverse variance weighted | 2 | 3.75E-01 | 0.94 (0.83 -1.07 ) |
|  | HLA DR on CD33+ HLA DR+ CD14dim | Inverse variance weighted | 3 | 3.76E-01 | 1.03 (0.96 -1.11 ) |
|  | CD4+CD8+ T cell Absolute Count | Inverse variance weighted | 2 | 3.82E-01 | 0.94 (0.83 -1.08 ) |
|  | CD40 on monocytes | Inverse variance weighted | 4 | 3.82E-01 | 1.04 (0.96 -1.12 ) |
|  | CD28 on CD4 regulatory T cell | Wald ratio | 1 | 3.85E-01 | 1.07 (0.92 -1.25 ) |
|  | CD24 on IgD+ CD24+ B cell | Inverse variance weighted | 2 | 3.87E-01 | 0.95 (0.84 -1.07 ) |
|  | CD8+ and CD8dim T cell %leukocyte | Inverse variance weighted | 2 | 3.90E-01 | 1.10 (0.88 -1.37 ) |
|  | CD19 on IgD- CD38dim B cell | Inverse variance weighted | 4 | 3.90E-01 | 0.95 (0.86 -1.06 ) |
|  | CD86 on myeloid Dendritic Cell | Wald ratio | 1 | 3.90E-01 | 0.95 (0.85 -1.07 ) |
|  | Naive CD4+ T cell %CD4+ T cell | Inverse variance weighted | 4 | 3.91E-01 | 1.10 (0.89 -1.35 ) |
|  | CD39+ resting CD4 regulatory T cell Absolute Count | Inverse variance weighted | 8 | 3.92E-01 | 1.03 (0.97 -1.09 ) |
|  | CD3 on CD28+ CD4+ T cell | Inverse variance weighted | 2 | 3.94E-01 | 0.97 (0.90 -1.04 ) |
|  | CD3 on Terminally Differentiated CD8+ T cell | Inverse variance weighted | 2 | 3.95E-01 | 0.92 (0.77 -1.11 ) |
|  | CD4-CD8- T cell Absolute Count | Inverse variance weighted | 2 | 3.97E-01 | 1.08 (0.91 -1.27 ) |
|  | IgD- CD27- B cell %lymphocyte | Inverse variance weighted | 2 | 3.98E-01 | 1.06 (0.93 -1.20 ) |
|  | SSC-A on granulocyte | Inverse variance weighted | 3 | 3.99E-01 | 0.95 (0.85 -1.06 ) |
|  | CD4+/CD8+ T cell | Wald ratio | 1 | 4.01E-01 | 0.84 (0.56 -1.26 ) |
|  | CD8+ T cell %T cell | Wald ratio | 1 | 4.01E-01 | 1.19 (0.80 -1.77 ) |
|  | CD19 on IgD+ CD24+ B cell | Inverse variance weighted | 4 | 4.03E-01 | 0.96 (0.86 -1.06 ) |
|  | CD4 on CD45RA+ CD4+ T cell | Inverse variance weighted | 2 | 4.05E-01 | 0.96 (0.86 -1.06 ) |
|  | Terminally Differentiated CD8+ T cell %T cell | Inverse variance weighted | 2 | 4.05E-01 | 0.94 (0.82 -1.08 ) |
|  | CD4 on Central Memory CD4+ T cell | Inverse variance weighted | 2 | 4.07E-01 | 0.95 (0.85 -1.07 ) |
|  | CD45RA+ CD28- CD8+ T cell Absolute Count | Inverse variance weighted | 417 | 4.14E-01 | 1.00 (1.00 -1.00 ) |
|  | CD4 regulatory T cell %T cell | Wald ratio | 1 | 4.14E-01 | 1.15 (0.82 -1.62 ) |
|  | CD20 on IgD+ CD24+ B cell | Inverse variance weighted | 2 | 4.15E-01 | 0.94 (0.80 -1.09 ) |
|  | CD28+ CD45RA+ CD8dim T cell %CD8dim T cell | Inverse variance weighted | 5 | 4.16E-01 | 0.98 (0.94 -1.03 ) |
|  | CD86+ plasmacytoid Dendritic Cell %Dendritic Cell | Wald ratio | 1 | 4.17E-01 | 1.09 (0.88 -1.36 ) |
|  | CD62L- monocyte %monocyte | Inverse variance weighted | 3 | 4.19E-01 | 0.94 (0.82 -1.08 ) |
|  | CD25 on CD39+ CD4 regulatory T cell | Wald ratio | 1 | 4.19E-01 | 1.09 (0.88 -1.35 ) |
|  | CD25 on CD39+ activated CD4 regulatory T cell | Wald ratio | 1 | 4.19E-01 | 1.10 (0.88 -1.37 ) |
|  | T cell %lymphocyte | Inverse variance weighted | 2 | 4.22E-01 | 1.06 (0.92 -1.21 ) |
|  | CD28- CD8+ T cell %CD8+ T cell | Inverse variance weighted | 3 | 4.23E-01 | 1.07 (0.91 -1.26 ) |
|  | Effector Memory CD4+ T cell %T cell | Inverse variance weighted | 4 | 4.25E-01 | 0.92 (0.75 -1.13 ) |
|  | Unswitched memory B cell Absolute Count | Inverse variance weighted | 2 | 4.28E-01 | 1.11 (0.85 -1.45 ) |
|  | CD127 on CD45RA- CD4 not regulatory T cell | Wald ratio | 1 | 4.30E-01 | 0.91 (0.71 -1.16 ) |
|  | Natural Killer T Absolute Count | Inverse variance weighted | 5 | 4.32E-01 | 0.95 (0.83 -1.08 ) |
|  | CD19 on transitional B cell | Inverse variance weighted | 4 | 4.35E-01 | 0.96 (0.86 -1.07 ) |
|  | CD3 on CD28+ CD45RA+ CD8+ T cell | Inverse variance weighted | 3 | 4.38E-01 | 1.04 (0.95 -1.13 ) |
|  | CD45RA on Terminally Differentiated CD8+ T cell | Wald ratio | 1 | 4.39E-01 | 1.09 (0.87 -1.37 ) |
|  | CD28+ CD45RA- CD8dim T cell %CD8dim T cell | Inverse variance weighted | 2 | 4.42E-01 | 1.02 (0.97 -1.07 ) |
|  | CD39 on CD39+ CD8+ T cell | Inverse variance weighted | 2 | 4.45E-01 | 0.95 (0.82 -1.09 ) |
|  | SSC-A on HLA DR+ Natural Killer | Inverse variance weighted | 3 | 4.46E-01 | 1.04 (0.95 -1.13 ) |
|  | CCR2 on CD14+ CD16- monocyte | Inverse variance weighted | 2 | 4.47E-01 | 1.07 (0.90 -1.26 ) |
|  | CD19 on unswitched memory B cell | Inverse variance weighted | 4 | 4.49E-01 | 0.96 (0.86 -1.07 ) |
|  | CD25++ CD4+ T cell Absolute Count | Wald ratio | 1 | 4.49E-01 | 0.93 (0.76 -1.13 ) |
|  | CD25++ CD4+ T cell %CD4+ T cell | Wald ratio | 1 | 4.49E-01 | 0.94 (0.80 -1.10 ) |
|  | CD25++ CD4+ T cell %T cell | Wald ratio | 1 | 4.49E-01 | 0.93 (0.78 -1.12 ) |
|  | CD25 on CD4+ T cell | Wald ratio | 1 | 4.49E-01 | 0.91 (0.72 -1.16 ) |
|  | CD45RA+ CD8+ T cell Absolute Count | Inverse variance weighted | 3 | 4.49E-01 | 1.19 (0.76 -1.85 ) |
|  | Resting CD4 regulatory T cell Absolute Count | Inverse variance weighted | 4 | 4.51E-01 | 0.97 (0.91 -1.04 ) |
|  | Immature Myeloid-Derived Suppressor Cells %CD33dim HLA DR- CD66b- | Inverse variance weighted | 2 | 4.51E-01 | 1.05 (0.93 -1.17 ) |
|  | CD14+ CD16+ monocyte Absolute Count | Inverse variance weighted | 3 | 4.53E-01 | 1.03 (0.95 -1.12 ) |
|  | CD8dim Natural Killer T %lymphocyte | Inverse variance weighted | 6 | 4.53E-01 | 0.97 (0.88 -1.06 ) |
|  | CD14+ CD16+ monocyte %monocyte | Inverse variance weighted | 3 | 4.54E-01 | 1.03 (0.95 -1.11 ) |
|  | CD28- CD4-CD8- T cell Absolute Count | Inverse variance weighted | 2 | 4.56E-01 | 1.06 (0.91 -1.24 ) |
|  | Basophil %CD33dim HLA DR- CD66b- | Inverse variance weighted | 3 | 4.56E-01 | 0.97 (0.90 -1.05 ) |
|  | Dendritic Cell Absolute Count | Inverse variance weighted | 2 | 4.56E-01 | 0.96 (0.88 -1.06 ) |
|  | CD8 on naive CD8+ T cell | Wald ratio | 1 | 4.57E-01 | 0.96 (0.87 -1.06 ) |
|  | CD28- CD8dim T cell %T cell | Inverse variance weighted | 3 | 4.58E-01 | 0.95 (0.83 -1.09 ) |
|  | BAFF-R on IgD+ B cell | Inverse variance weighted | 10 | 4.59E-01 | 1.02 (0.97 -1.06 ) |
|  | IgD on IgD+ CD24+ B cell | Inverse variance weighted | 6 | 4.60E-01 | 0.97 (0.89 -1.05 ) |
|  | CD25 on IgD- CD24- B cell | Inverse variance weighted | 2 | 4.62E-01 | 0.92 (0.75 -1.14 ) |
|  | HLA DR+ CD8+ T cell Absolute Count | Inverse variance weighted | 3 | 4.63E-01 | 0.96 (0.86 -1.07 ) |
|  | BAFF-R on IgD+ CD24- B cell | Inverse variance weighted | 10 | 4.65E-01 | 1.02 (0.97 -1.06 ) |
|  | CD62L on monocyte | Inverse variance weighted | 2 | 4.66E-01 | 1.03 (0.95 -1.12 ) |
|  | IgD+ CD38- B cell Absolute Count | Wald ratio | 1 | 4.66E-01 | 1.28 (0.66 -2.49 ) |
|  | BAFF-R on B cell | Inverse variance weighted | 10 | 4.68E-01 | 1.02 (0.97 -1.06 ) |
|  | BAFF-R on IgD+ CD38+ B cell | Inverse variance weighted | 10 | 4.69E-01 | 1.02 (0.97 -1.06 ) |
|  | CD19 on IgD+ CD38+ B cell | Inverse variance weighted | 4 | 4.70E-01 | 0.96 (0.87 -1.06 ) |
|  | CD28 on CD39+ resting CD4 regulatory T cell | Inverse variance weighted | 2 | 4.72E-01 | 1.09 (0.86 -1.37 ) |
|  | Natural Killer T %lymphocyte | Inverse variance weighted | 7 | 4.73E-01 | 0.97 (0.88 -1.06 ) |
|  | HLA DR+ T cell%lymphocyte | Inverse variance weighted | 4 | 4.73E-01 | 0.97 (0.88 -1.06 ) |
|  | CD28+ CD45RA- CD8dim T cell %T cell | Inverse variance weighted | 3 | 4.73E-01 | 1.02 (0.97 -1.06 ) |
|  | CD127 on CD8+ T cell | Wald ratio | 1 | 4.74E-01 | 1.08 (0.88 -1.32 ) |
|  | CD19 on IgD+ CD38dim B cell | Inverse variance weighted | 4 | 4.74E-01 | 0.96 (0.85 -1.08 ) |
|  | CCR2 on monocyte | Inverse variance weighted | 2 | 4.85E-01 | 1.05 (0.92 -1.19 ) |
|  | CD3 on CD4+ T cell | Inverse variance weighted | 2 | 4.88E-01 | 1.07 (0.89 -1.28 ) |
|  | Plasma Blast-Plasma Cell %B cell | Wald ratio | 1 | 4.91E-01 | 0.87 (0.59 -1.29 ) |
|  | CX3CR1 on CD14- CD16- | Inverse variance weighted | 3 | 4.92E-01 | 1.04 (0.93 -1.18 ) |
|  | HLA DR on CD14- CD16- | Inverse variance weighted | 4 | 4.94E-01 | 0.97 (0.88 -1.06 ) |
|  | BAFF-R on IgD+ CD38dim B cell | Inverse variance weighted | 10 | 4.94E-01 | 1.02 (0.97 -1.06 ) |
|  | CD3 on T cell | Inverse variance weighted | 3 | 4.95E-01 | 0.97 (0.90 -1.05 ) |
|  | CD45RA+ CD8+ T cell %CD8+ T cell | Inverse variance weighted | 4 | 4.96E-01 | 1.01 (0.98 -1.04 ) |
|  | CD11c+ CD62L- monocyte Absolute Count | Wald ratio | 1 | 4.96E-01 | 1.12 (0.81 -1.55 ) |
|  | Activated & secreting CD4 regulatory T cell %CD4+ T cell | Inverse variance weighted | 9 | 4.98E-01 | 1.01 (0.98 -1.04 ) |
|  | HLA DR+ T cell Absolute Count | Inverse variance weighted | 3 | 5.02E-01 | 0.96 (0.87 -1.07 ) |
|  | BAFF-R on naive-mature B cell | Inverse variance weighted | 10 | 5.06E-01 | 1.01 (0.97 -1.06 ) |
|  | CD33 on basophil | Inverse variance weighted | 7 | 5.07E-01 | 0.99 (0.94 -1.03 ) |
|  | CD39+ CD4+ T cell Absolute Count | Inverse variance weighted | 5 | 5.07E-01 | 0.98 (0.91 -1.05 ) |
|  | CD8 on CD28+ CD45RA+ CD8+ T cell | Inverse variance weighted | 8 | 5.10E-01 | 1.03 (0.95 -1.12 ) |
|  | CD33 on Immature Myeloid-Derived Suppressor Cells | Inverse variance weighted | 6 | 5.13E-01 | 0.98 (0.94 -1.03 ) |
|  | CD4 on CD28+ CD4+ T cell | Inverse variance weighted | 2 | 5.15E-01 | 1.08 (0.86 -1.35 ) |
|  | CD25 on IgD- CD27- B cell | Inverse variance weighted | 2 | 5.17E-01 | 1.05 (0.90 -1.23 ) |
|  | FSC-A on CD8+ T cell | Wald ratio | 1 | 5.18E-01 | 1.17 (0.73 -1.88 ) |
|  | CD33- HLA DR- Absolute Count | Wald ratio | 1 | 5.22E-01 | 0.94 (0.77 -1.14 ) |
|  | CD25++ CD8+ T cell Absolute Count | Inverse variance weighted | 3 | 5.30E-01 | 1.03 (0.93 -1.14 ) |
|  | CD14+ CD16- monocyte %monocyte | Wald ratio | 1 | 5.31E-01 | 0.83 (0.46 -1.50 ) |
|  | CD16+ monocyte %monocyte | Wald ratio | 1 | 5.31E-01 | 1.21 (0.67 -2.18 ) |
|  | HLA DR++ monocyte %leukocyte | Inverse variance weighted | 2 | 5.32E-01 | 1.04 (0.92 -1.18 ) |
|  | CD19 on memory B cell | Inverse variance weighted | 3 | 5.32E-01 | 0.95 (0.82 -1.11 ) |
|  | Unswitched memory B cell %B cell | Wald ratio | 1 | 5.33E-01 | 1.09 (0.84 -1.41 ) |
|  | CD19 on CD24+ CD27+ B cell | Inverse variance weighted | 3 | 5.34E-01 | 0.96 (0.84 -1.10 ) |
|  | CD86 on monocyte | Wald ratio | 1 | 5.35E-01 | 0.93 (0.72 -1.18 ) |
|  | FSC-A on monocyte | Inverse variance weighted | 2 | 5.36E-01 | 0.87 (0.55 -1.36 ) |
|  | CD25++ CD8+ T cell %T cell | Inverse variance weighted | 2 | 5.44E-01 | 1.04 (0.92 -1.16 ) |
|  | Activated & resting CD4 regulatory T cell Absolute Count | Wald ratio | 1 | 5.45E-01 | 0.93 (0.73 -1.18 ) |
|  | Central Memory CD4+ T cell %CD4+ T cell | Wald ratio | 1 | 5.45E-01 | 1.07 (0.86 -1.32 ) |
|  | CD62L- Dendritic Cell %Dendritic Cell | Inverse variance weighted | 4 | 5.49E-01 | 0.98 (0.91 -1.05 ) |
|  | CD33dim HLA DR- Absolute Count | Inverse variance weighted | 3 | 5.49E-01 | 0.99 (0.95 -1.03 ) |
|  | IgD on IgD+ CD38- unswitched memory B cell | Inverse variance weighted | 2 | 5.50E-01 | 0.98 (0.92 -1.04 ) |
|  | CD28+ CD45RA- CD8+ T cell Absolute Count | Inverse variance weighted | 2 | 5.50E-01 | 1.02 (0.95 -1.11 ) |
|  | Basophil Absolute Count | Inverse variance weighted | 3 | 5.51E-01 | 0.99 (0.95 -1.03 ) |
|  | IgD- CD38+ B cell %B cell | Wald ratio | 1 | 5.53E-01 | 0.92 (0.71 -1.20 ) |
|  | CD8 on HLA DR+ CD8+ T cell | Inverse variance weighted | 2 | 5.55E-01 | 1.05 (0.90 -1.22 ) |
|  | Naive-mature B cell %B cell | Wald ratio | 1 | 5.57E-01 | 1.10 (0.81 -1.48 ) |
|  | CD20 on B cell | Inverse variance weighted | 4 | 5.57E-01 | 1.06 (0.87 -1.31 ) |
|  | CD20 on IgD+ CD38- B cell | Inverse variance weighted | 4 | 5.59E-01 | 0.96 (0.84 -1.10 ) |
|  | CD20 on switched memory B cell | Inverse variance weighted | 7 | 5.60E-01 | 0.97 (0.89 -1.06 ) |
|  | IgD on unswitched memory B cell | Inverse variance weighted | 5 | 5.66E-01 | 0.98 (0.93 -1.04 ) |
|  | PDL-1 on CD14- CD16+ monocyte | Inverse variance weighted | 2 | 5.67E-01 | 1.03 (0.93 -1.14 ) |
|  | CD14+ CD16- monocyte Absolute Count | Inverse variance weighted | 3 | 5.69E-01 | 0.95 (0.81 -1.12 ) |
|  | Naive CD8+ T cell %T cell | Inverse variance weighted | 3 | 5.69E-01 | 1.00 (0.98 -1.01 ) |
|  | CD62L- plasmacytoid Dendritic Cell %Dendritic Cell | Inverse variance weighted | 2 | 5.71E-01 | 0.96 (0.82 -1.12 ) |
|  | CD20 on unswitched memory B cell | Inverse variance weighted | 4 | 5.76E-01 | 0.95 (0.79 -1.14 ) |
|  | Memory B cell Absolute Count | Wald ratio | 1 | 5.82E-01 | 1.18 (0.66 -2.10 ) |
|  | IgD+ CD24+ B cell Absolute Count | Wald ratio | 1 | 5.82E-01 | 1.15 (0.69 -1.92 ) |
|  | CD24+ CD27+ B cell Absolute Count | Wald ratio | 1 | 5.82E-01 | 1.18 (0.65 -2.14 ) |
|  | FSC-A on plasmacytoid Dendritic Cell | Inverse variance weighted | 4 | 5.85E-01 | 1.04 (0.90 -1.20 ) |
|  | Activated & resting CD4 regulatory T cell %CD4 regulatory T cell | Inverse variance weighted | 5 | 5.86E-01 | 0.99 (0.94 -1.04 ) |
|  | Secreting CD4 regulatory T cell %CD4 regulatory T cell | Inverse variance weighted | 5 | 5.87E-01 | 1.01 (0.97 -1.06 ) |
|  | Effector Memory CD4-CD8- T cell Absolute Count | Inverse variance weighted | 4 | 5.88E-01 | 1.03 (0.92 -1.15 ) |
|  | CD25 on IgD+ B cell | Inverse variance weighted | 2 | 5.89E-01 | 0.96 (0.85 -1.10 ) |
|  | CD39+ activated CD4 regulatory T cell Absolute Count | Inverse variance weighted | 4 | 5.91E-01 | 0.98 (0.90 -1.06 ) |
|  | CD27 on unswitched memory B cell | Inverse variance weighted | 11 | 5.96E-01 | 0.98 (0.91 -1.06 ) |
|  | CD64 on CD14- CD16+ monocyte | Wald ratio | 1 | 5.97E-01 | 0.93 (0.70 -1.22 ) |
|  | CD20 on IgD- CD38dim B cell | Inverse variance weighted | 7 | 5.99E-01 | 0.98 (0.89 -1.07 ) |
|  | CD38 on IgD+ CD38dim B cell | Inverse variance weighted | 4 | 6.08E-01 | 1.03 (0.93 -1.13 ) |
|  | CD19 on IgD+ CD24- B cell | Inverse variance weighted | 4 | 6.08E-01 | 0.97 (0.86 -1.09 ) |
|  | CD45RA- CD28- CD8+ T cell %T cell | Inverse variance weighted | 40 | 6.09E-01 | 1.00 (1.00 -1.00 ) |
|  | Effector Memory CD8+ T cell %T cell | Inverse variance weighted | 2 | 6.10E-01 | 1.02 (0.93 -1.12 ) |
|  | CD8 on CD28+ CD45RA- CD8+ T cell | Inverse variance weighted | 2 | 6.18E-01 | 0.95 (0.78 -1.16 ) |
|  | Activated & resting CD4 regulatory T cell %CD4+ T cell | Inverse variance weighted | 3 | 6.28E-01 | 0.98 (0.92 -1.05 ) |
|  | CD25 on switched memory B cell | Inverse variance weighted | 3 | 6.30E-01 | 0.98 (0.88 -1.08 ) |
|  | Terminally Differentiated CD4+ T cell %T cell | Inverse variance weighted | 5 | 6.31E-01 | 0.96 (0.82 -1.13 ) |
|  | Activated & secreting CD4 regulatory T cell %CD4 regulatory T cell | Inverse variance weighted | 8 | 6.35E-01 | 1.01 (0.96 -1.06 ) |
|  | CD45RA+ CD8+ T cell %T cell | Inverse variance weighted | 3 | 6.36E-01 | 1.08 (0.78 -1.51 ) |
|  | CD127 on CD45RA+ CD4+ T cell | Inverse variance weighted | 4 | 6.36E-01 | 1.06 (0.84 -1.32 ) |
|  | CD19 on CD20- B cell | Wald ratio | 1 | 6.38E-01 | 1.03 (0.91 -1.17 ) |
|  | HLA DR++ monocyte %monocyte | Inverse variance weighted | 4 | 6.38E-01 | 1.02 (0.93 -1.12 ) |
|  | CD25 on transitional B cell | Inverse variance weighted | 2 | 6.39E-01 | 0.96 (0.83 -1.12 ) |
|  | HLA DR+ CD4+ T cell %lymphocyte | Inverse variance weighted | 2 | 6.41E-01 | 0.95 (0.78 -1.16 ) |
|  | SSC-A on CD4+ T cell | Inverse variance weighted | 4 | 6.42E-01 | 0.93 (0.69 -1.26 ) |
|  | CD127 on CD28+ CD45RA- CD8+ T cell | Wald ratio | 1 | 6.43E-01 | 1.02 (0.93 -1.12 ) |
|  | CD45RA+ CD28- CD8+ T cell %T cell | Inverse variance weighted | 63 | 6.43E-01 | 1.00 (1.00 -1.00 ) |
|  | Natural Killer %lymphocyte | Inverse variance weighted | 3 | 6.46E-01 | 0.97 (0.86 -1.10 ) |
|  | Effector Memory CD8+ T cell Absolute Count | Inverse variance weighted | 2 | 6.48E-01 | 1.05 (0.85 -1.30 ) |
|  | CD3- lymphocyte Absolute Count | Wald ratio | 1 | 6.50E-01 | 0.96 (0.80 -1.15 ) |
|  | CD8dim T cell %T cell | Inverse variance weighted | 2 | 6.53E-01 | 0.94 (0.74 -1.21 ) |
|  | CD4 on CD39+ secreting CD4 regulatory T cell | Inverse variance weighted | 3 | 6.53E-01 | 1.06 (0.83 -1.35 ) |
|  | CD80 on myeloid Dendritic Cell | Inverse variance weighted | 4 | 6.54E-01 | 0.97 (0.83 -1.12 ) |
|  | CD3 on CD4 regulatory T cell | Inverse variance weighted | 2 | 6.63E-01 | 0.97 (0.87 -1.09 ) |
|  | CD8 on Natural Killer T | Inverse variance weighted | 2 | 6.63E-01 | 1.11 (0.69 -1.79 ) |
|  | CD20 on naive-mature B cell | Inverse variance weighted | 6 | 6.65E-01 | 1.02 (0.93 -1.12 ) |
|  | CD25 on IgD+ CD38dim B cell | Inverse variance weighted | 2 | 6.68E-01 | 0.97 (0.83 -1.13 ) |
|  | CD20 on IgD- CD24- B cell | Inverse variance weighted | 2 | 6.70E-01 | 1.06 (0.81 -1.40 ) |
|  | CD8dim T cell %leukocyte | Inverse variance weighted | 2 | 6.71E-01 | 0.94 (0.73 -1.23 ) |
|  | IgD on IgD+ CD24- B cell | Inverse variance weighted | 5 | 6.73E-01 | 0.99 (0.93 -1.05 ) |
|  | IgD+ CD24- B cell %B cell | Wald ratio | 1 | 6.74E-01 | 1.03 (0.89 -1.19 ) |
|  | CD45RA on CD39+ resting CD4 regulatory T cell | Wald ratio | 1 | 6.75E-01 | 1.02 (0.94 -1.10 ) |
|  | CD19 on naive-mature B cell | Inverse variance weighted | 2 | 6.75E-01 | 1.08 (0.76 -1.54 ) |
|  | HVEM on T cell | Wald ratio | 1 | 6.76E-01 | 1.03 (0.90 -1.17 ) |
|  | HVEM on Effector Memory CD8+ T cell | Wald ratio | 1 | 6.76E-01 | 1.03 (0.90 -1.17 ) |
|  | HVEM on CD4+ T cell | Wald ratio | 1 | 6.76E-01 | 1.03 (0.91 -1.16 ) |
|  | HVEM on Central Memory CD4+ T cell | Wald ratio | 1 | 6.76E-01 | 1.03 (0.90 -1.17 ) |
|  | HVEM on CD45RA- CD4+ T cell | Wald ratio | 1 | 6.76E-01 | 1.03 (0.91 -1.16 ) |
|  | HVEM on Central Memory CD8+ T cell | Wald ratio | 1 | 6.76E-01 | 1.03 (0.91 -1.16 ) |
|  | IgD on IgD+ CD38dim B cell | Inverse variance weighted | 5 | 6.76E-01 | 0.99 (0.93 -1.05 ) |
|  | CD4 on CD4+ T cell | Wald ratio | 1 | 6.78E-01 | 0.97 (0.84 -1.12 ) |
|  | CD4 on CD4 regulatory T cell | Wald ratio | 1 | 6.78E-01 | 0.96 (0.82 -1.14 ) |
|  | CD4 on resting CD4 regulatory T cell | Wald ratio | 1 | 6.78E-01 | 0.95 (0.77 -1.19 ) |
|  | CD4 on activated CD4 regulatory T cell | Wald ratio | 1 | 6.78E-01 | 0.97 (0.82 -1.14 ) |
|  | CD4 on secreting CD4 regulatory T cell | Wald ratio | 1 | 6.78E-01 | 0.96 (0.79 -1.17 ) |
|  | CD4 on activated & secreting CD4 regulatory T cell | Wald ratio | 1 | 6.78E-01 | 0.96 (0.81 -1.15 ) |
|  | Naive CD8+ T cell Absolute Count | Wald ratio | 1 | 6.79E-01 | 0.91 (0.58 -1.43 ) |
|  | CD3 on secreting CD4 regulatory T cell | Inverse variance weighted | 2 | 6.80E-01 | 0.98 (0.87 -1.10 ) |
|  | CD28- CD8dim T cell Absolute Count | Inverse variance weighted | 2 | 6.82E-01 | 1.04 (0.86 -1.27 ) |
|  | CD25++ CD45RA- CD4 not regulatory T cell Absolute Count | Inverse variance weighted | 2 | 6.83E-01 | 1.05 (0.84 -1.29 ) |
|  | CD127 on granulocyte | Inverse variance weighted | 8 | 6.86E-01 | 1.01 (0.96 -1.07 ) |
|  | SSC-A on Natural Killer T | Inverse variance weighted | 2 | 6.92E-01 | 0.96 (0.78 -1.18 ) |
|  | CD25 on CD45RA- CD4 not regulatory T cell | Inverse variance weighted | 2 | 6.92E-01 | 0.97 (0.84 -1.13 ) |
|  | CD25 on IgD- CD38- B cell | Inverse variance weighted | 3 | 6.96E-01 | 1.02 (0.91 -1.15 ) |
|  | CD45 on Natural Killer | Inverse variance weighted | 2 | 6.96E-01 | 1.06 (0.79 -1.42 ) |
|  | IgD on IgD+ CD38+ B cell | Inverse variance weighted | 6 | 6.97E-01 | 0.99 (0.95 -1.04 ) |
|  | HVEM on naive CD8+ T cell | Inverse variance weighted | 2 | 7.03E-01 | 0.98 (0.86 -1.10 ) |
|  | CD127 on CD28+ CD4+ T cell | Inverse variance weighted | 3 | 7.04E-01 | 1.03 (0.90 -1.16 ) |
|  | Transitional B cell Absolute Count | Wald ratio | 1 | 7.12E-01 | 1.06 (0.78 -1.43 ) |
|  | Naive CD4+ T cell Absolute Count | Inverse variance weighted | 2 | 7.15E-01 | 1.10 (0.66 -1.82 ) |
|  | FSC-A on granulocyte | Inverse variance weighted | 2 | 7.18E-01 | 0.97 (0.85 -1.12 ) |
|  | Hematopoietic Stem Cell Absolute Count | Inverse variance weighted | 2 | 7.19E-01 | 0.99 (0.93 -1.05 ) |
|  | CD3 on naive CD8+ T cell | Inverse variance weighted | 4 | 7.20E-01 | 0.99 (0.92 -1.06 ) |
|  | CCR2 on granulocyte | Inverse variance weighted | 2 | 7.21E-01 | 0.97 (0.81 -1.15 ) |
|  | CD4-CD8- T cell %leukocyte | Inverse variance weighted | 4 | 7.23E-01 | 1.03 (0.89 -1.18 ) |
|  | CD45 on CD33+ HLA DR+ CD14- | Wald ratio | 1 | 7.24E-01 | 0.97 (0.80 -1.17 ) |
|  | HLA DR on CD14+ CD16- monocyte | Inverse variance weighted | 5 | 7.24E-01 | 1.01 (0.94 -1.09 ) |
|  | CD20- CD38- B cell %B cell | Wald ratio | 1 | 7.25E-01 | 1.03 (0.88 -1.19 ) |
|  | CD25 on naive-mature B cell | Inverse variance weighted | 4 | 7.31E-01 | 1.02 (0.90 -1.17 ) |
|  | HLA DR on CD14+ monocyte | Inverse variance weighted | 5 | 7.32E-01 | 1.01 (0.94 -1.10 ) |
|  | CD19 on switched memory B cell | Inverse variance weighted | 2 | 7.34E-01 | 0.90 (0.50 -1.63 ) |
|  | IgD+ B cell %B cell | Wald ratio | 1 | 7.38E-01 | 0.96 (0.73 -1.24 ) |
|  | CD39 on granulocyte | Inverse variance weighted | 2 | 7.39E-01 | 0.96 (0.78 -1.19 ) |
|  | SSC-A on myeloid Dendritic Cell | Inverse variance weighted | 5 | 7.41E-01 | 0.97 (0.79 -1.18 ) |
|  | Terminally Differentiated CD4-CD8- T cell %T cell | Inverse variance weighted | 3 | 7.43E-01 | 0.97 (0.80 -1.17 ) |
|  | CD45 on CD33- HLA DR+ | Inverse variance weighted | 2 | 7.45E-01 | 1.03 (0.84 -1.27 ) |
|  | FSC-A on Natural Killer | Inverse variance weighted | 3 | 7.55E-01 | 1.02 (0.90 -1.16 ) |
|  | Lymphocyte %leukocyte | Wald ratio | 1 | 7.60E-01 | 1.06 (0.73 -1.54 ) |
|  | Granulocyte %leukocyte | Wald ratio | 1 | 7.60E-01 | 0.94 (0.65 -1.37 ) |
|  | CD8 on Terminally Differentiated CD8+ T cell | Inverse variance weighted | 2 | 7.60E-01 | 0.97 (0.81 -1.16 ) |
|  | CD20 on IgD+ CD38dim B cell | Inverse variance weighted | 7 | 7.62E-01 | 1.02 (0.91 -1.13 ) |
|  | Natural Killer Absolute Count | Inverse variance weighted | 3 | 7.63E-01 | 0.98 (0.84 -1.13 ) |
|  | Terminally Differentiated CD4-CD8- T cell Absolute Count | Wald ratio | 1 | 7.64E-01 | 0.89 (0.42 -1.88 ) |
|  | CD14- CD16+ monocyte %monocyte | Wald ratio | 1 | 7.66E-01 | 1.04 (0.81 -1.34 ) |
|  | HLA DR+ CD4+ T cell Absolute Count | Inverse variance weighted | 2 | 7.72E-01 | 0.97 (0.77 -1.22 ) |
|  | IgD- CD27- B cell %B cell | Inverse variance weighted | 3 | 7.75E-01 | 1.02 (0.91 -1.13 ) |
|  | CD38 on IgD+ CD24- B cell | Inverse variance weighted | 2 | 7.76E-01 | 1.03 (0.84 -1.26 ) |
|  | CD38 on CD3- CD19- | Wald ratio | 1 | 7.79E-01 | 0.97 (0.80 -1.18 ) |
|  | Plasmacytoid Dendritic Cell %Dendritic Cell | Inverse variance weighted | 2 | 7.84E-01 | 1.02 (0.89 -1.16 ) |
|  | CD27 on IgD+ CD24+ B cell | Inverse variance weighted | 6 | 7.85E-01 | 0.99 (0.92 -1.06 ) |
|  | CD16-CD56 on Natural Killer | Inverse variance weighted | 8 | 7.88E-01 | 1.01 (0.93 -1.10 ) |
|  | CD45RA- CD28- CD8+ T cell %CD8+ T cell | Inverse variance weighted | 10 | 7.88E-01 | 1.00 (1.00 -1.00 ) |
|  | CD45RA- CD28- CD8+ T cell Absolute Count | Inverse variance weighted | 138 | 7.92E-01 | 1.00 (1.00 -1.00 ) |
|  | CD86 on granulocyte | Inverse variance weighted | 2 | 7.94E-01 | 0.96 (0.73 -1.28 ) |
|  | CD25 on IgD- CD38dim B cell | Inverse variance weighted | 3 | 7.95E-01 | 0.98 (0.87 -1.11 ) |
|  | FSC-A on CD14+ monocyte | Wald ratio | 1 | 7.97E-01 | 1.08 (0.62 -1.87 ) |
|  | IgD on IgD+ CD38- B cell | Wald ratio | 1 | 7.97E-01 | 0.99 (0.92 -1.07 ) |
|  | CD25++ CD45RA- CD4 not regulatory T cell %T cell | Inverse variance weighted | 3 | 7.98E-01 | 1.01 (0.95 -1.07 ) |
|  | CD33+ HLA DR+ CD14dim Absolute Count | Inverse variance weighted | 2 | 7.98E-01 | 0.97 (0.76 -1.23 ) |
|  | HVEM on naive CD4+ T cell | Wald ratio | 1 | 8.01E-01 | 1.02 (0.88 -1.18 ) |
|  | CD25 on CD45RA+ CD4 not regulatory T cell | Wald ratio | 1 | 8.06E-01 | 1.02 (0.86 -1.21 ) |
|  | Terminally Differentiated CD8+ T cell Absolute Count | Wald ratio | 1 | 8.07E-01 | 1.04 (0.77 -1.40 ) |
|  | CD27 on IgD- CD38+ B cell | Inverse variance weighted | 2 | 8.10E-01 | 1.04 (0.78 -1.37 ) |
|  | CD64 on CD14+ CD16- monocyte | Inverse variance weighted | 8 | 8.10E-01 | 1.01 (0.96 -1.06 ) |
|  | CCR2 on myeloid Dendritic Cell | Inverse variance weighted | 2 | 8.11E-01 | 0.99 (0.88 -1.11 ) |
|  | Naive-mature B cell %lymphocyte | Wald ratio | 1 | 8.17E-01 | 1.01 (0.92 -1.12 ) |
|  | IgD+ CD24- B cell %lymphocyte | Wald ratio | 1 | 8.17E-01 | 1.01 (0.91 -1.12 ) |
|  | Plasmacytoid Dendritic Cell Absolute Count | Inverse variance weighted | 2 | 8.20E-01 | 1.04 (0.76 -1.42 ) |
|  | CD64 on monocyte | Inverse variance weighted | 8 | 8.22E-01 | 1.01 (0.95 -1.06 ) |
|  | CD62L- monocyte Absolute Count | Inverse variance weighted | 2 | 8.25E-01 | 1.03 (0.78 -1.36 ) |
|  | CD8+ Natural Killer T %lymphocyte | Wald ratio | 1 | 8.27E-01 | 0.96 (0.64 -1.42 ) |
|  | CD38 on naive-mature B cell | Inverse variance weighted | 3 | 8.27E-01 | 1.01 (0.90 -1.15 ) |
|  | CD8dim Natural Killer T Absolute Count | Inverse variance weighted | 5 | 8.29E-01 | 0.99 (0.89 -1.09 ) |
|  | CD28 on CD45RA- CD4 not regulatory T cell | Inverse variance weighted | 3 | 8.37E-01 | 1.01 (0.91 -1.12 ) |
|  | CD14 on CD14+ CD16- monocyte | Wald ratio | 1 | 8.40E-01 | 0.98 (0.84 -1.15 ) |
|  | CD45 on CD8+ T cell | Inverse variance weighted | 2 | 8.43E-01 | 1.01 (0.91 -1.13 ) |
|  | CD19 on IgD+ B cell | Inverse variance weighted | 2 | 8.44E-01 | 1.05 (0.65 -1.69 ) |
|  | CD27 on memory B cell | Inverse variance weighted | 7 | 8.48E-01 | 0.99 (0.92 -1.07 ) |
|  | CD24 on IgD+ CD38+ B cell | Inverse variance weighted | 2 | 8.49E-01 | 0.97 (0.73 -1.30 ) |
|  | CD25 on resting CD4 regulatory T cell | Wald ratio | 1 | 8.50E-01 | 1.10 (0.41 -2.97 ) |
|  | CD8dim T cell Absolute Count | Inverse variance weighted | 3 | 8.52E-01 | 0.98 (0.81 -1.19 ) |
|  | CD33dim HLA DR+ CD11b- Absolute Count | Inverse variance weighted | 2 | 8.52E-01 | 1.01 (0.89 -1.15 ) |
|  | PDL-1 on monocyte | Inverse variance weighted | 2 | 8.55E-01 | 1.01 (0.90 -1.13 ) |
|  | CD38 on transitional B cell | Inverse variance weighted | 5 | 8.55E-01 | 1.01 (0.92 -1.10 ) |
|  | FSC-A on Natural Killer T | Inverse variance weighted | 2 | 8.57E-01 | 0.98 (0.78 -1.23 ) |
|  | CD14 on CD14+ CD16+ monocyte | Inverse variance weighted | 2 | 8.59E-01 | 1.02 (0.86 -1.21 ) |
|  | HVEM on Terminally Differentiated CD4+ T cell | Wald ratio | 1 | 8.60E-01 | 1.01 (0.87 -1.18 ) |
|  | HVEM on CD8+ T cell | Wald ratio | 1 | 8.60E-01 | 1.01 (0.88 -1.17 ) |
|  | CD38 on IgD- CD38dim B cell | Inverse variance weighted | 3 | 8.62E-01 | 1.01 (0.90 -1.13 ) |
|  | CD25++ CD45RA- CD4 not regulatory T cell %CD4+ T cell | Inverse variance weighted | 3 | 8.64E-01 | 0.99 (0.93 -1.06 ) |
|  | CD127 on CD28+ CD45RA+ CD8+ T cell | Inverse variance weighted | 2 | 8.64E-01 | 1.02 (0.83 -1.26 ) |
|  | CD28- CD4-CD8- T cell %T cell | Inverse variance weighted | 3 | 8.65E-01 | 1.01 (0.86 -1.20 ) |
|  | CD16-CD56 on Natural Killer T | Inverse variance weighted | 4 | 8.67E-01 | 1.01 (0.92 -1.10 ) |
|  | CD25 on IgD+ CD38+ B cell | Inverse variance weighted | 3 | 8.77E-01 | 1.01 (0.90 -1.14 ) |
|  | CD8+ Natural Killer T %T cell | Wald ratio | 1 | 8.77E-01 | 0.97 (0.63 -1.48 ) |
|  | Effector Memory CD4+ T cell %CD4+ T cell | Inverse variance weighted | 2 | 8.79E-01 | 1.02 (0.80 -1.29 ) |
|  | CD4-CD8- T cell %T cell | Inverse variance weighted | 3 | 8.81E-01 | 1.01 (0.85 -1.21 ) |
|  | CD4 on Effector Memory CD4+ T cell | Inverse variance weighted | 3 | 8.88E-01 | 0.99 (0.91 -1.09 ) |
|  | CCR2 on monocyte | Wald ratio | 1 | 8.91E-01 | 0.99 (0.83 -1.17 ) |
|  | HLA DR on monocyte | Inverse variance weighted | 4 | 8.92E-01 | 1.01 (0.92 -1.10 ) |
|  | CD25 on CD39+ resting CD4 regulatory T cell | Wald ratio | 1 | 8.92E-01 | 1.02 (0.74 -1.41 ) |
|  | FSC-A on myeloid Dendritic Cell | Inverse variance weighted | 3 | 8.94E-01 | 0.99 (0.90 -1.09 ) |
|  | CD24+ CD27+ B cell %B cell | Inverse variance weighted | 3 | 8.94E-01 | 0.99 (0.88 -1.12 ) |
|  | CD11b on CD33dim HLA DR- | Wald ratio | 1 | 8.95E-01 | 0.99 (0.89 -1.10 ) |
|  | CD11b on basophil | Wald ratio | 1 | 8.95E-01 | 0.99 (0.90 -1.10 ) |
|  | CD4-CD8- Natural Killer T %lymphocyte | Inverse variance weighted | 6 | 8.97E-01 | 1.01 (0.92 -1.10 ) |
|  | Unswitched memory B cell %lymphocyte | Inverse variance weighted | 4 | 8.97E-01 | 1.01 (0.85 -1.20 ) |
|  | CD28+ CD45RA+ CD8+ T cell %CD8+ T cell | Wald ratio | 1 | 9.03E-01 | 0.99 (0.87 -1.13 ) |
|  | CD11c on CD62L+ myeloid Dendritic Cell | Wald ratio | 1 | 9.04E-01 | 1.01 (0.91 -1.12 ) |
|  | CD3 on Natural Killer T | Inverse variance weighted | 2 | 9.05E-01 | 1.01 (0.83 -1.23 ) |
|  | CD25 on IgD+ CD38- naive B cell | Inverse variance weighted | 3 | 9.06E-01 | 1.01 (0.91 -1.12 ) |
|  | Monocyte Absolute Count | Wald ratio | 1 | 9.06E-01 | 0.98 (0.75 -1.29 ) |
|  | CCR2 on CD62L+ myeloid Dendritic Cell | Inverse variance weighted | 3 | 9.09E-01 | 1.00 (0.91 -1.08 ) |
|  | CD20 on IgD+ B cell | Inverse variance weighted | 5 | 9.11E-01 | 1.01 (0.91 -1.11 ) |
|  | HVEM on Effector Memory CD4+ T cell | Wald ratio | 1 | 9.13E-01 | 1.01 (0.89 -1.14 ) |
|  | CD80 on CD62L+ myeloid Dendritic Cell | Inverse variance weighted | 3 | 9.15E-01 | 0.99 (0.81 -1.21 ) |
|  | CD86 on CD62L+ myeloid Dendritic Cell | Wald ratio | 1 | 9.16E-01 | 1.01 (0.91 -1.11 ) |
|  | CD3- lymphocyte %leukocyte | Wald ratio | 1 | 9.21E-01 | 1.01 (0.76 -1.35 ) |
|  | CD28- CD4-CD8- T cell %CD4-CD8- T cell | Wald ratio | 1 | 9.21E-01 | 0.99 (0.76 -1.28 ) |
|  | CD28+ CD4-CD8- T cell %CD4-CD8- T cell | Wald ratio | 1 | 9.21E-01 | 1.01 (0.78 -1.31 ) |
|  | CD28- CD8dim T cell %CD8dim T cell | Wald ratio | 1 | 9.21E-01 | 0.99 (0.75 -1.30 ) |
|  | Effector Memory CD8+ T cell %CD8+ T cell | Inverse variance weighted | 3 | 9.25E-01 | 1.00 (0.93 -1.09 ) |
|  | SSC-A on Natural Killer | Wald ratio | 1 | 9.30E-01 | 0.99 (0.84 -1.17 ) |
|  | CD19 on CD20- CD38- B cell | Wald ratio | 1 | 9.32E-01 | 0.99 (0.71 -1.37 ) |
|  | CD38 on IgD+ CD38+ B cell | Inverse variance weighted | 3 | 9.34E-01 | 1.01 (0.86 -1.18 ) |
|  | CD25 on IgD+ CD24- B cell | Inverse variance weighted | 3 | 9.35E-01 | 1.00 (0.90 -1.13 ) |
|  | CD14- CD16+ monocyte Absolute Count | Wald ratio | 1 | 9.44E-01 | 0.99 (0.84 -1.17 ) |
|  | CCR2 on CD14- CD16+ monocyte | Wald ratio | 1 | 9.44E-01 | 1.01 (0.76 -1.34 ) |
|  | CD3 on CD45RA+ CD4+ T cell | Inverse variance weighted | 4 | 9.44E-01 | 1.00 (0.92 -1.08 ) |
|  | CD4 on CD39+ resting CD4 regulatory T cell | Wald ratio | 1 | 9.47E-01 | 0.99 (0.78 -1.26 ) |
|  | CD27 on IgD+ CD38- unswitched memory B cell | Inverse variance weighted | 5 | 9.54E-01 | 1.00 (0.94 -1.07 ) |
|  | CD25 on IgD+ CD38- B cell | Inverse variance weighted | 3 | 9.54E-01 | 1.00 (0.86 -1.15 ) |
|  | CD4-CD8- Natural Killer T %T cell | Inverse variance weighted | 5 | 9.55E-01 | 1.00 (0.91 -1.10 ) |
|  | CD28+ CD45RA- CD8+ T cell %CD8+ T cell | Inverse variance weighted | 3 | 9.57E-01 | 1.00 (0.88 -1.13 ) |
|  | IgD+ CD38+ B cell Absolute Count | Inverse variance weighted | 2 | 9.57E-01 | 1.01 (0.72 -1.41 ) |
|  | CD19 on IgD+ CD38- unswitched memory B cell | Inverse variance weighted | 3 | 9.58E-01 | 0.99 (0.80 -1.24 ) |
|  | IgD on transitional B cell | Inverse variance weighted | 5 | 9.59E-01 | 1.00 (0.94 -1.06 ) |
|  | CD8 on CD8+ T cell | Wald ratio | 1 | 9.61E-01 | 1.00 (0.89 -1.13 ) |
|  | CD4 on CD39+ activated CD4 regulatory T cell | Inverse variance weighted | 3 | 9.63E-01 | 1.00 (0.86 -1.16 ) |
|  | CD39+ resting CD4 regulatory T cell %resting CD4 regulatory T cell | Inverse variance weighted | 5 | 9.63E-01 | 1.00 (0.92 -1.08 ) |
|  | CD28+ CD45RA- CD8+ T cell %T cell | Inverse variance weighted | 3 | 9.64E-01 | 1.00 (0.94 -1.06 ) |
|  | CD8 on CD28- CD8+ T cell | Inverse variance weighted | 2 | 9.65E-01 | 0.99 (0.72 -1.37 ) |
|  | IgD on IgD+ B cell | Inverse variance weighted | 2 | 9.66E-01 | 1.00 (0.94 -1.07 ) |
|  | CD20 on IgD+ CD38+ B cell | Inverse variance weighted | 5 | 9.73E-01 | 1.00 (0.91 -1.10 ) |
|  | CD62L on CD62L+ Dendritic Cell | Inverse variance weighted | 2 | 9.74E-01 | 1.00 (0.86 -1.16 ) |
|  | Effector Memory CD4-CD8- T cell %CD4-CD8- T cell | Inverse variance weighted | 4 | 9.74E-01 | 1.00 (0.84 -1.20 ) |
|  | CD45 on Immature Myeloid-Derived Suppressor Cells | Inverse variance weighted | 2 | 9.78E-01 | 1.00 (0.92 -1.08 ) |
|  | CD20 on IgD+ CD24- B cell | Inverse variance weighted | 4 | 9.80E-01 | 1.00 (0.89 -1.11 ) |
|  | IgD- CD27- B cell Absolute Count | Inverse variance weighted | 2 | 9.84E-01 | 1.00 (0.83 -1.20 ) |
|  | HLA DR+ T cell%T cell | Inverse variance weighted | 4 | 9.86E-01 | 1.00 (0.88 -1.14 ) |
|  | B cell Absolute Count | Wald ratio | 1 | 9.87E-01 | 1.00 (0.74 -1.34 ) |
|  | CD20 on IgD- CD38- B cell | Inverse variance weighted | 4 | 9.88E-01 | 1.00 (0.87 -1.15 ) |
|  | Effector Memory CD4-CD8- T cell %T cell | Inverse variance weighted | 3 | 9.92E-01 | 1.00 (0.86 -1.17 ) |
|  | CD39+ CD8+ T cell %CD8+ T cell | Inverse variance weighted | 6 | 9.96E-01 | 1.00 (0.93 -1.08 ) |
|  | CD8 on Central Memory CD8+ T cell | Inverse variance weighted | 2 | 9.99E-01 | 1.00 (0.89 -1.12 ) |
|  | CD25 on B cell | Inverse variance weighted | 3 | 9.99E-01 | 1.00 (0.90 -1.12 ) |

**Table S5. Two-sample Mendelian randomization estimations showing the effects of Immune cell traits on the risk of eclampsia.**

| outcome | exposure | method | nsnp | pval | or |
| --- | --- | --- | --- | --- | --- |
| eclampsia | CD25++ CD8+ T cell Absolute Count | Inverse variance weighted | 3 | 5.75E-04 | 2.02 (1.35 -3.02 ) |
|  | IgD on IgD+ CD24+ B cell | Inverse variance weighted | 6 | 1.39E-03 | 1.40 (1.14 -1.71 ) |
|  | CD127 on CD8+ T cell | Wald ratio | 1 | 1.92E-03 | 3.13 (1.52 -6.45 ) |
|  | CD25++ CD8+ T cell %CD8+ T cell | Wald ratio | 1 | 2.35E-03 | 2.09 (1.30 -3.37 ) |
|  | IgD on IgD+ CD38- unswitched memory B cell | Inverse variance weighted | 2 | 4.19E-03 | 1.33 (1.09 -1.61 ) |
|  | CD28- CD25++ CD8+ T cell Absolute Count | Wald ratio | 1 | 4.33E-03 | 3.72 (1.51 -9.19 ) |
|  | IgD on IgD+ B cell | Inverse variance weighted | 2 | 4.42E-03 | 1.39 (1.11 -1.74 ) |
|  | CD38 on CD3- CD19- | Wald ratio | 1 | 4.43E-03 | 0.37 (0.19 -0.73 ) |
|  | IgD on IgD+ CD38- B cell | Wald ratio | 1 | 5.37E-03 | 1.48 (1.12 -1.96 ) |
|  | CD80 on monocyte | Inverse variance weighted | 6 | 5.71E-03 | 1.36 (1.09 -1.70 ) |
|  | CD24+ CD27+ B cell %lymphocyte | Inverse variance weighted | 3 | 7.19E-03 | 0.40 (0.20 -0.78 ) |
|  | CD80 on CD62L+ plasmacytoid Dendritic Cell | Inverse variance weighted | 2 | 7.35E-03 | 0.53 (0.33 -0.84 ) |
|  | CD80 on plasmacytoid Dendritic Cell | Inverse variance weighted | 2 | 7.41E-03 | 0.53 (0.33 -0.84 ) |
|  | CD123 on CD62L+ plasmacytoid Dendritic Cell | Inverse variance weighted | 2 | 7.48E-03 | 0.55 (0.35 -0.85 ) |
|  | CD123 on plasmacytoid Dendritic Cell | Inverse variance weighted | 2 | 7.53E-03 | 0.55 (0.36 -0.85 ) |
|  | CD38 on IgD+ CD38dim B cell | Inverse variance weighted | 4 | 1.05E-02 | 0.69 (0.52 -0.92 ) |
|  | Monocyte Absolute Count | Wald ratio | 1 | 1.13E-02 | 3.55 (1.33 -9.47 ) |
|  | CD64 on CD14- CD16+ monocyte | Wald ratio | 1 | 1.26E-02 | 3.15 (1.28 -7.78 ) |
|  | CD3 on activated CD4 regulatory T cell | Inverse variance weighted | 4 | 1.26E-02 | 0.77 (0.62 -0.94 ) |
|  | CD3 on activated & secreting CD4 regulatory T cell | Inverse variance weighted | 4 | 1.29E-02 | 0.77 (0.63 -0.95 ) |
|  | CD38 on IgD+ B cell | Wald ratio | 1 | 1.41E-02 | 0.39 (0.19 -0.83 ) |
|  | CD38 on transitional B cell | Inverse variance weighted | 5 | 1.54E-02 | 0.72 (0.55 -0.94 ) |
|  | B cell %lymphocyte | Inverse variance weighted | 2 | 1.94E-02 | 0.38 (0.17 -0.86 ) |
|  | CD33- HLA DR+ Absolute Count | Wald ratio | 1 | 2.09E-02 | 0.51 (0.28 -0.90 ) |
|  | IgD+ CD24+ B cell %lymphocyte | Inverse variance weighted | 2 | 2.11E-02 | 0.38 (0.17 -0.86 ) |
|  | CD38 on naive-mature B cell | Inverse variance weighted | 3 | 2.34E-02 | 0.67 (0.48 -0.95 ) |
|  | IgD+ B cell %Lymphocyte | Inverse variance weighted | 2 | 2.58E-02 | 0.38 (0.16 -0.89 ) |
|  | CD38 on IgD- CD38dim B cell | Inverse variance weighted | 3 | 2.62E-02 | 0.70 (0.51 -0.96 ) |
|  | CD45RA- CD28- CD8+ T cell Absolute Count | Inverse variance weighted | 138 | 2.79E-02 | 1.00 (1.00 -1.00 ) |
|  | CD25 on CD45RA+ CD4 not regulatory T cell | Wald ratio | 1 | 3.09E-02 | 1.97 (1.06 -3.64 ) |
|  | CD25 on IgD- CD38- B cell | Inverse variance weighted | 3 | 3.51E-02 | 1.61 (1.03 -2.51 ) |
|  | CD3 on CD39+ secreting CD4 regulatory T cell | Inverse variance weighted | 2 | 3.56E-02 | 0.75 (0.58 -0.98 ) |
|  | T cell Absolute Count | Inverse variance weighted | 2 | 3.90E-02 | 1.83 (1.03 -3.26 ) |
|  | CD3 on CD39+ CD4+ T cell | Inverse variance weighted | 4 | 4.28E-02 | 0.81 (0.66 -0.99 ) |
|  | CD3 on secreting CD4 regulatory T cell | Inverse variance weighted | 2 | 4.37E-02 | 0.79 (0.62 -0.99 ) |
|  | CD3 on CD4 regulatory T cell | Inverse variance weighted | 2 | 4.44E-02 | 0.79 (0.63 -0.99 ) |
|  | CD62L- plasmacytoid Dendritic Cell Absolute Count | Wald ratio | 1 | 5.12E-02 | 0.58 (0.33 -1.00 ) |
|  | BAFF-R on IgD+ CD24+ B cell | Inverse variance weighted | 6 | 5.26E-02 | 1.17 (1.00 -1.37 ) |
|  | CCR7 on naive CD4+ T cell | Inverse variance weighted | 4 | 5.57E-02 | 1.37 (0.99 -1.89 ) |
|  | HVEM on T cell | Wald ratio | 1 | 5.78E-02 | 1.58 (0.99 -2.53 ) |
|  | HVEM on Effector Memory CD8+ T cell | Wald ratio | 1 | 5.78E-02 | 1.58 (0.98 -2.53 ) |
|  | HVEM on CD4+ T cell | Wald ratio | 1 | 5.78E-02 | 1.56 (0.99 -2.47 ) |
|  | HVEM on Central Memory CD4+ T cell | Wald ratio | 1 | 5.78E-02 | 1.58 (0.98 -2.53 ) |
|  | HVEM on CD45RA- CD4+ T cell | Wald ratio | 1 | 5.78E-02 | 1.54 (0.99 -2.42 ) |
|  | HVEM on Central Memory CD8+ T cell | Wald ratio | 1 | 5.78E-02 | 1.53 (0.99 -2.36 ) |
|  | CD3 on HLA DR+ CD8+ T cell | Wald ratio | 1 | 5.90E-02 | 0.59 (0.34 -1.02 ) |
|  | CD3 on CD39+ resting CD4 regulatory T cell | Wald ratio | 1 | 5.95E-02 | 0.64 (0.40 -1.02 ) |
|  | CD3 on Terminally Differentiated CD4+ T cell | Wald ratio | 1 | 6.14E-02 | 0.59 (0.33 -1.03 ) |
|  | CD3 on Central Memory CD8+ T cell | Wald ratio | 1 | 6.14E-02 | 0.71 (0.49 -1.02 ) |
|  | CD3 on CD8+ T cell | Wald ratio | 1 | 6.14E-02 | 0.70 (0.48 -1.02 ) |
|  | CD3 on CD39+ CD8+ T cell | Wald ratio | 1 | 6.14E-02 | 0.66 (0.42 -1.02 ) |
|  | CD11c+ HLA DR++ monocyte %monocyte | Wald ratio | 1 | 6.25E-02 | 0.44 (0.19 -1.04 ) |
|  | CD39 on CD39+ secreting CD4 regulatory T cell | Inverse variance weighted | 5 | 6.61E-02 | 0.90 (0.80 -1.01 ) |
|  | CD39 on CD39+ activated CD4 regulatory T cell | Inverse variance weighted | 5 | 6.98E-02 | 0.91 (0.82 -1.01 ) |
|  | CD25 on resting CD4 regulatory T cell | Wald ratio | 1 | 7.05E-02 | 27.14 (0.76 -971.58 ) |
|  | PDL-1 on monocyte | Inverse variance weighted | 2 | 7.27E-02 | 1.46 (0.97 -2.20 ) |
|  | CD45 on Natural Killer | Inverse variance weighted | 2 | 7.29E-02 | 1.74 (0.95 -3.18 ) |
|  | CD38 on IgD+ CD24- B cell | Inverse variance weighted | 2 | 7.30E-02 | 0.63 (0.38 -1.04 ) |
|  | Terminally Differentiated CD4+ T cell Absolute Count | Inverse variance weighted | 2 | 7.31E-02 | 0.53 (0.27 -1.06 ) |
|  | SSC-A on Natural Killer | Wald ratio | 1 | 8.17E-02 | 1.71 (0.93 -3.11 ) |
|  | CD3 on CD39+ activated CD4 regulatory T cell | Inverse variance weighted | 4 | 8.23E-02 | 0.83 (0.67 -1.02 ) |
|  | CD39 on CD39+ CD8+ T cell | Inverse variance weighted | 2 | 8.34E-02 | 0.60 (0.33 -1.07 ) |
|  | CD45 on CD33+ HLA DR+ CD14- | Wald ratio | 1 | 8.41E-02 | 1.87 (0.92 -3.80 ) |
|  | IgD- CD38- B cell Absolute Count | Wald ratio | 1 | 8.44E-02 | 1.85 (0.92 -3.73 ) |
|  | Naive CD8+ T cell %T cell | Inverse variance weighted | 3 | 8.50E-02 | 0.97 (0.94 -1.00 ) |
|  | CCR2 on CD14+ CD16- monocyte | Inverse variance weighted | 2 | 8.62E-02 | 1.45 (0.95 -2.20 ) |
|  | CCR2 on monocyte | Wald ratio | 1 | 8.79E-02 | 1.72 (0.92 -3.22 ) |
|  | CD39+ CD8+ T cell %T cell | Inverse variance weighted | 8 | 8.79E-02 | 0.90 (0.80 -1.02 ) |
|  | CD3 on Effector Memory CD8+ T cell | Inverse variance weighted | 3 | 8.81E-02 | 0.74 (0.52 -1.05 ) |
|  | CD33dim HLA DR- Absolute Count | Inverse variance weighted | 3 | 9.21E-02 | 0.90 (0.80 -1.02 ) |
|  | Lymphocyte Absolute Count | Inverse variance weighted | 2 | 9.59E-02 | 1.80 (0.90 -3.58 ) |
|  | BAFF-R on IgD+ B cell | Inverse variance weighted | 10 | 9.60E-02 | 1.12 (0.98 -1.27 ) |
|  | BAFF-R on B cell | Inverse variance weighted | 10 | 9.66E-02 | 1.12 (0.98 -1.28 ) |
|  | Effector Memory CD4-CD8- T cell %CD4-CD8- T cell | Inverse variance weighted | 4 | 9.77E-02 | 1.32 (0.95 -1.82 ) |
|  | SSC-A on plasmacytoid Dendritic Cell | Inverse variance weighted | 5 | 9.81E-02 | 0.66 (0.40 -1.08 ) |
|  | IgD on transitional B cell | Inverse variance weighted | 5 | 9.87E-02 | 1.26 (0.96 -1.65 ) |
|  | CD3 on Effector Memory CD4+ T cell | Inverse variance weighted | 2 | 9.87E-02 | 0.78 (0.59 -1.05 ) |
|  | CD39+ resting CD4 regulatory T cell %resting CD4 regulatory T cell | Inverse variance weighted | 5 | 9.90E-02 | 0.77 (0.57 -1.05 ) |
|  | Basophil Absolute Count | Inverse variance weighted | 3 | 1.01E-01 | 0.90 (0.80 -1.02 ) |
|  | BAFF-R on IgD+ CD24- B cell | Inverse variance weighted | 10 | 1.01E-01 | 1.12 (0.98 -1.27 ) |
|  | CD8+ and CD8dim T cell %leukocyte | Inverse variance weighted | 2 | 1.01E-01 | 2.72 (0.82 -9.03 ) |
|  | IgD on unswitched memory B cell | Inverse variance weighted | 5 | 1.01E-01 | 1.28 (0.95 -1.73 ) |
|  | CD127 on CD28+ CD45RA+ CD8+ T cell | Inverse variance weighted | 2 | 1.02E-01 | 1.57 (0.91 -2.71 ) |
|  | CCR7 on naive CD8+ T cell | Inverse variance weighted | 2 | 1.02E-01 | 1.61 (0.91 -2.84 ) |
|  | BAFF-R on IgD+ CD38+ B cell | Inverse variance weighted | 10 | 1.02E-01 | 1.12 (0.98 -1.28 ) |
|  | CD39+ secreting CD4 regulatory T cell %CD4 regulatory T cell | Inverse variance weighted | 6 | 1.04E-01 | 0.90 (0.80 -1.02 ) |
|  | BAFF-R on naive-mature B cell | Inverse variance weighted | 10 | 1.05E-01 | 1.11 (0.98 -1.27 ) |
|  | Terminally Differentiated CD4+ T cell %CD4+ T cell | Inverse variance weighted | 4 | 1.06E-01 | 0.69 (0.44 -1.08 ) |
|  | BAFF-R on IgD+ CD38- naive B cell | Inverse variance weighted | 8 | 1.07E-01 | 1.11 (0.98 -1.27 ) |
|  | CD4 on monocyte | Wald ratio | 1 | 1.07E-01 | 1.64 (0.90 -3.00 ) |
|  | CD39+ CD8+ T cell Absolute Count | Inverse variance weighted | 7 | 1.07E-01 | 0.91 (0.80 -1.02 ) |
|  | HLA DR on HLA DR+ T cell | Wald ratio | 1 | 1.08E-01 | 0.62 (0.34 -1.11 ) |
|  | HLA DR on HLA DR+ CD4+ T cell | Wald ratio | 1 | 1.08E-01 | 0.50 (0.21 -1.17 ) |
|  | BAFF-R on IgD+ CD38dim B cell | Inverse variance weighted | 10 | 1.08E-01 | 1.11 (0.98 -1.27 ) |
|  | BAFF-R on transitional B cell | Inverse variance weighted | 8 | 1.08E-01 | 1.13 (0.97 -1.30 ) |
|  | CD25 on transitional B cell | Inverse variance weighted | 2 | 1.08E-01 | 1.94 (0.86 -4.37 ) |
|  | CD39+ resting CD4 regulatory T cell %CD4 regulatory T cell | Inverse variance weighted | 11 | 1.10E-01 | 0.92 (0.83 -1.02 ) |
|  | CD3 on CD45RA- CD4+ T cell | Inverse variance weighted | 2 | 1.12E-01 | 0.81 (0.63 -1.05 ) |
|  | CD3 on Naive CD4+ T cell | Inverse variance weighted | 2 | 1.12E-01 | 0.85 (0.70 -1.04 ) |
|  | BAFF-R on CD20- B cell | Wald ratio | 1 | 1.17E-01 | 1.57 (0.89 -2.74 ) |
|  | CD39+ CD4+ T cell %CD4+ T cell | Inverse variance weighted | 6 | 1.18E-01 | 0.91 (0.81 -1.02 ) |
|  | CD3 on Central Memory CD4+ T cell | Inverse variance weighted | 2 | 1.18E-01 | 0.83 (0.66 -1.05 ) |
|  | CD39+ activated CD4 regulatory T cell %CD4 regulatory T cell | Inverse variance weighted | 6 | 1.19E-01 | 0.90 (0.79 -1.03 ) |
|  | FSC-A on Natural Killer T | Inverse variance weighted | 2 | 1.19E-01 | 2.06 (0.83 -5.11 ) |
|  | CD39+ CD4+ T cell %T cell | Inverse variance weighted | 6 | 1.19E-01 | 0.91 (0.81 -1.03 ) |
|  | CD3 on CD28+ CD4+ T cell | Inverse variance weighted | 2 | 1.21E-01 | 0.81 (0.61 -1.06 ) |
|  | CD64 on CD14- CD16- | Inverse variance weighted | 3 | 1.24E-01 | 1.55 (0.89 -2.71 ) |
|  | IgD on IgD+ CD38+ B cell | Inverse variance weighted | 6 | 1.24E-01 | 1.18 (0.96 -1.45 ) |
|  | CD25 on CD39+ CD4 regulatory T cell | Wald ratio | 1 | 1.28E-01 | 0.55 (0.25 -1.19 ) |
|  | CD25 on CD39+ activated CD4 regulatory T cell | Wald ratio | 1 | 1.28E-01 | 0.53 (0.23 -1.20 ) |
|  | BAFF-R on IgD+ CD38- B cell | Inverse variance weighted | 9 | 1.29E-01 | 1.11 (0.97 -1.27 ) |
|  | Central Memory CD8+ T cell Absolute Count | Wald ratio | 1 | 1.30E-01 | 1.89 (0.83 -4.33 ) |
|  | BAFF-R on CD20- CD38- B cell | Wald ratio | 1 | 1.31E-01 | 1.42 (0.90 -2.24 ) |
|  | CD20 on IgD+ CD24- B cell | Inverse variance weighted | 4 | 1.34E-01 | 1.35 (0.91 -1.99 ) |
|  | CD28+ CD45RA+ CD8dim T cell Absolute Count | Inverse variance weighted | 8 | 1.35E-01 | 1.02 (0.99 -1.05 ) |
|  | T/B cell | Inverse variance weighted | 2 | 1.37E-01 | 1.81 (0.83 -3.94 ) |
|  | CD3 on T cell | Inverse variance weighted | 3 | 1.38E-01 | 0.81 (0.61 -1.07 ) |
|  | HVEM on Effector Memory CD4+ T cell | Wald ratio | 1 | 1.38E-01 | 1.42 (0.89 -2.27 ) |
|  | Unswitched memory B cell Absolute Count | Inverse variance weighted | 2 | 1.39E-01 | 0.48 (0.18 -1.27 ) |
|  | CD20 on IgD+ CD38+ B cell | Inverse variance weighted | 5 | 1.44E-01 | 1.29 (0.92 -1.82 ) |
|  | HLA DR on CD14- CD16- | Inverse variance weighted | 4 | 1.44E-01 | 1.29 (0.92 -1.82 ) |
|  | IgD on IgD+ CD24- B cell | Inverse variance weighted | 5 | 1.45E-01 | 1.20 (0.94 -1.54 ) |
|  | Unswitched memory B cell %B cell | Wald ratio | 1 | 1.47E-01 | 0.50 (0.19 -1.28 ) |
|  | Terminally Differentiated CD4-CD8- T cell %CD4-CD8- T cell | Inverse variance weighted | 2 | 1.50E-01 | 0.68 (0.40 -1.15 ) |
|  | CD20- CD38- B cell %B cell | Wald ratio | 1 | 1.51E-01 | 1.49 (0.86 -2.56 ) |
|  | HVEM on Terminally Differentiated CD4+ T cell | Wald ratio | 1 | 1.53E-01 | 1.51 (0.86 -2.66 ) |
|  | HVEM on CD8+ T cell | Wald ratio | 1 | 1.53E-01 | 1.46 (0.87 -2.46 ) |
|  | CD62L on CD62L+ myeloid Dendritic Cell | Wald ratio | 1 | 1.53E-01 | 0.58 (0.28 -1.22 ) |
|  | Effector Memory CD4+ T cell Absolute Count | Wald ratio | 1 | 1.53E-01 | 1.42 (0.88 -2.28 ) |
|  | IgD on IgD+ CD38dim B cell | Inverse variance weighted | 5 | 1.55E-01 | 1.21 (0.93 -1.57 ) |
|  | CD40 on monocytes | Inverse variance weighted | 4 | 1.55E-01 | 0.84 (0.66 -1.07 ) |
|  | CD25 on secreting CD4 regulatory T cell | Inverse variance weighted | 2 | 1.58E-01 | 0.72 (0.45 -1.14 ) |
|  | CCR2 on granulocyte | Inverse variance weighted | 2 | 1.61E-01 | 1.59 (0.83 -3.04 ) |
|  | CD45RA- CD4+ T cell Absolute Count | Inverse variance weighted | 2 | 1.62E-01 | 1.34 (0.89 -2.01 ) |
|  | CD25 on CD45RA- CD4 not regulatory T cell | Inverse variance weighted | 2 | 1.62E-01 | 0.69 (0.40 -1.16 ) |
|  | CD25 on IgD+ CD38- unswitched memory B cell | Wald ratio | 1 | 1.63E-01 | 1.31 (0.90 -1.91 ) |
|  | CD45RA- CD4+ T cell %T cell | Inverse variance weighted | 2 | 1.64E-01 | 1.36 (0.88 -2.12 ) |
|  | CD25 on CD39+ secreting CD4 regulatory T cell | Inverse variance weighted | 2 | 1.65E-01 | 0.71 (0.44 -1.15 ) |
|  | CD25 on IgD+ CD38- B cell | Inverse variance weighted | 3 | 1.69E-01 | 0.70 (0.42 -1.16 ) |
|  | CD3 on CD28+ CD45RA- CD8+ T cell | Inverse variance weighted | 4 | 1.69E-01 | 0.80 (0.58 -1.10 ) |
|  | SSC-A on granulocyte | Inverse variance weighted | 3 | 1.69E-01 | 1.33 (0.89 -1.99 ) |
|  | BAFF-R on memory B cell | Inverse variance weighted | 7 | 1.73E-01 | 1.14 (0.94 -1.38 ) |
|  | HVEM on naive CD4+ T cell | Wald ratio | 1 | 1.74E-01 | 1.46 (0.85 -2.51 ) |
|  | BAFF-R on unswitched memory B cell | Inverse variance weighted | 7 | 1.77E-01 | 1.14 (0.94 -1.38 ) |
|  | CD14 on CD14+ CD16+ monocyte | Inverse variance weighted | 2 | 1.79E-01 | 0.68 (0.38 -1.20 ) |
|  | CD86 on monocyte | Wald ratio | 1 | 1.82E-01 | 1.85 (0.75 -4.59 ) |
|  | HLA DR+ CD8+ T cell %lymphocyte | Inverse variance weighted | 2 | 1.87E-01 | 1.25 (0.90 -1.73 ) |
|  | Immature Myeloid-Derived Suppressor Cells Absolute Count | Inverse variance weighted | 2 | 1.89E-01 | 0.82 (0.61 -1.10 ) |
|  | FSC-A on Natural Killer | Inverse variance weighted | 3 | 1.90E-01 | 1.36 (0.86 -2.16 ) |
|  | CD20 on IgD+ B cell | Inverse variance weighted | 5 | 1.91E-01 | 1.27 (0.89 -1.81 ) |
|  | Terminally Differentiated CD4-CD8- T cell %T cell | Inverse variance weighted | 3 | 1.91E-01 | 0.72 (0.44 -1.18 ) |
|  | CD4 on CD39+ resting CD4 regulatory T cell | Wald ratio | 1 | 1.91E-01 | 0.56 (0.23 -1.34 ) |
|  | CD28 on CD39+ resting CD4 regulatory T cell | Inverse variance weighted | 2 | 1.94E-01 | 0.68 (0.38 -1.22 ) |
|  | CD45 on CD33- HLA DR- | Inverse variance weighted | 2 | 1.95E-01 | 1.32 (0.87 -2.02 ) |
|  | CD45 on HLA DR+ T cell | Wald ratio | 1 | 1.96E-01 | 1.43 (0.83 -2.47 ) |
|  | SSC-A on HLA DR+ T cell | Wald ratio | 1 | 1.96E-01 | 1.60 (0.78 -3.27 ) |
|  | Unswitched memory B cell %lymphocyte | Inverse variance weighted | 4 | 1.99E-01 | 0.66 (0.35 -1.24 ) |
|  | CD45RA on naive CD8+ T cell | Inverse variance weighted | 2 | 1.99E-01 | 0.74 (0.46 -1.18 ) |
|  | CD3 on Natural Killer T | Inverse variance weighted | 2 | 2.05E-01 | 1.66 (0.76 -3.65 ) |
|  | CD39+ CD8+ T cell %CD8+ T cell | Inverse variance weighted | 6 | 2.05E-01 | 0.84 (0.64 -1.10 ) |
|  | Terminally Differentiated CD8+ T cell %T cell | Inverse variance weighted | 2 | 2.05E-01 | 0.72 (0.43 -1.20 ) |
|  | CCR2 on CD62L+ myeloid Dendritic Cell | Inverse variance weighted | 3 | 2.07E-01 | 1.21 (0.90 -1.61 ) |
|  | CD127 on CD28+ CD4+ T cell | Inverse variance weighted | 3 | 2.08E-01 | 1.36 (0.84 -2.21 ) |
|  | CD127 on granulocyte | Inverse variance weighted | 8 | 2.08E-01 | 0.87 (0.70 -1.08 ) |
|  | CD25 on CD39+ resting CD4 regulatory T cell | Wald ratio | 1 | 2.11E-01 | 0.47 (0.15 -1.53 ) |
|  | CD20 on B cell | Inverse variance weighted | 4 | 2.11E-01 | 1.28 (0.87 -1.89 ) |
|  | CD25 on IgD- CD38dim B cell | Inverse variance weighted | 3 | 2.16E-01 | 0.77 (0.50 -1.17 ) |
|  | HLA DR on CD33+ HLA DR+ CD14- | Inverse variance weighted | 2 | 2.18E-01 | 1.15 (0.92 -1.44 ) |
|  | CD20 on naive-mature B cell | Inverse variance weighted | 6 | 2.18E-01 | 1.24 (0.88 -1.75 ) |
|  | CD28- CD4-CD8- T cell %T cell | Inverse variance weighted | 3 | 2.21E-01 | 0.72 (0.43 -1.22 ) |
|  | CD8 on CD28+ CD45RA+ CD8+ T cell | Inverse variance weighted | 8 | 2.28E-01 | 1.13 (0.93 -1.36 ) |
|  | CD39 on CD39+ CD4+ T cell | Inverse variance weighted | 4 | 2.34E-01 | 0.92 (0.81 -1.05 ) |
|  | CD39 on granulocyte | Inverse variance weighted | 2 | 2.38E-01 | 0.59 (0.24 -1.42 ) |
|  | SSC-A on Natural Killer T | Inverse variance weighted | 2 | 2.38E-01 | 0.66 (0.33 -1.32 ) |
|  | IgD- CD38+ B cell %B cell | Wald ratio | 1 | 2.39E-01 | 0.56 (0.21 -1.47 ) |
|  | CD8 on Terminally Differentiated CD8+ T cell | Inverse variance weighted | 2 | 2.45E-01 | 1.23 (0.87 -1.76 ) |
|  | CD4 on HLA DR+ CD4+ T cell | Inverse variance weighted | 2 | 2.46E-01 | 1.42 (0.79 -2.55 ) |
|  | CD28 on CD39+ CD4+ T cell | Inverse variance weighted | 5 | 2.48E-01 | 0.88 (0.71 -1.09 ) |
|  | CD14 on Monocytic Myeloid-Derived Suppressor Cells | Wald ratio | 1 | 2.49E-01 | 0.76 (0.47 -1.22 ) |
|  | CD11c on CD62L+ myeloid Dendritic Cell | Wald ratio | 1 | 2.49E-01 | 1.25 (0.85 -1.83 ) |
|  | CD45 on HLA DR+ CD4+ T cell | Inverse variance weighted | 2 | 2.49E-01 | 1.45 (0.77 -2.75 ) |
|  | CD45RA- CD4+ T cell %CD4+ T cell | Inverse variance weighted | 4 | 2.50E-01 | 1.13 (0.92 -1.38 ) |
|  | CD28 on CD39+ secreting CD4 regulatory T cell | Inverse variance weighted | 2 | 2.51E-01 | 0.83 (0.60 -1.14 ) |
|  | CX3CR1 on monocyte | Inverse variance weighted | 3 | 2.59E-01 | 1.16 (0.89 -1.52 ) |
|  | CX3CR1 on CD14+ CD16- monocyte | Inverse variance weighted | 3 | 2.59E-01 | 1.15 (0.90 -1.46 ) |
|  | BAFF-R on IgD- CD38- B cell | Inverse variance weighted | 7 | 2.61E-01 | 1.14 (0.90 -1.45 ) |
|  | CD28+ CD45RA+ CD8dim T cell %T cell | Inverse variance weighted | 6 | 2.66E-01 | 0.91 (0.76 -1.08 ) |
|  | FSC-A on CD4+ T cell | Inverse variance weighted | 3 | 2.66E-01 | 0.59 (0.23 -1.50 ) |
|  | CCR2 on myeloid Dendritic Cell | Inverse variance weighted | 2 | 2.67E-01 | 1.22 (0.86 -1.75 ) |
|  | HLA DR on CD33- HLA DR+ | Inverse variance weighted | 2 | 2.70E-01 | 1.11 (0.93 -1.32 ) |
|  | CD28+ CD45RA+ CD8dim T cell %CD8dim T cell | Inverse variance weighted | 5 | 2.70E-01 | 0.91 (0.78 -1.07 ) |
|  | CD127 on CD45RA+ CD4+ T cell | Inverse variance weighted | 4 | 2.71E-01 | 1.34 (0.80 -2.25 ) |
|  | Memory B cell %lymphocyte | Inverse variance weighted | 2 | 2.73E-01 | 0.62 (0.26 -1.46 ) |
|  | CD4 on CD39+ CD4+ T cell | Inverse variance weighted | 5 | 2.74E-01 | 0.87 (0.69 -1.11 ) |
|  | CD8 on Effector Memory CD8+ T cell | Inverse variance weighted | 3 | 2.74E-01 | 1.19 (0.87 -1.63 ) |
|  | CD39+ CD4+ T cell Absolute Count | Inverse variance weighted | 5 | 2.75E-01 | 0.86 (0.66 -1.13 ) |
|  | CD8+ Natural Killer T %lymphocyte | Wald ratio | 1 | 2.76E-01 | 0.45 (0.11 -1.90 ) |
|  | Lymphocyte %leukocyte | Wald ratio | 1 | 2.76E-01 | 2.16 (0.54 -8.63 ) |
|  | Granulocyte %leukocyte | Wald ratio | 1 | 2.76E-01 | 0.47 (0.12 -1.84 ) |
|  | CD3 on naive CD8+ T cell | Inverse variance weighted | 4 | 2.80E-01 | 0.89 (0.71 -1.10 ) |
|  | CCR2 on CD62L+ plasmacytoid Dendritic Cell | Inverse variance weighted | 2 | 2.83E-01 | 1.20 (0.86 -1.68 ) |
|  | CCR2 on plasmacytoid Dendritic Cell | Inverse variance weighted | 2 | 2.83E-01 | 1.20 (0.86 -1.68 ) |
|  | CX3CR1 on CD14+ CD16+ monocyte | Inverse variance weighted | 3 | 2.83E-01 | 1.15 (0.89 -1.50 ) |
|  | CD45RA+ CD28- CD8+ T cell %T cell | Inverse variance weighted | 63 | 2.85E-01 | 1.00 (1.00 -1.00 ) |
|  | BAFF-R on IgD- CD24- B cell | Inverse variance weighted | 8 | 2.87E-01 | 1.14 (0.90 -1.45 ) |
|  | CD25++ CD8+ T cell %T cell | Inverse variance weighted | 2 | 2.87E-01 | 1.67 (0.65 -4.28 ) |
|  | CD25 on IgD+ CD24+ B cell | Inverse variance weighted | 3 | 2.92E-01 | 1.29 (0.80 -2.07 ) |
|  | Terminally Differentiated CD8+ T cell %CD8+ T cell | Inverse variance weighted | 3 | 2.95E-01 | 0.81 (0.54 -1.21 ) |
|  | CD25 on CD39+ CD4+ T cell | Inverse variance weighted | 5 | 2.95E-01 | 1.10 (0.92 -1.32 ) |
|  | BAFF-R on IgD- CD27- B cell | Inverse variance weighted | 8 | 2.98E-01 | 1.14 (0.89 -1.45 ) |
|  | IgD+ B cell %B cell | Wald ratio | 1 | 2.99E-01 | 1.67 (0.63 -4.38 ) |
|  | CD25++ CD4+ T cell Absolute Count | Wald ratio | 1 | 3.03E-01 | 0.68 (0.32 -1.42 ) |
|  | CD25++ CD4+ T cell %CD4+ T cell | Wald ratio | 1 | 3.03E-01 | 0.74 (0.41 -1.32 ) |
|  | CD25++ CD4+ T cell %T cell | Wald ratio | 1 | 3.03E-01 | 0.70 (0.36 -1.37 ) |
|  | CD25 on CD4+ T cell | Wald ratio | 1 | 3.03E-01 | 0.63 (0.26 -1.53 ) |
|  | CD28- CD127- CD25++ CD8+ T cell Absolute Count | Wald ratio | 1 | 3.04E-01 | 1.49 (0.70 -3.21 ) |
|  | CD3 on CD45RA+ CD4+ T cell | Inverse variance weighted | 4 | 3.04E-01 | 0.90 (0.74 -1.10 ) |
|  | CD28+ CD45RA- CD8dim T cell Absolute Count | Inverse variance weighted | 3 | 3.08E-01 | 1.10 (0.92 -1.31 ) |
|  | CD4RA on Terminally Differentiated CD4+ T cell | Inverse variance weighted | 3 | 3.09E-01 | 0.91 (0.75 -1.10 ) |
|  | BAFF-R on switched memory B cell | Inverse variance weighted | 9 | 3.09E-01 | 1.13 (0.89 -1.42 ) |
|  | CD27 on unswitched memory B cell | Inverse variance weighted | 11 | 3.13E-01 | 1.14 (0.88 -1.47 ) |
|  | CD11c on myeloid Dendritic Cell | Inverse variance weighted | 5 | 3.15E-01 | 1.23 (0.82 -1.83 ) |
|  | CD25 on switched memory B cell | Inverse variance weighted | 3 | 3.15E-01 | 0.83 (0.57 -1.20 ) |
|  | CD28 on CD4+ T cell | Inverse variance weighted | 2 | 3.19E-01 | 1.24 (0.81 -1.88 ) |
|  | CD28 on CD28+ CD4+ T cell | Inverse variance weighted | 2 | 3.22E-01 | 1.22 (0.82 -1.81 ) |
|  | CD4-CD8- T cell %leukocyte | Inverse variance weighted | 4 | 3.30E-01 | 0.78 (0.47 -1.29 ) |
|  | CD28- CD4-CD8- T cell Absolute Count | Inverse variance weighted | 2 | 3.32E-01 | 0.75 (0.43 -1.33 ) |
|  | CD28+ CD45RA- CD8dim T cell %CD8dim T cell | Inverse variance weighted | 2 | 3.34E-01 | 1.10 (0.91 -1.34 ) |
|  | Switched memory B cell %lymphocyte | Inverse variance weighted | 3 | 3.38E-01 | 0.76 (0.44 -1.33 ) |
|  | CD14+ CD16- monocyte %monocyte | Wald ratio | 1 | 3.39E-01 | 0.35 (0.04 -3.04 ) |
|  | CD16+ monocyte %monocyte | Wald ratio | 1 | 3.39E-01 | 2.87 (0.33 -24.79 ) |
|  | HLA DR on Dendritic Cell | Inverse variance weighted | 7 | 3.39E-01 | 1.07 (0.93 -1.23 ) |
|  | CD8 on CD28+ CD45RA- CD8+ T cell | Inverse variance weighted | 2 | 3.39E-01 | 0.80 (0.51 -1.26 ) |
|  | CD28+ CD45RA- CD8dim T cell %T cell | Inverse variance weighted | 3 | 3.41E-01 | 1.08 (0.92 -1.28 ) |
|  | CD24 on IgD- CD38- B cell | Inverse variance weighted | 2 | 3.44E-01 | 0.84 (0.58 -1.21 ) |
|  | FSC-A on granulocyte | Inverse variance weighted | 2 | 3.44E-01 | 1.35 (0.72 -2.54 ) |
|  | CD24 on IgD+ CD38- B cell | Inverse variance weighted | 2 | 3.45E-01 | 0.80 (0.51 -1.27 ) |
|  | CD45RA on CD39+ resting CD4 regulatory T cell | Wald ratio | 1 | 3.45E-01 | 1.15 (0.86 -1.52 ) |
|  | CD20 on IgD+ CD38dim B cell | Inverse variance weighted | 7 | 3.45E-01 | 1.15 (0.86 -1.55 ) |
|  | CD20 on unswitched memory B cell | Inverse variance weighted | 4 | 3.46E-01 | 1.25 (0.78 -2.01 ) |
|  | CD3- lymphocyte Absolute Count | Wald ratio | 1 | 3.49E-01 | 0.73 (0.39 -1.40 ) |
|  | CD45RA+ CD28- CD8+ T cell Absolute Count | Inverse variance weighted | 417 | 3.49E-01 | 1.00 (1.00 -1.00 ) |
|  | CD24 on IgD+ CD38- unswitched memory B cell | Inverse variance weighted | 2 | 3.53E-01 | 0.89 (0.69 -1.14 ) |
|  | CD24 on unswitched memory B cell | Inverse variance weighted | 2 | 3.55E-01 | 0.86 (0.64 -1.18 ) |
|  | CD25 on activated & secreting CD4 regulatory T cell | Wald ratio | 1 | 3.56E-01 | 0.74 (0.40 -1.39 ) |
|  | CD45 on Immature Myeloid-Derived Suppressor Cells | Inverse variance weighted | 2 | 3.56E-01 | 1.20 (0.81 -1.77 ) |
|  | CD14 on CD14+ CD16- monocyte | Wald ratio | 1 | 3.57E-01 | 1.32 (0.73 -2.37 ) |
|  | CD24 on IgD+ CD24+ B cell | Inverse variance weighted | 2 | 3.58E-01 | 0.88 (0.66 -1.16 ) |
|  | CD19 on IgD+ CD38- unswitched memory B cell | Inverse variance weighted | 3 | 3.60E-01 | 1.33 (0.72 -2.47 ) |
|  | CD45RA- CD28- CD8+ T cell %T cell | Inverse variance weighted | 40 | 3.62E-01 | 1.00 (1.00 -1.00 ) |
|  | CD25++ CD45RA+ CD4 not regulatory T cell Absolute Count | Inverse variance weighted | 3 | 3.62E-01 | 0.85 (0.61 -1.20 ) |
|  | CD20 on IgD+ CD38- B cell | Inverse variance weighted | 4 | 3.64E-01 | 1.26 (0.76 -2.09 ) |
|  | CD25 on IgD+ CD38+ B cell | Inverse variance weighted | 3 | 3.64E-01 | 1.42 (0.67 -3.02 ) |
|  | IgD+ CD38+ B cell Absolute Count | Inverse variance weighted | 2 | 3.68E-01 | 1.65 (0.55 -4.90 ) |
|  | CD62L on CD62L+ plasmacytoid Dendritic Cell | Inverse variance weighted | 3 | 3.69E-01 | 0.80 (0.49 -1.30 ) |
|  | CD28- CD8dim T cell %T cell | Inverse variance weighted | 3 | 3.70E-01 | 0.80 (0.49 -1.30 ) |
|  | CD62L on monocyte | Inverse variance weighted | 2 | 3.70E-01 | 1.15 (0.84 -1.58 ) |
|  | BAFF-R on CD24+ CD27+ B cell | Inverse variance weighted | 8 | 3.78E-01 | 1.12 (0.87 -1.43 ) |
|  | CD33+ HLA DR+ CD14dim %CD33+ HLA DR+ | Wald ratio | 1 | 3.79E-01 | 0.77 (0.42 -1.39 ) |
|  | CD62L- plasmacytoid Dendritic Cell %Dendritic Cell | Inverse variance weighted | 2 | 3.81E-01 | 0.76 (0.40 -1.41 ) |
|  | CD127 on CD45RA- CD4 not regulatory T cell | Wald ratio | 1 | 3.81E-01 | 1.50 (0.61 -3.71 ) |
|  | CD45RA+ CD8+ T cell %T cell | Inverse variance weighted | 3 | 3.82E-01 | 0.81 (0.50 -1.31 ) |
|  | CD28 on CD39+ activated CD4 regulatory T cell | Inverse variance weighted | 2 | 3.82E-01 | 1.26 (0.75 -2.11 ) |
|  | Plasma Blast-Plasma Cell %B cell | Wald ratio | 1 | 3.83E-01 | 1.90 (0.45 -8.10 ) |
|  | SSC-A on myeloid Dendritic Cell | Inverse variance weighted | 5 | 3.85E-01 | 1.22 (0.78 -1.90 ) |
|  | Terminally Differentiated CD4-CD8- T cell Absolute Count | Wald ratio | 1 | 3.87E-01 | 3.36 (0.22 -52.11 ) |
|  | CD28 on CD39+ CD8+ T cell | Inverse variance weighted | 2 | 3.89E-01 | 1.08 (0.91 -1.27 ) |
|  | CD8+ Natural Killer T %T cell | Wald ratio | 1 | 3.91E-01 | 0.51 (0.11 -2.38 ) |
|  | HLA DR+ Natural Killer %Natural Killer | Inverse variance weighted | 5 | 3.92E-01 | 1.12 (0.87 -1.44 ) |
|  | CD8 on CD39+ CD8+ T cell | Inverse variance weighted | 5 | 3.92E-01 | 1.22 (0.77 -1.92 ) |
|  | CCR2 on CD14+ CD16+ monocyte | Wald ratio | 1 | 3.97E-01 | 1.87 (0.44 -7.95 ) |
|  | BAFF-R on IgD+ CD38- unswitched memory B cell | Inverse variance weighted | 5 | 3.99E-01 | 1.12 (0.86 -1.47 ) |
|  | CD39+ resting CD4 regulatory T cell Absolute Count | Inverse variance weighted | 8 | 4.00E-01 | 0.92 (0.76 -1.11 ) |
|  | Naive-mature B cell %B cell | Wald ratio | 1 | 4.01E-01 | 1.61 (0.53 -4.87 ) |
|  | CD45RA on Terminally Differentiated CD8+ T cell | Wald ratio | 1 | 4.02E-01 | 1.42 (0.62 -3.24 ) |
|  | BAFF-R on IgD- CD38dim B cell | Wald ratio | 1 | 4.02E-01 | 1.39 (0.64 -3.02 ) |
|  | CD27 on Plasma Blast-Plasma Cell | Wald ratio | 1 | 4.02E-01 | 1.47 (0.60 -3.65 ) |
|  | CD4+CD8+ T cell Absolute Count | Inverse variance weighted | 2 | 4.12E-01 | 0.81 (0.48 -1.35 ) |
|  | CCR2 on CD14- CD16- | Inverse variance weighted | 3 | 4.14E-01 | 1.27 (0.72 -2.23 ) |
|  | HLA DR+ CD8+ T cell %T cell | Inverse variance weighted | 3 | 4.19E-01 | 1.16 (0.81 -1.64 ) |
|  | CD33dim HLA DR+ CD11b+ Absolute Count | Wald ratio | 1 | 4.22E-01 | 0.81 (0.48 -1.35 ) |
|  | CD27 on T cell | Wald ratio | 1 | 4.22E-01 | 1.19 (0.77 -1.84 ) |
|  | PDL-1 on CD14+ CD16+ monocyte | Wald ratio | 1 | 4.22E-01 | 0.68 (0.27 -1.73 ) |
|  | CX3CR1 on CD14- CD16+ monocyte | Wald ratio | 1 | 4.22E-01 | 1.26 (0.72 -2.21 ) |
|  | CD14 on CD33dim HLA DR+ CD11b+ | Wald ratio | 1 | 4.22E-01 | 1.18 (0.79 -1.75 ) |
|  | CD11c on monocyte | Wald ratio | 1 | 4.22E-01 | 0.82 (0.50 -1.33 ) |
|  | CD11b on CD14+ monocyte | Wald ratio | 1 | 4.22E-01 | 0.92 (0.74 -1.14 ) |
|  | CD11b on CD33+ HLA DR+ CD14dim | Wald ratio | 1 | 4.22E-01 | 0.89 (0.68 -1.17 ) |
|  | CD11b on Monocytic Myeloid-Derived Suppressor Cells | Wald ratio | 1 | 4.22E-01 | 0.89 (0.66 -1.19 ) |
|  | CD4+ CD8dim T cell %lymphocyte | Inverse variance weighted | 2 | 4.23E-01 | 0.79 (0.45 -1.40 ) |
|  | HLA DR on CD33+ HLA DR+ CD14dim | Inverse variance weighted | 3 | 4.26E-01 | 1.12 (0.85 -1.46 ) |
|  | CD20 on IgD- CD24- B cell | Inverse variance weighted | 2 | 4.30E-01 | 1.27 (0.70 -2.28 ) |
|  | CD8+ Natural Killer T Absolute Count | Inverse variance weighted | 2 | 4.31E-01 | 2.04 (0.35 -12.08 ) |
|  | CD25 on memory B cell | Inverse variance weighted | 4 | 4.31E-01 | 1.18 (0.78 -1.79 ) |
|  | HLA DR+ CD4+ T cell %T cell | Wald ratio | 1 | 4.33E-01 | 1.25 (0.72 -2.18 ) |
|  | Natural Killer Absolute Count | Inverse variance weighted | 3 | 4.35E-01 | 0.82 (0.50 -1.35 ) |
|  | CD45 on CD8+ T cell | Inverse variance weighted | 2 | 4.35E-01 | 1.12 (0.84 -1.50 ) |
|  | CD3 on resting CD4 regulatory T cell | Inverse variance weighted | 4 | 4.35E-01 | 0.91 (0.71 -1.16 ) |
|  | HLA DR on myeloid Dendritic Cell | Inverse variance weighted | 6 | 4.35E-01 | 1.07 (0.90 -1.27 ) |
|  | CD24 on transitional B cell | Wald ratio | 1 | 4.38E-01 | 1.43 (0.58 -3.51 ) |
|  | CD16-CD56 on HLA DR+ Natural Killer | Inverse variance weighted | 2 | 4.39E-01 | 0.72 (0.32 -1.64 ) |
|  | CD39+ secreting CD4 regulatory T cell %secreting CD4 regulatory T cell | Inverse variance weighted | 4 | 4.40E-01 | 0.95 (0.84 -1.08 ) |
|  | CD20 on memory B cell | Inverse variance weighted | 5 | 4.40E-01 | 1.18 (0.77 -1.81 ) |
|  | CD40 on CD14+ CD16- monocyte | Inverse variance weighted | 3 | 4.43E-01 | 0.88 (0.63 -1.22 ) |
|  | CD25 on CD24+ CD27+ B cell | Inverse variance weighted | 4 | 4.44E-01 | 1.17 (0.78 -1.77 ) |
|  | CD45 on granulocyte | Wald ratio | 1 | 4.46E-01 | 0.71 (0.30 -1.70 ) |
|  | CD4+ CD8dim T cell %leukocyte | Inverse variance weighted | 2 | 4.50E-01 | 0.80 (0.44 -1.44 ) |
|  | CD62L- monocyte Absolute Count | Inverse variance weighted | 2 | 4.50E-01 | 0.82 (0.48 -1.38 ) |
|  | CD45RA on resting CD4 regulatory T cell | Inverse variance weighted | 6 | 4.51E-01 | 0.95 (0.83 -1.09 ) |
|  | Activated & resting CD4 regulatory T cell %CD4 regulatory T cell | Inverse variance weighted | 5 | 4.51E-01 | 0.95 (0.83 -1.09 ) |
|  | Secreting CD4 regulatory T cell %CD4 regulatory T cell | Inverse variance weighted | 5 | 4.52E-01 | 1.05 (0.92 -1.21 ) |
|  | CD28+ CD45RA- CD8+ T cell Absolute Count | Inverse variance weighted | 2 | 4.53E-01 | 1.09 (0.86 -1.38 ) |
|  | CD19 on CD24+ CD27+ B cell | Inverse variance weighted | 3 | 4.55E-01 | 0.90 (0.67 -1.20 ) |
|  | Transitional B cell Absolute Count | Wald ratio | 1 | 4.58E-01 | 1.52 (0.50 -4.62 ) |
|  | CD24 on IgD+ CD38+ B cell | Inverse variance weighted | 2 | 4.60E-01 | 1.33 (0.63 -2.82 ) |
|  | CD4-CD8- T cell Absolute Count | Inverse variance weighted | 2 | 4.60E-01 | 0.79 (0.43 -1.47 ) |
|  | FSC-A on plasmacytoid Dendritic Cell | Inverse variance weighted | 4 | 4.64E-01 | 1.22 (0.72 -2.07 ) |
|  | CD19 on memory B cell | Inverse variance weighted | 3 | 4.65E-01 | 0.89 (0.64 -1.23 ) |
|  | CD25 on unswitched memory B cell | Inverse variance weighted | 4 | 4.69E-01 | 1.17 (0.76 -1.82 ) |
|  | Natural Killer T %lymphocyte | Inverse variance weighted | 7 | 4.71E-01 | 1.14 (0.80 -1.62 ) |
|  | CD8dim T cell %T cell | Inverse variance weighted | 2 | 4.72E-01 | 0.78 (0.39 -1.54 ) |
|  | CD80 on CD62L+ myeloid Dendritic Cell | Inverse variance weighted | 3 | 4.78E-01 | 1.21 (0.71 -2.05 ) |
|  | Activated & secreting CD4 regulatory T cell Absolute Count | Inverse variance weighted | 4 | 4.86E-01 | 1.06 (0.90 -1.26 ) |
|  | CD14+ CD16- monocyte Absolute Count | Inverse variance weighted | 3 | 4.86E-01 | 1.42 (0.53 -3.86 ) |
|  | CD8dim T cell %leukocyte | Inverse variance weighted | 2 | 4.87E-01 | 0.77 (0.38 -1.59 ) |
|  | Secreting CD4 regulatory T cell Absolute Count | Inverse variance weighted | 4 | 4.87E-01 | 1.06 (0.90 -1.26 ) |
|  | IgD+ CD38+ B cell %lymphocyte | Wald ratio | 1 | 4.90E-01 | 4.11 (0.07 -226.73 ) |
|  | CD3 on HLA DR+ CD4+ T cell | Inverse variance weighted | 2 | 4.91E-01 | 0.80 (0.42 -1.51 ) |
|  | CD8 on CD8+ T cell | Wald ratio | 1 | 4.92E-01 | 0.86 (0.55 -1.34 ) |
|  | CD19 on IgD- CD38dim B cell | Inverse variance weighted | 4 | 4.96E-01 | 0.92 (0.71 -1.18 ) |
|  | CD4+ CD8dim T cell Absolute Count | Inverse variance weighted | 2 | 4.96E-01 | 0.79 (0.41 -1.54 ) |
|  | CD80 on granulocyte | Inverse variance weighted | 3 | 5.01E-01 | 1.18 (0.72 -1.94 ) |
|  | Activated CD4 regulatory T cell Absolute Count | Inverse variance weighted | 2 | 5.03E-01 | 1.17 (0.74 -1.85 ) |
|  | CD62L- Dendritic Cell %Dendritic Cell | Inverse variance weighted | 4 | 5.03E-01 | 0.92 (0.71 -1.18 ) |
|  | CD34 on Hematopoietic Stem Cell | Inverse variance weighted | 4 | 5.06E-01 | 1.14 (0.78 -1.66 ) |
|  | CD19 on CD20- B cell | Wald ratio | 1 | 5.08E-01 | 1.17 (0.74 -1.84 ) |
|  | HLA DR++ monocyte Absolute Count | Wald ratio | 1 | 5.09E-01 | 1.22 (0.68 -2.18 ) |
|  | Dendritic Cell Absolute Count | Inverse variance weighted | 2 | 5.11E-01 | 0.89 (0.63 -1.26 ) |
|  | CD25 on IgD+ CD38- naive B cell | Inverse variance weighted | 3 | 5.12E-01 | 1.27 (0.62 -2.63 ) |
|  | PDL-1 on CD14+ CD16- monocyte | Wald ratio | 1 | 5.18E-01 | 0.73 (0.27 -1.92 ) |
|  | CD20 on IgD- CD38- B cell | Inverse variance weighted | 4 | 5.21E-01 | 1.18 (0.71 -1.96 ) |
|  | CD19 on IgD+ B cell | Inverse variance weighted | 2 | 5.24E-01 | 1.57 (0.39 -6.30 ) |
|  | CD14- CD16+ monocyte %monocyte | Wald ratio | 1 | 5.29E-01 | 1.35 (0.53 -3.42 ) |
|  | B cell Absolute Count | Wald ratio | 1 | 5.37E-01 | 1.40 (0.48 -4.12 ) |
|  | CD3 on CD28+ CD45RA+ CD8+ T cell | Inverse variance weighted | 3 | 5.40E-01 | 0.90 (0.65 -1.25 ) |
|  | CD86+ plasmacytoid Dendritic Cell %Dendritic Cell | Wald ratio | 1 | 5.43E-01 | 1.28 (0.58 -2.83 ) |
|  | HLA DR on CD14+ monocyte | Inverse variance weighted | 5 | 5.44E-01 | 1.05 (0.89 -1.25 ) |
|  | HLA DR on CD14+ CD16- monocyte | Inverse variance weighted | 5 | 5.44E-01 | 1.05 (0.89 -1.24 ) |
|  | FSC-A on CD14+ monocyte | Wald ratio | 1 | 5.45E-01 | 1.88 (0.24 -14.57 ) |
|  | CD4 on naive CD4+ T cell | Inverse variance weighted | 5 | 5.46E-01 | 0.86 (0.52 -1.42 ) |
|  | CD45 on CD33- HLA DR+ | Inverse variance weighted | 2 | 5.48E-01 | 0.84 (0.47 -1.49 ) |
|  | CD4 on Effector Memory CD4+ T cell | Inverse variance weighted | 3 | 5.48E-01 | 1.10 (0.80 -1.52 ) |
|  | CD3- lymphocyte %leukocyte | Wald ratio | 1 | 5.50E-01 | 1.38 (0.48 -3.98 ) |
|  | CD28- CD4-CD8- T cell %CD4-CD8- T cell | Wald ratio | 1 | 5.50E-01 | 0.75 (0.29 -1.94 ) |
|  | CD28+ CD4-CD8- T cell %CD4-CD8- T cell | Wald ratio | 1 | 5.50E-01 | 1.34 (0.52 -3.47 ) |
|  | CD28- CD8dim T cell %CD8dim T cell | Wald ratio | 1 | 5.50E-01 | 0.73 (0.26 -2.03 ) |
|  | CD4 regulatory T cell %CD4+ T cell | Inverse variance weighted | 2 | 5.56E-01 | 1.32 (0.53 -3.29 ) |
|  | CD45RA- CD28- CD8+ T cell %CD8+ T cell | Inverse variance weighted | 10 | 5.57E-01 | 1.00 (1.00 -1.01 ) |
|  | SSC-A on T cell | Wald ratio | 1 | 5.59E-01 | 0.65 (0.15 -2.80 ) |
|  | CD127 on CD28+ CD45RA- CD8+ T cell | Wald ratio | 1 | 5.60E-01 | 1.11 (0.79 -1.55 ) |
|  | CD4 on CD4+ T cell | Wald ratio | 1 | 5.62E-01 | 1.17 (0.68 -2.02 ) |
|  | CD4 on CD4 regulatory T cell | Wald ratio | 1 | 5.62E-01 | 1.20 (0.65 -2.23 ) |
|  | CD4 on resting CD4 regulatory T cell | Wald ratio | 1 | 5.62E-01 | 1.27 (0.57 -2.86 ) |
|  | CD4 on activated CD4 regulatory T cell | Wald ratio | 1 | 5.62E-01 | 1.20 (0.65 -2.19 ) |
|  | CD4 on secreting CD4 regulatory T cell | Wald ratio | 1 | 5.62E-01 | 1.24 (0.60 -2.58 ) |
|  | CD4 on activated & secreting CD4 regulatory T cell | Wald ratio | 1 | 5.62E-01 | 1.21 (0.64 -2.27 ) |
|  | Memory B cell %B cell | Inverse variance weighted | 2 | 5.64E-01 | 0.79 (0.35 -1.78 ) |
|  | CD33+ HLA DR+ CD14dim Absolute Count | Inverse variance weighted | 2 | 5.65E-01 | 1.12 (0.76 -1.65 ) |
|  | CD45 on CD14+ monocyte | Wald ratio | 1 | 5.66E-01 | 1.57 (0.33 -7.39 ) |
|  | CD27 on CD24+ CD27+ B cell | Inverse variance weighted | 9 | 5.68E-01 | 0.94 (0.77 -1.16 ) |
|  | Plasma Blast-Plasma Cell %lymphocyte | Wald ratio | 1 | 5.75E-01 | 0.74 (0.26 -2.12 ) |
|  | CD24+ CD27+ B cell %B cell | Inverse variance weighted | 3 | 5.79E-01 | 0.85 (0.48 -1.51 ) |
|  | CD4+ T cell %leukocyte | Wald ratio | 1 | 5.80E-01 | 1.45 (0.39 -5.39 ) |
|  | Immature Myeloid-Derived Suppressor Cells %CD33dim HLA DR- CD66b- | Inverse variance weighted | 2 | 5.84E-01 | 0.91 (0.66 -1.27 ) |
|  | IgD- CD27- B cell Absolute Count | Inverse variance weighted | 2 | 5.85E-01 | 0.88 (0.55 -1.40 ) |
|  | CX3CR1 on CD14- CD16- | Inverse variance weighted | 3 | 5.85E-01 | 1.22 (0.60 -2.50 ) |
|  | CD28 on CD45RA- CD4 not regulatory T cell | Inverse variance weighted | 3 | 5.89E-01 | 1.11 (0.76 -1.61 ) |
|  | Naive CD8+ T cell Absolute Count | Wald ratio | 1 | 5.90E-01 | 0.63 (0.12 -3.35 ) |
|  | Central Memory CD4+ T cell Absolute Count | Wald ratio | 1 | 5.93E-01 | 1.26 (0.54 -2.96 ) |
|  | CD4+ T cell Absolute Count | Wald ratio | 1 | 5.93E-01 | 1.23 (0.58 -2.58 ) |
|  | HLA DR on plasmacytoid Dendritic Cell | Inverse variance weighted | 8 | 5.99E-01 | 1.03 (0.91 -1.17 ) |
|  | Monocytic Myeloid-Derived Suppressor Cells Absolute Count | Inverse variance weighted | 5 | 6.03E-01 | 0.95 (0.79 -1.15 ) |
|  | CD45RA+ CD28- CD8+ T cell %CD8+ T cell | Wald ratio | 1 | 6.03E-01 | 0.99 (0.94 -1.04 ) |
|  | Natural Killer %lymphocyte | Inverse variance weighted | 3 | 6.04E-01 | 0.89 (0.59 -1.36 ) |
|  | CD33dim HLA DR+ CD11b- Absolute Count | Inverse variance weighted | 2 | 6.06E-01 | 1.13 (0.71 -1.80 ) |
|  | CD3 on HLA DR+ T cell | Inverse variance weighted | 2 | 6.09E-01 | 0.83 (0.41 -1.70 ) |
|  | HLA DR on CD14+ CD16+ monocyte | Inverse variance weighted | 4 | 6.09E-01 | 1.07 (0.82 -1.40 ) |
|  | CD80 on myeloid Dendritic Cell | Inverse variance weighted | 4 | 6.11E-01 | 1.12 (0.73 -1.70 ) |
|  | CD39+ secreting CD4 regulatory T cell Absolute Count | Inverse variance weighted | 4 | 6.14E-01 | 0.97 (0.85 -1.10 ) |
|  | HVEM on naive CD8+ T cell | Inverse variance weighted | 2 | 6.17E-01 | 1.17 (0.64 -2.13 ) |
|  | CD20 on IgD+ CD24+ B cell | Inverse variance weighted | 2 | 6.21E-01 | 1.15 (0.66 -2.03 ) |
|  | IgD+ CD24- B cell %B cell | Wald ratio | 1 | 6.22E-01 | 0.88 (0.52 -1.48 ) |
|  | HLA DR+ T cell%lymphocyte | Inverse variance weighted | 4 | 6.26E-01 | 1.11 (0.72 -1.72 ) |
|  | CD3 on CD28- CD8+ T cell | Inverse variance weighted | 2 | 6.28E-01 | 0.84 (0.43 -1.67 ) |
|  | CD27 on IgD- CD38dim B cell | Inverse variance weighted | 6 | 6.28E-01 | 0.95 (0.78 -1.16 ) |
|  | Activated & secreting CD4 regulatory T cell %CD4+ T cell | Inverse variance weighted | 9 | 6.33E-01 | 1.03 (0.93 -1.14 ) |
|  | HLA DR on B cell | Inverse variance weighted | 9 | 6.35E-01 | 1.06 (0.84 -1.32 ) |
|  | CCR2 on monocyte | Inverse variance weighted | 2 | 6.35E-01 | 1.15 (0.65 -2.01 ) |
|  | CD11c+ CD62L- monocyte Absolute Count | Wald ratio | 1 | 6.38E-01 | 1.33 (0.40 -4.44 ) |
|  | CD4+CD8+ T cell %leukocyte | Wald ratio | 1 | 6.39E-01 | 0.89 (0.55 -1.44 ) |
|  | CD45 on Natural Killer T | Wald ratio | 1 | 6.40E-01 | 0.81 (0.33 -1.97 ) |
|  | CD40 on CD14+ CD16+ monocyte | Inverse variance weighted | 3 | 6.40E-01 | 0.94 (0.73 -1.21 ) |
|  | CD25 on IgD+ CD24- B cell | Inverse variance weighted | 3 | 6.46E-01 | 1.21 (0.53 -2.75 ) |
|  | HLA DR+ T cell%T cell | Inverse variance weighted | 4 | 6.47E-01 | 1.10 (0.73 -1.64 ) |
|  | CD3 on Terminally Differentiated CD8+ T cell | Inverse variance weighted | 2 | 6.47E-01 | 0.81 (0.34 -1.97 ) |
|  | CD20 on CD24+ CD27+ B cell | Inverse variance weighted | 5 | 6.49E-01 | 1.09 (0.75 -1.60 ) |
|  | CD4-CD8- T cell %T cell | Inverse variance weighted | 3 | 6.50E-01 | 0.88 (0.50 -1.53 ) |
|  | CD25 on B cell | Inverse variance weighted | 3 | 6.51E-01 | 0.91 (0.61 -1.36 ) |
|  | Granulocytic Myeloid-Derived Suppressor Cells Absolute Count | Inverse variance weighted | 2 | 6.55E-01 | 1.11 (0.70 -1.75 ) |
|  | CD28 on CD4 regulatory T cell | Wald ratio | 1 | 6.57E-01 | 0.88 (0.49 -1.56 ) |
|  | Naive-mature B cell %lymphocyte | Wald ratio | 1 | 6.58E-01 | 1.09 (0.75 -1.56 ) |
|  | IgD+ CD24- B cell %lymphocyte | Wald ratio | 1 | 6.58E-01 | 1.09 (0.75 -1.59 ) |
|  | SSC-A on CD4+ T cell | Inverse variance weighted | 4 | 6.59E-01 | 0.87 (0.47 -1.61 ) |
|  | CD19 on B cell | Wald ratio | 1 | 6.59E-01 | 1.22 (0.51 -2.89 ) |
|  | CD8+ T cell Absolute Count | Wald ratio | 1 | 6.62E-01 | 0.85 (0.42 -1.74 ) |
|  | CD8+ T cell %leukocyte | Wald ratio | 1 | 6.62E-01 | 0.85 (0.40 -1.79 ) |
|  | CD127- CD8+ T cell Absolute Count | Wald ratio | 1 | 6.62E-01 | 0.85 (0.42 -1.74 ) |
|  | T cell %lymphocyte | Inverse variance weighted | 2 | 6.63E-01 | 1.11 (0.68 -1.82 ) |
|  | CD14- CD16+ monocyte Absolute Count | Wald ratio | 1 | 6.64E-01 | 1.15 (0.62 -2.11 ) |
|  | CCR2 on CD14- CD16+ monocyte | Wald ratio | 1 | 6.64E-01 | 0.80 (0.28 -2.24 ) |
|  | BAFF-R on IgD- CD38+ B cell | Inverse variance weighted | 3 | 6.64E-01 | 1.15 (0.61 -2.14 ) |
|  | CD14- CD16- Absolute Count | Inverse variance weighted | 3 | 6.73E-01 | 0.88 (0.48 -1.62 ) |
|  | CD8 on CD28- CD8+ T cell | Inverse variance weighted | 2 | 6.78E-01 | 1.14 (0.62 -2.08 ) |
|  | Terminally Differentiated CD4+ T cell %T cell | Inverse variance weighted | 5 | 6.78E-01 | 0.93 (0.67 -1.29 ) |
|  | IgD- CD38dim B cell %lymphocyte | Inverse variance weighted | 2 | 6.83E-01 | 0.86 (0.41 -1.80 ) |
|  | IgD+ CD38- B cell Absolute Count | Wald ratio | 1 | 6.86E-01 | 1.68 (0.14 -20.83 ) |
|  | CD28+ CD45RA+ CD8+ T cell %T cell | Inverse variance weighted | 37 | 6.87E-01 | 1.00 (0.99 -1.02 ) |
|  | IgD- CD27- B cell %lymphocyte | Inverse variance weighted | 2 | 6.89E-01 | 1.10 (0.69 -1.76 ) |
|  | Effector Memory CD8+ T cell %CD8+ T cell | Inverse variance weighted | 3 | 6.91E-01 | 1.05 (0.83 -1.33 ) |
|  | CD20 on switched memory B cell | Inverse variance weighted | 7 | 6.99E-01 | 1.06 (0.78 -1.46 ) |
|  | CD28- CD8dim T cell Absolute Count | Inverse variance weighted | 2 | 7.01E-01 | 0.87 (0.44 -1.74 ) |
|  | CD45RA+ CD8+ T cell Absolute Count | Inverse variance weighted | 3 | 7.02E-01 | 1.25 (0.40 -3.84 ) |
|  | Basophil %CD33dim HLA DR- CD66b- | Inverse variance weighted | 3 | 7.03E-01 | 1.05 (0.83 -1.32 ) |
|  | CD27 on IgD+ CD24+ B cell | Inverse variance weighted | 6 | 7.03E-01 | 0.95 (0.74 -1.22 ) |
|  | CD64 on CD14+ CD16+ monocyte | Inverse variance weighted | 2 | 7.05E-01 | 0.84 (0.34 -2.09 ) |
|  | Memory B cell Absolute Count | Wald ratio | 1 | 7.08E-01 | 0.66 (0.08 -5.67 ) |
|  | IgD+ CD24+ B cell Absolute Count | Wald ratio | 1 | 7.08E-01 | 0.70 (0.11 -4.61 ) |
|  | CD24+ CD27+ B cell Absolute Count | Wald ratio | 1 | 7.08E-01 | 0.66 (0.07 -5.90 ) |
|  | CD28- CD8+ T cell %CD8+ T cell | Inverse variance weighted | 3 | 7.10E-01 | 0.89 (0.49 -1.62 ) |
|  | CD19 on CD20- CD38- B cell | Wald ratio | 1 | 7.13E-01 | 0.80 (0.24 -2.66 ) |
|  | CD11b on CD33dim HLA DR- | Wald ratio | 1 | 7.17E-01 | 0.93 (0.63 -1.37 ) |
|  | CD11b on basophil | Wald ratio | 1 | 7.17E-01 | 0.93 (0.64 -1.36 ) |
|  | HLA DR on HLA DR+ Natural Killer | Inverse variance weighted | 5 | 7.17E-01 | 1.07 (0.73 -1.58 ) |
|  | CD33 on Granulocytic Myeloid-Derived Suppressor Cells | Inverse variance weighted | 3 | 7.19E-01 | 0.97 (0.83 -1.14 ) |
|  | CD33 on basophil | Inverse variance weighted | 7 | 7.20E-01 | 0.98 (0.87 -1.10 ) |
|  | Activated & secreting CD4 regulatory T cell %CD4 regulatory T cell | Inverse variance weighted | 8 | 7.21E-01 | 1.02 (0.91 -1.14 ) |
|  | Activated CD4 regulatory T cell %CD4 regulatory T cell | Inverse variance weighted | 3 | 7.23E-01 | 0.96 (0.75 -1.22 ) |
|  | CD86 on myeloid Dendritic Cell | Wald ratio | 1 | 7.24E-01 | 0.93 (0.61 -1.41 ) |
|  | CD33 on CD33dim HLA DR+ CD11b- | Inverse variance weighted | 7 | 7.27E-01 | 1.02 (0.91 -1.14 ) |
|  | CD16 on CD14+ CD16+ monocyte | Inverse variance weighted | 4 | 7.28E-01 | 1.04 (0.82 -1.32 ) |
|  | CD39 on monocyte | Inverse variance weighted | 2 | 7.29E-01 | 0.80 (0.23 -2.76 ) |
|  | CD86+ myeloid Dendritic Cell %Dendritic Cell | Inverse variance weighted | 3 | 7.33E-01 | 0.96 (0.74 -1.23 ) |
|  | CD86+ myeloid Dendritic Cell Absolute Count | Inverse variance weighted | 3 | 7.34E-01 | 0.96 (0.74 -1.23 ) |
|  | CD62L- CD86+ myeloid Dendritic Cell Absolute Count | Inverse variance weighted | 3 | 7.34E-01 | 0.96 (0.77 -1.20 ) |
|  | CD40 on CD14- CD16+ monocyte | Inverse variance weighted | 7 | 7.35E-01 | 0.96 (0.78 -1.19 ) |
|  | HLA DR+ Natural Killer Absolute Count | Inverse variance weighted | 3 | 7.35E-01 | 1.05 (0.79 -1.40 ) |
|  | CD8 on Central Memory CD8+ T cell | Inverse variance weighted | 2 | 7.36E-01 | 0.93 (0.61 -1.42 ) |
|  | CD62L- Dendritic Cell Absolute Count | Inverse variance weighted | 3 | 7.36E-01 | 0.96 (0.73 -1.25 ) |
|  | Myeloid Dendritic Cell %Dendritic Cell | Inverse variance weighted | 2 | 7.40E-01 | 0.95 (0.68 -1.32 ) |
|  | Myeloid Dendritic Cell Absolute Count | Inverse variance weighted | 2 | 7.42E-01 | 0.95 (0.69 -1.30 ) |
|  | HLA DR+ Natural Killer %CD3- lymphocyte | Inverse variance weighted | 2 | 7.44E-01 | 1.05 (0.79 -1.40 ) |
|  | CD11c on granulocyte | Inverse variance weighted | 2 | 7.46E-01 | 0.95 (0.67 -1.33 ) |
|  | CD25++ CD45RA- CD4 not regulatory T cell %T cell | Inverse variance weighted | 3 | 7.46E-01 | 1.04 (0.80 -1.36 ) |
|  | CD4 on CD39+ secreting CD4 regulatory T cell | Inverse variance weighted | 3 | 7.48E-01 | 0.94 (0.65 -1.36 ) |
|  | CD28- CD8+ T cell Absolute Count | Inverse variance weighted | 3 | 7.51E-01 | 0.91 (0.51 -1.63 ) |
|  | CD28+ CD45RA+ CD8+ T cell Absolute Count | Inverse variance weighted | 3 | 7.52E-01 | 1.17 (0.43 -3.17 ) |
|  | CD45RA on naive CD4+ T cell | Inverse variance weighted | 10 | 7.53E-01 | 1.03 (0.87 -1.22 ) |
|  | CD25++ CD45RA+ CD4 not regulatory T cell %T cell | Inverse variance weighted | 4 | 7.56E-01 | 1.05 (0.75 -1.47 ) |
|  | Naive CD4+ T cell Absolute Count | Inverse variance weighted | 2 | 7.57E-01 | 1.21 (0.37 -3.96 ) |
|  | CD25++ CD45RA+ CD4 not regulatory T cell %CD4+ T cell | Inverse variance weighted | 4 | 7.58E-01 | 1.05 (0.76 -1.45 ) |
|  | HLA DR++ monocyte %leukocyte | Inverse variance weighted | 2 | 7.58E-01 | 1.10 (0.61 -1.96 ) |
|  | SSC-A on HLA DR+ Natural Killer | Inverse variance weighted | 3 | 7.58E-01 | 0.95 (0.67 -1.33 ) |
|  | CD20 on transitional B cell | Inverse variance weighted | 2 | 7.66E-01 | 1.07 (0.67 -1.71 ) |
|  | Natural Killer T %T cell | Inverse variance weighted | 5 | 7.67E-01 | 1.06 (0.71 -1.59 ) |
|  | CD16 on CD14- CD16+ monocyte | Inverse variance weighted | 7 | 7.67E-01 | 1.02 (0.87 -1.20 ) |
|  | CD4 regulatory T cell %T cell | Wald ratio | 1 | 7.69E-01 | 0.83 (0.23 -2.92 ) |
|  | CD62L- monocyte %monocyte | Inverse variance weighted | 3 | 7.69E-01 | 0.90 (0.45 -1.80 ) |
|  | IgD+ CD38- B cell %B cell | Inverse variance weighted | 2 | 7.71E-01 | 0.87 (0.35 -2.19 ) |
|  | CD8dim T cell Absolute Count | Inverse variance weighted | 3 | 7.72E-01 | 0.91 (0.47 -1.74 ) |
|  | Resting CD4 regulatory T cell %CD4 regulatory T cell | Inverse variance weighted | 9 | 7.73E-01 | 1.02 (0.90 -1.15 ) |
|  | HLA DR on monocyte | Inverse variance weighted | 4 | 7.76E-01 | 1.03 (0.85 -1.24 ) |
|  | Natural Killer %CD3- lymphocyte | Inverse variance weighted | 5 | 7.76E-01 | 0.96 (0.72 -1.27 ) |
|  | CD45 on B cell | Inverse variance weighted | 2 | 7.81E-01 | 0.90 (0.43 -1.89 ) |
|  | CD8 on Natural Killer T | Inverse variance weighted | 2 | 7.87E-01 | 1.08 (0.61 -1.90 ) |
|  | CD28 on CD45RA+ CD4+ T cell | Inverse variance weighted | 2 | 7.96E-01 | 0.95 (0.65 -1.38 ) |
|  | CD16-CD56 on Natural Killer T | Inverse variance weighted | 4 | 7.98E-01 | 0.96 (0.70 -1.32 ) |
|  | HLA DR on CD14- CD16+ monocyte | Inverse variance weighted | 8 | 7.98E-01 | 0.97 (0.74 -1.26 ) |
|  | CD19 on IgD- CD38- B cell | Wald ratio | 1 | 7.98E-01 | 1.13 (0.43 -2.98 ) |
|  | CD33- HLA DR- Absolute Count | Wald ratio | 1 | 8.03E-01 | 0.91 (0.44 -1.88 ) |
|  | Plasmacytoid Dendritic Cell %Dendritic Cell | Inverse variance weighted | 2 | 8.03E-01 | 0.91 (0.42 -1.95 ) |
|  | CD28+ CD4-CD8- T cell %T cell | Wald ratio | 1 | 8.05E-01 | 1.14 (0.40 -3.24 ) |
|  | HLA DR+ CD8+ T cell Absolute Count | Inverse variance weighted | 3 | 8.05E-01 | 1.08 (0.59 -1.97 ) |
|  | CD4-CD8- Natural Killer T Absolute Count | Wald ratio | 1 | 8.05E-01 | 1.11 (0.49 -2.50 ) |
|  | CD62L- CD86+ myeloid Dendritic Cell %Dendritic Cell | Inverse variance weighted | 4 | 8.09E-01 | 0.97 (0.78 -1.21 ) |
|  | CD28+ CD45RA- CD8+ T cell %CD8+ T cell | Inverse variance weighted | 3 | 8.09E-01 | 1.04 (0.76 -1.43 ) |
|  | CD25++ CD45RA- CD4 not regulatory T cell %CD4+ T cell | Inverse variance weighted | 3 | 8.11E-01 | 0.97 (0.77 -1.23 ) |
|  | CD33 on Immature Myeloid-Derived Suppressor Cells | Inverse variance weighted | 6 | 8.14E-01 | 0.99 (0.87 -1.11 ) |
|  | CD33 on CD33dim HLA DR- | Inverse variance weighted | 6 | 8.14E-01 | 1.01 (0.91 -1.13 ) |
|  | CD62L- myeloid Dendritic Cell %Dendritic Cell | Inverse variance weighted | 4 | 8.17E-01 | 0.97 (0.76 -1.24 ) |
|  | Activated & resting CD4 regulatory T cell %CD4+ T cell | Inverse variance weighted | 3 | 8.18E-01 | 0.97 (0.77 -1.23 ) |
|  | CD39+ activated CD4 regulatory T cell Absolute Count | Inverse variance weighted | 4 | 8.19E-01 | 0.97 (0.72 -1.29 ) |
|  | CD28 on activated & secreting CD4 regulatory T cell | Wald ratio | 1 | 8.21E-01 | 0.94 (0.57 -1.56 ) |
|  | CD28 on activated CD4 regulatory T cell | Wald ratio | 1 | 8.21E-01 | 0.94 (0.53 -1.66 ) |
|  | CD4 on Central Memory CD4+ T cell | Inverse variance weighted | 2 | 8.22E-01 | 1.05 (0.70 -1.57 ) |
|  | HLA DR on CD33dim HLA DR+ CD11b+ | Inverse variance weighted | 4 | 8.27E-01 | 1.05 (0.70 -1.55 ) |
|  | CD4 on CD45RA+ CD4+ T cell | Inverse variance weighted | 2 | 8.27E-01 | 1.04 (0.72 -1.52 ) |
|  | CD25 on naive-mature B cell | Inverse variance weighted | 4 | 8.28E-01 | 0.96 (0.68 -1.37 ) |
|  | CD27 on switched memory B cell | Inverse variance weighted | 9 | 8.28E-01 | 0.98 (0.81 -1.18 ) |
|  | CD4 on CD39+ activated CD4 regulatory T cell | Inverse variance weighted | 3 | 8.30E-01 | 1.04 (0.70 -1.56 ) |
|  | IgD- CD27- B cell %B cell | Inverse variance weighted | 3 | 8.33E-01 | 1.04 (0.73 -1.48 ) |
|  | Effector Memory CD8+ T cell %T cell | Inverse variance weighted | 2 | 8.33E-01 | 1.03 (0.78 -1.37 ) |
|  | CD19 on naive-mature B cell | Inverse variance weighted | 2 | 8.35E-01 | 1.39 (0.06 -30.29 ) |
|  | CD4 on CD28+ CD4+ T cell | Inverse variance weighted | 2 | 8.35E-01 | 0.95 (0.56 -1.60 ) |
|  | CD28 on resting CD4 regulatory T cell | Wald ratio | 1 | 8.36E-01 | 0.96 (0.65 -1.42 ) |
|  | CD25++ CD45RA- CD4 not regulatory T cell Absolute Count | Inverse variance weighted | 2 | 8.37E-01 | 1.09 (0.50 -2.36 ) |
|  | FSC-A on myeloid Dendritic Cell | Inverse variance weighted | 3 | 8.39E-01 | 1.04 (0.73 -1.48 ) |
|  | CD38 on IgD+ CD38+ B cell | Inverse variance weighted | 3 | 8.40E-01 | 0.93 (0.44 -1.95 ) |
|  | CD8dim Natural Killer T %lymphocyte | Inverse variance weighted | 6 | 8.40E-01 | 1.03 (0.74 -1.44 ) |
|  | CD33dim HLA DR+ CD11b+ %CD33dim HLA DR+ | Inverse variance weighted | 3 | 8.48E-01 | 0.97 (0.74 -1.27 ) |
|  | CD33dim HLA DR+ CD11b- %CD33dim HLA DR+ | Inverse variance weighted | 3 | 8.48E-01 | 1.03 (0.78 -1.34 ) |
|  | CD27 on memory B cell | Inverse variance weighted | 7 | 8.48E-01 | 0.98 (0.77 -1.24 ) |
|  | HLA DR+ T cell Absolute Count | Inverse variance weighted | 3 | 8.49E-01 | 1.06 (0.59 -1.91 ) |
|  | SSC-A on monocyte | Inverse variance weighted | 7 | 8.49E-01 | 1.02 (0.84 -1.24 ) |
|  | CD64 on CD14+ CD16- monocyte | Inverse variance weighted | 8 | 8.51E-01 | 1.02 (0.81 -1.29 ) |
|  | CD64 on monocyte | Inverse variance weighted | 8 | 8.51E-01 | 1.02 (0.80 -1.31 ) |
|  | CD19 on unswitched memory B cell | Inverse variance weighted | 4 | 8.53E-01 | 0.96 (0.65 -1.42 ) |
|  | CD28 on secreting CD4 regulatory T cell | Inverse variance weighted | 2 | 8.54E-01 | 0.96 (0.62 -1.48 ) |
|  | FSC-A on CD8+ T cell | Wald ratio | 1 | 8.55E-01 | 1.18 (0.20 -6.96 ) |
|  | Effector Memory CD4-CD8- T cell %T cell | Inverse variance weighted | 3 | 8.58E-01 | 0.95 (0.56 -1.62 ) |
|  | CD25 on IgD- CD27- B cell | Inverse variance weighted | 2 | 8.60E-01 | 1.05 (0.60 -1.85 ) |
|  | Naive CD8+ T cell %CD8+ T cell | Wald ratio | 1 | 8.62E-01 | 1.12 (0.31 -4.09 ) |
|  | CD4-CD8- Natural Killer T %lymphocyte | Inverse variance weighted | 6 | 8.65E-01 | 1.03 (0.73 -1.45 ) |
|  | CD20 on IgD- CD38dim B cell | Inverse variance weighted | 7 | 8.69E-01 | 1.03 (0.74 -1.42 ) |
|  | CD25 on IgD- CD24- B cell | Inverse variance weighted | 2 | 8.69E-01 | 0.94 (0.43 -2.03 ) |
|  | Resting CD4 regulatory T cell %CD4+ T cell | Inverse variance weighted | 5 | 8.72E-01 | 0.99 (0.85 -1.15 ) |
|  | Effector Memory CD4-CD8- T cell Absolute Count | Inverse variance weighted | 4 | 8.73E-01 | 0.96 (0.60 -1.54 ) |
|  | CD19 on switched memory B cell | Inverse variance weighted | 2 | 8.76E-01 | 0.93 (0.35 -2.45 ) |
|  | FSC-A on monocyte | Inverse variance weighted | 2 | 8.77E-01 | 1.06 (0.52 -2.14 ) |
|  | CD62L on CD62L+ Dendritic Cell | Inverse variance weighted | 2 | 8.77E-01 | 0.96 (0.55 -1.67 ) |
|  | CD24 on switched memory B cell | Inverse variance weighted | 2 | 8.81E-01 | 1.04 (0.65 -1.64 ) |
|  | PDL-1 on CD14- CD16+ monocyte | Inverse variance weighted | 2 | 8.83E-01 | 0.96 (0.56 -1.64 ) |
|  | CD24 on memory B cell | Inverse variance weighted | 2 | 8.85E-01 | 1.03 (0.66 -1.61 ) |
|  | CD19 on IgD+ CD38dim B cell | Inverse variance weighted | 4 | 8.87E-01 | 0.97 (0.65 -1.45 ) |
|  | CD19 on transitional B cell | Inverse variance weighted | 4 | 8.90E-01 | 0.97 (0.67 -1.42 ) |
|  | Effector Memory CD4+ T cell %CD4+ T cell | Inverse variance weighted | 2 | 8.90E-01 | 1.09 (0.34 -3.48 ) |
|  | CD8 on naive CD8+ T cell | Wald ratio | 1 | 8.92E-01 | 1.03 (0.72 -1.47 ) |
|  | CD45RA+ CD8+ T cell %CD8+ T cell | Inverse variance weighted | 4 | 8.93E-01 | 0.99 (0.90 -1.10 ) |
|  | Natural Killer T Absolute Count | Inverse variance weighted | 5 | 8.94E-01 | 0.97 (0.65 -1.47 ) |
|  | CD33+ HLA DR+ CD14- %CD33+ HLA DR+ | Wald ratio | 1 | 8.94E-01 | 0.97 (0.62 -1.52 ) |
|  | CD33+ HLA DR+ Absolute Count | Wald ratio | 1 | 9.04E-01 | 1.02 (0.70 -1.50 ) |
|  | CD33+ HLA DR+ CD14- Absolute Count | Wald ratio | 1 | 9.04E-01 | 1.02 (0.71 -1.48 ) |
|  | CD8dim Natural Killer T %T cell | Inverse variance weighted | 5 | 9.06E-01 | 1.02 (0.72 -1.44 ) |
|  | CD19 on IgD+ CD38+ B cell | Inverse variance weighted | 4 | 9.07E-01 | 0.98 (0.70 -1.38 ) |
|  | CD33 on CD14+ monocyte | Inverse variance weighted | 5 | 9.07E-01 | 0.99 (0.89 -1.11 ) |
|  | Effector Memory CD4+ T cell %T cell | Inverse variance weighted | 4 | 9.09E-01 | 0.96 (0.51 -1.82 ) |
|  | Effector Memory CD8+ T cell Absolute Count | Inverse variance weighted | 2 | 9.14E-01 | 0.98 (0.74 -1.30 ) |
|  | PDL-1 on CD14- CD16- | Wald ratio | 1 | 9.15E-01 | 0.94 (0.31 -2.86 ) |
|  | Naive CD4-CD8- T cell %T cell | Wald ratio | 1 | 9.16E-01 | 1.06 (0.39 -2.87 ) |
|  | CD25 on IgD+ CD38dim B cell | Inverse variance weighted | 2 | 9.17E-01 | 0.97 (0.56 -1.68 ) |
|  | CD8 on HLA DR+ CD8+ T cell | Inverse variance weighted | 2 | 9.17E-01 | 0.97 (0.55 -1.71 ) |
|  | CD28 on CD28+ CD45RA+ CD8+ T cell | Inverse variance weighted | 3 | 9.19E-01 | 0.98 (0.70 -1.38 ) |
|  | CD19 on IgD+ CD24+ B cell | Inverse variance weighted | 4 | 9.20E-01 | 0.98 (0.67 -1.44 ) |
|  | SSC-A on lymphocyte | Wald ratio | 1 | 9.21E-01 | 0.96 (0.43 -2.15 ) |
|  | CD33 on CD66b++ myeloid cell | Inverse variance weighted | 4 | 9.26E-01 | 0.99 (0.87 -1.14 ) |
|  | CD4+/CD8+ T cell | Wald ratio | 1 | 9.29E-01 | 0.94 (0.21 -4.10 ) |
|  | CD8+ T cell %T cell | Wald ratio | 1 | 9.29E-01 | 1.07 (0.25 -4.58 ) |
|  | Secreting CD4 regulatory T cell %CD4+ T cell | Inverse variance weighted | 7 | 9.30E-01 | 1.01 (0.88 -1.15 ) |
|  | CD33 on CD33dim HLA DR+ CD11b+ | Inverse variance weighted | 6 | 9.30E-01 | 1.00 (0.90 -1.12 ) |
|  | CD27 on IgD- CD38+ B cell | Inverse variance weighted | 2 | 9.32E-01 | 1.03 (0.54 -1.98 ) |
|  | CD25 on IgD+ B cell | Inverse variance weighted | 2 | 9.32E-01 | 0.98 (0.61 -1.57 ) |
|  | HLA DR++ monocyte %monocyte | Inverse variance weighted | 4 | 9.36E-01 | 1.01 (0.74 -1.39 ) |
|  | Activated & resting CD4 regulatory T cell Absolute Count | Wald ratio | 1 | 9.37E-01 | 0.96 (0.39 -2.36 ) |
|  | Central Memory CD4+ T cell %CD4+ T cell | Wald ratio | 1 | 9.37E-01 | 1.03 (0.47 -2.26 ) |
|  | Resting CD4 regulatory T cell Absolute Count | Inverse variance weighted | 4 | 9.38E-01 | 0.99 (0.78 -1.26 ) |
|  | CD28+ CD45RA- CD8+ T cell %T cell | Inverse variance weighted | 3 | 9.38E-01 | 1.01 (0.82 -1.24 ) |
|  | Plasmacytoid Dendritic Cell Absolute Count | Inverse variance weighted | 2 | 9.39E-01 | 0.94 (0.18 -4.91 ) |
|  | FSC-A on HLA DR+ Natural Killer | Inverse variance weighted | 3 | 9.44E-01 | 0.99 (0.70 -1.39 ) |
|  | CD33 on CD33+ HLA DR+ CD14- | Inverse variance weighted | 4 | 9.45E-01 | 1.00 (0.90 -1.12 ) |
|  | CD33 on CD33+ HLA DR+ CD14dim | Inverse variance weighted | 4 | 9.46E-01 | 1.00 (0.90 -1.12 ) |
|  | CD33 on CD33+ HLA DR+ | Inverse variance weighted | 4 | 9.48E-01 | 1.00 (0.90 -1.12 ) |
|  | B cell %CD3- lymphocyte | Inverse variance weighted | 3 | 9.48E-01 | 1.01 (0.75 -1.36 ) |
|  | CD33 on Monocytic Myeloid-Derived Suppressor Cells | Inverse variance weighted | 4 | 9.48E-01 | 1.00 (0.90 -1.12 ) |
|  | CD28+ CD45RA+ CD8+ T cell %CD8+ T cell | Wald ratio | 1 | 9.52E-01 | 0.99 (0.63 -1.56 ) |
|  | HLA DR on CD33dim HLA DR+ CD11b- | Inverse variance weighted | 5 | 9.53E-01 | 0.99 (0.70 -1.40 ) |
|  | HLA DR+ CD4+ T cell Absolute Count | Inverse variance weighted | 2 | 9.59E-01 | 0.97 (0.31 -3.04 ) |
|  | Naive CD4+ T cell %CD4+ T cell | Inverse variance weighted | 4 | 9.59E-01 | 1.02 (0.55 -1.89 ) |
|  | CD27 on IgD+ CD38- unswitched memory B cell | Inverse variance weighted | 5 | 9.60E-01 | 1.01 (0.82 -1.24 ) |
|  | CD16-CD56 on Natural Killer | Inverse variance weighted | 8 | 9.62E-01 | 1.01 (0.80 -1.27 ) |
|  | HLA DR+ CD4+ T cell %lymphocyte | Inverse variance weighted | 2 | 9.63E-01 | 1.03 (0.35 -3.02 ) |
|  | Terminally Differentiated CD8+ T cell Absolute Count | Wald ratio | 1 | 9.69E-01 | 0.98 (0.33 -2.93 ) |
|  | CD20 on IgD- CD27- B cell | Inverse variance weighted | 4 | 9.69E-01 | 0.99 (0.63 -1.57 ) |
|  | SSC-A on CD14+ monocyte | Inverse variance weighted | 5 | 9.71E-01 | 1.00 (0.79 -1.28 ) |
|  | CD8dim Natural Killer T Absolute Count | Inverse variance weighted | 5 | 9.76E-01 | 1.01 (0.69 -1.46 ) |
|  | CD3 on CD4+ T cell | Inverse variance weighted | 2 | 9.76E-01 | 0.99 (0.63 -1.57 ) |
|  | CD86 on CD62L+ myeloid Dendritic Cell | Wald ratio | 1 | 9.77E-01 | 1.01 (0.71 -1.43 ) |
|  | CD27 on IgD- CD38- B cell | Inverse variance weighted | 7 | 9.78E-01 | 1.00 (0.79 -1.27 ) |
|  | CD62L- myeloid Dendritic Cell Absolute Count | Inverse variance weighted | 5 | 9.78E-01 | 1.00 (0.78 -1.27 ) |
|  | Hematopoietic Stem Cell Absolute Count | Inverse variance weighted | 2 | 9.83E-01 | 1.00 (0.76 -1.31 ) |
|  | CD4-CD8- Natural Killer T %T cell | Inverse variance weighted | 5 | 9.84E-01 | 1.00 (0.71 -1.42 ) |
|  | CD14+ CD16+ monocyte Absolute Count | Inverse variance weighted | 3 | 9.88E-01 | 1.00 (0.73 -1.36 ) |
|  | CD86 on granulocyte | Inverse variance weighted | 2 | 9.90E-01 | 0.99 (0.35 -2.83 ) |
|  | CD19 on IgD+ CD24- B cell | Inverse variance weighted | 4 | 9.92E-01 | 1.00 (0.58 -1.72 ) |
|  | CD14+ CD16+ monocyte %monocyte | Inverse variance weighted | 3 | 9.94E-01 | 1.00 (0.75 -1.33 ) |
|  | IgD+ CD38- B cell %lymphocyte | Inverse variance weighted | 2 | 9.97E-01 | 1.00 (0.21 -4.86 ) |

**Figure S1. Instrumental variable (IV) assumptions of Mendelian randomization.**


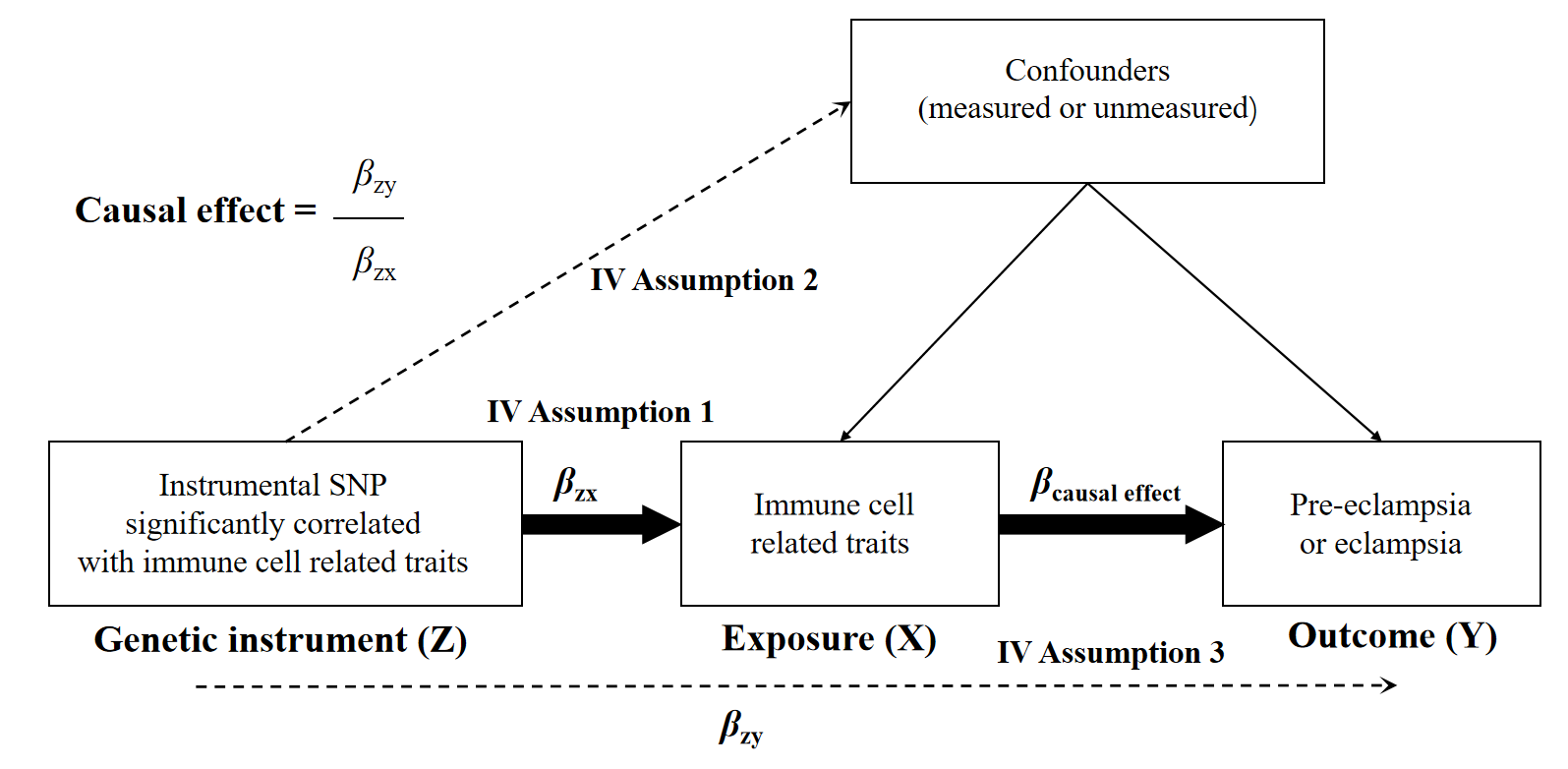

Supplement: Supplementary Materials — Table S1: patient demographic information from SardiNIA. Table S2: statistical power calculation for MR analysis. SNP: single nucleotide polymorphism; OR: odds ratio. Table S3: two-sample MR estimations showing the effects of immune cell traits on the risk of pre-eclampsia/eclampsia. Table S4: two-sample MR estimations showing the effects of immune cell traits on the risk of pre-eclampsia. Table S5: two-sample MR estimations showing the effects of immune cell traits on the risk of eclampsia. Figure S1: IV assumptions for MR. [file 8834312.f1.docx]
